# Re-investigating the structure–property relationship of the solid electrolytes Li _3−*x*_In_1−*x*_Zr_*x*_Cl_6_ and the impact of In–Zr(iv) substitution†

**DOI:** 10.1039/d2ta08433c

**Published:** 2023-01-25

**Authors:** Eveline van der Maas, Theodosios Famprikis, Saskia Pieters, Jonas P. Dijkstra, Zhaolong Li, Steven R. Parnell, Ronald I. Smith, Ernst R. H. van Eck, Swapna Ganapathy, Marnix Wagemaker

**Affiliations:** a Department of Radiation Science and Technology, Delft University of Technology Delft Netherlands s.ganapathy@tudelft.nl m.wagemaker@tudelft.nl; b Magnetic Resonance Research Center, Radboud University Nijmegen Netherlands erhve@nmr.ru.nl; c ISIS Facility, Rutherford Appleton Laboratory Chilton Didcot Oxfordshire UK OX11 0QX

## Abstract

Chloride-based solid electrolytes are considered interesting candidates for catholytes in all-solid-state batteries due to their high electrochemical stability, which allows the use of high-voltage cathodes without protective coatings. Aliovalent Zr(iv) substitution is a widely applicable strategy to increase the ionic conductivity of Li_3_M(iii)Cl_6_ solid electrolytes. In this study, we investigate how Zr(iv) substitution affects the structure and ion conduction in Li_3−*x*_In_1−*x*_Zr_*x*_Cl_6_ (0 ≤ *x* ≤ 0.5). Rietveld refinement using both X-ray and neutron diffraction is used to make a structural model based on two sets of scattering contrasts. AC-impedance measurements and solid-state NMR relaxometry measurements at multiple Larmor frequencies are used to study the Li-ion dynamics. In this manner the diffusion mechanism and its correlation with the structure are explored and compared to previous studies, advancing the understanding of these complex and difficult to characterize materials. It is found that the diffusion in Li_3_InCl_6_ is most likely anisotropic considering the crystal structure and two distinct jump processes found by solid-state NMR. Zr-substitution improves ionic conductivity by tuning the charge carrier concentration, accompanied by small changes in the crystal structure which affect ion transport on short timescales, likely reducing the anisotropy.

## Abstract 2

Aliovalent Zr(iv) doping in halide solid-state electrolyte Li_3_InCl_6_: combined X-ray and neutron diffraction, AC-impedance and solid-state NMR reveal structure and diffusion pathways.

## Graphical abstract

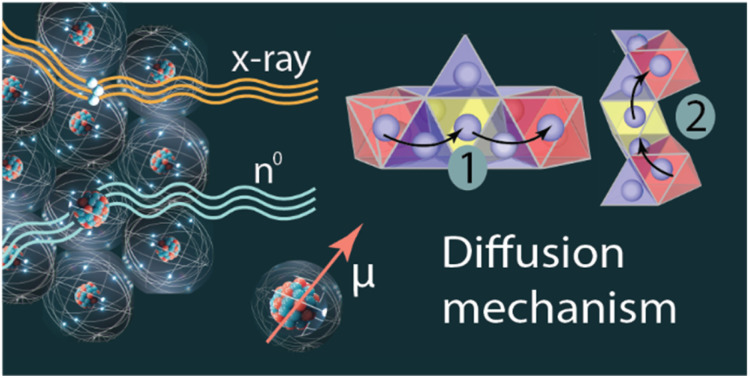

## Introduction

Replacing flammable, liquid electrolytes with ion-conducting solids can lead to advances in the safety of high-energy-density batteries.^1^ Many solid electrolyte materials with room-temperature ionic conductivity of the order of mS cm^−1^ have been developed in recent years. These include the families of the oxides (*e.g.* garnets^2^), phosphates (*e.g.* NaSICON^3^), sulfides (*e.g.* argyrodites Li_6_PS_5_X (X = Cl, Br, I^4^), Li_10_GeP_2_S_12_-type^5^) or hydrides (*e.g.* borohydrides/closoboranes^6^). For solid electrolytes to become a safer and feasible alternative, all-solid-state batteries need to be able to compete with liquid-electrolyte-based batteries both in terms of energy density and charging rates. Therefore, the amount of solid electrolyte has to be minimized both in the separator as well as in the cathode composite, without compromising on the internal resistance and electrode loading. This necessitates a solid electrolyte with a high ionic conductivity. The Li-ion conducting solid electrolytes with the highest conductivity so far are the sulfide-based ones, that can reach 10^−2^ S cm^−1^.^4,5^

Halide solid electrolytes with composition Li_3_M(iii)X_6_ (M(iii) = lanthanides, Sc, In, X = Cl, Br, I) have been receiving renewed interest from the scientific community since 2018, after Asano *et al.* published that Li_3_YCl_6_ and Li_3_YBr_6_ could reach ionic conductivities of 10^−3^ S cm^−1^ and showcased good performance in batteries using standard electrodes.^7^ Improved fundamental understanding of the conduction mechanism in Li_3_M(iii)Cl_6_ halide solid electrolytes may help formulate design guidelines to attain comparable ionic conductivities.

An interesting factor to understand, especially for solid-electrolytes used in cathode composites, is anisotropy of the diffusion or in its extreme case reduced dimensionality, which may arise for certain crystal structures. While one-dimensional diffusion is typically not favourable for long-range ion conduction, two-dimensional conductors need favourable microstructure for good percolation.^8^ Examples for anisotropy/reduced dimensionlity in layered (Li-)ion conducting materials are manifold, as for example graphite,^9^ LiCoO_2_,^10^ LiFePO_4_,^11^ TiO_2_–B,^12^ Li in hexagonal TiS_2_,^13^ hectorite-type silicate^14^ and β-alumina.^15^ Considering that crystallites of solid electrolytes like the monoclinic Li_3_InCl_6_, which has a layered atomic arrangement, may grow into platelets and may have anisotropic properties, this could very well impact the efficacy of ionic transport in composites, especially in combination with layered oxide cathodes.

Compared to most other relevant solid electrolyte families, chlorides have a higher oxidative stability and are compatible with 4 V cathodes.^16–18^ This is an important property, as otherwise electronically insulating coatings have to be utilized to protect the electrolyte from the high potential of the cathode. In this context, it was recently demonstrated that a spinel Li_2_In_*x*_Sc_2/3−*x*_Cl_4_ (0  ≤  *x*  ≤  0.666) solid electrolyte, in combination with an uncoated LiNi_0.85_Co_0.1_Mn_0.05_O_2,_ cathode achieved more than 3000 cycles with 80% capacity retention (cycled between 2.8–4.3 V *vs.* Li/Li^+^),^17^ which represents a milestone in the development of all-solid state batteries. The compatibility of In-, Sc-, Zr- and Y-containing chloride solid electrolytes with high-voltage cathodes and the corresponding evolution of the all solid-state battery impedance have been thoroughly investigated by Kochetkov *et al.*^19^

A commonly employed strategy to increase the ionic conductivity of a material is through aliovalent substitution. In aliovalent substitution, an atom is replaced with another of different charge, which is compensated by a change in the charge carrier concentration. In most Li-ion conducting materials, an optimum Li^+^/V_Li_ charge carrier concentration exists: at higher Li^+^ concentrations, more Li^+^ is available in the electrolyte to transport the charge. At too high Li^+^ concentrations, the probability of a Li^+^ having access to a neighbouring vacancy is reduced, which can become the rate-limiting factor and reduce ionic conductivity. For the chloride Li_3_M(iii)Cl_6_, this optimum was successfully achieved by Zr(iv) substitution for M(iii) = Y (ref. 20), Er (ref. 20 and 21), Yb (ref. 22–24), In (ref. 25–27) and Sc (ref. 27). The Zr(iv) replaces the M(iii), and the excess charge is compensated by a lithium vacancy. In all structures, the ionic conductivity measured by impedance spectroscopy has been shown to increase up to a certain substituent concentration.^18–25^

When the Li_3_M(iii)Cl_6_ materials were first investigated, a trend between the M(iii) Shannon radius and the crystal structure was found. Larger M(iii) would crystalize in trigonal *P*3̄*m*1 (M(iii) = Y, Tb-Tm),^28^ intermediate size in orthorhombic *Pnma* (Yb, Lu)^28^ and even smaller (M(iii) = In, Sc) in monoclinic *C*2/*m*.^29^ This still holds under the same synthesis conditions, but different synthesis methods have shown to be able to disturb this trend.^30–32^

For the materials with M(iii) = Y (ref. 20), Er (ref. 20), Yb (ref. 22), a trigonal *P*3̄*m*1 to orthorhombic *Pnma* phase transition has been observed upon Zr-substitution. This is rationalized by the reduction of the average transition metal radius^20^ by introducing the smaller Zr(iv)-ion (the Shannon radius of Zr is 0.72 Å in 6-fold coordination, compared to Y (0.90 Å), Er (0.89 Å) and Yb (0.87 Å)).^33^ The *Pnma* phase can also be synthesized without Zr-substitution, by synthesizing the material by co-melting of the reagents with small LiCl deficiency.^30^

For Li_3_M(iii)Cl_6_ with smaller M(iii), such as In (0.80 Å) and Sc (0.75 Å), the structure crystallizes in monoclinic *C*2/*m*.^25,34^ The end member Li_2_ZrCl_6_ also crystallizes in monoclinic *C*2/*m*^25,34^ (isostructural to Li_3_InCl_6_) when synthesized with conventional ampoule synthesis, as well as in trigonal *P*3̄*m*1 when synthesized mechanochemically.^28^ In both cases, substitution with M(iii) = In (ref. 27 and 31, trigonal structure can exist below 40% In) and Fe (trigonal structure only, ref. 34, solubility up to 25% Fe-content) were shown to improve ionic conductivity, though to a lesser extent than the In-rich compositions. Mixtures between the larger and smaller M(iii) can be synthesized, and the resulting crystal structure also seems to depend on the average M(iii) radius, as also observed for Zr-doping and demonstrated by the Li–Y–In–Cl solid electrolytes investigated by Li *et al.*^35^

**Fig. 1:**
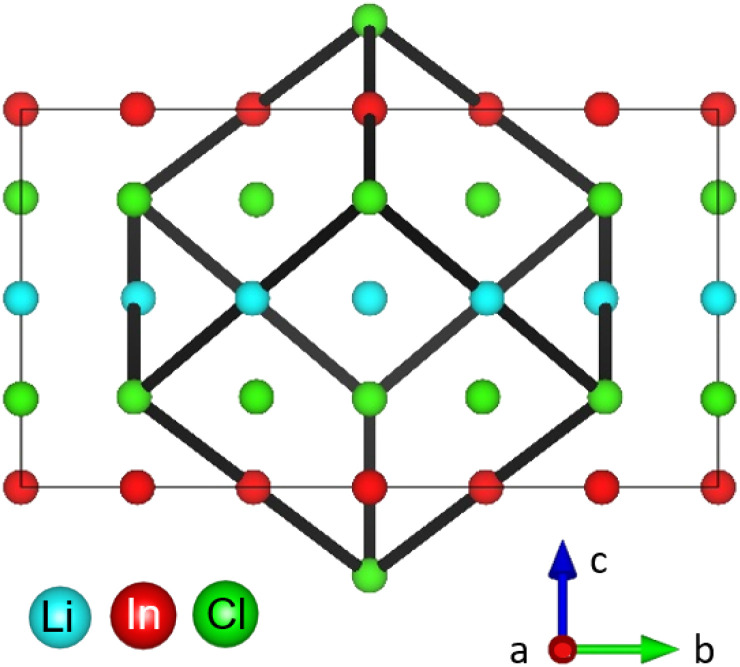
Simplified crystal structure of Li_3_InCl_6_. The structure can be considered as a double LiCl unit cell with a small monoclinic distortion and Li replaced by In(iii) and vacancies (the cubic cell is illustrated by the black cube). Most cations are on octahedrally coordinated sites within the Cl cubic close packing arranged in a layered fashion in alternating Li- and In/Li layers.

The monoclinic structure of the Li_3_InCl_6_ consists of a double LiCl unit cell with a small monoclinic distortion and part of the Li^+^ replaced by In^3+^ in a layered arrangement (see Fig. 1). In this structure, Zr(iv) substitution does not lead to a phase transition.^25^ While the monoclinic structure type is less common in chlorides, similar structures are found for bromide and iodide analogues.^36–39^

Upon Zr-substitution, the changes in lattice parameter are small^25^ and the anionic framework remains quasi cubic-close-packed. It is therefore unlikely that the increase in ionic conductivity arises from a change in the size of the bottleneck or interaction of the Li-ions with the anionic lattice. This raises the question: To what extent is the increase in ionic conductivity on Zr(iv) substitution due to the change in charge carrier concentrations, changes in the crystal structure and/or changes in the diffusion pathway(s)?

In a recent study,^25^ this question was investigated using a structural model obtained from Rietveld refinement based on neutron diffraction data alone, bond valence calculations, AC-impedance measurements as well as solid-state NMR measurements. It was concluded that the occupancy of the secondary In-site, which breaks the layered arrangement of the Li-atoms in the *ab*-plane, promotes ion conduction also along the *c*-direction. While the bond valence calculations show similar activation energies for all possible jump pathways, the solid-state NMR measurements for Li_3_InCl_6_ indicate two motional modes, rendering the picture unclear.

In ref. 27, a similar study was performed for the same substitution series. In this case, bond valence calculations, also based on structural models obtained from neutron diffraction alone, show some difference in the activation energies for both diffusion pathways (0.483 eV and 0.456 eV for paths along the *ab*-plane, and 0.509 eV along the *c*-direction). Therefore, it was suggested that though 3D diffusion is possible, the diffusion is likely faster in the *ab*-plane and therefore anisotropic, however no experimental data on short-range ion transport was presented.

Finally, in ref. 40, the Li_3−*x*_In_1−*x*_Zr_*x*_Cl_6_ substitution series is investigated by *ab initio* molecular dynamics simulations. An increase in conductivity from 1.2 to 22 mS cm^−1^ has been found for a Zr concentration of 25%. These simulations reproduce published experimental trends^25^ as well as the trends found in the present work, albeit with a larger magnitude and high error bar for the tracer diffusion coefficient, probably because of the challenge of simulating such disordered materials (*i.e.* featuring partially occupied and/or shared atomic positions) with the number of atoms that are usually simulated by *ab initio* methods.

In this contribution, we re-investigate the structure–property relationship of the Li_3_InCl_6_ solid electrolyte and the impact on it of Zr(iv) substitution, exclusively using experimental techniques. To do so, the series Li_3−*x*_In_1−*x*_Zr_*x*_Cl_6_ (*x* = [0, 0.5], steps of 0.1) was synthesized. Combined Rietveld refinement using X-ray and neutron powder diffraction data was employed for the structural characterization. The long-range ionic conductivity was measured using AC-impedance spectroscopy. Information about the ionic conductivity at short timescales were obtained from solid-state NMR spin-lattice relaxometry measurements. The measurements were performed at multiple Larmor frequencies, to be able to better distinguish model suitability and to resolve different motional modes. Finally, the static NMR-lineshape at high temperature was analysed, providing information on the ionic motion at longer timescales.^41^

Owing to the combined structural refinement using X-ray and neutron diffraction data, we can report accurate atomic occupancies which reveals clear trends upon Zr substitution. The solid-state NMR measurements at multiple fields allow us to resolve and fit the two motional modes in Li_3_InCl_6_ mentioned by ref. 25, which we demonstrate to be distinct in their activation energy. The second mode is not apparent for the doped materials, and the two modes may have approached each other as indicated in ref. 25, which correlates with the reduction of the In-occupancy in the *c*-direction. For the doped materials Li_3−*x*_In_1−*x*_Zr_*x*_Cl_6_ with *x* = 0.3 and *x* = 0.5, we show that unambiguous fitting of spectral densities is a very complex problem, emphasizing that results need to be interpreted with care. We find residual chemical shift anisotropy, represented by asymmetric satellites, in the static line shapes at high temperatures. This means that the motion does not fully average out the chemical shift across all sites, indicating that there are differences in the jump-rates across different sites at the timescale of T_2_, which, considering the crystal structure, we interpret as anisotropic ion conduction.

## Results and discussion

**Fig. 2:**
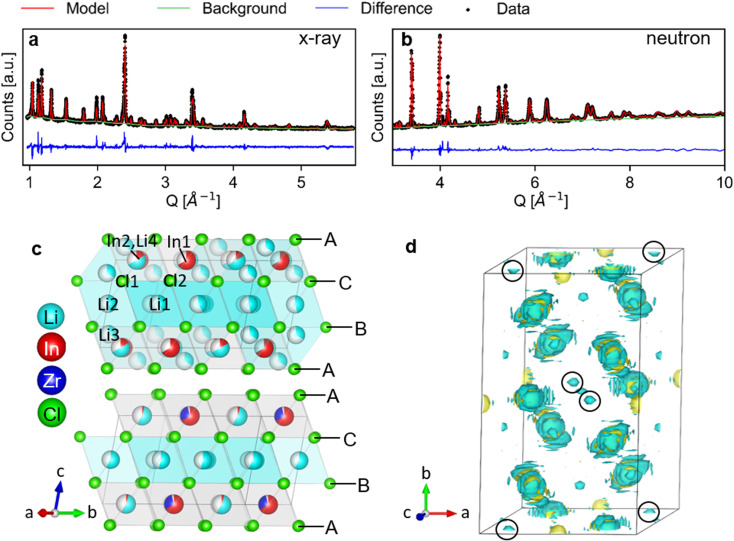
Simultaneous Rietveld fit to the (a) X-ray diffraction data and (b) neutron diffraction data of Li_2.7_In _0.7_Zr_0.3_Cl_6_ in the *C*2/*m* space group. (c) The structure of Li_3_InCl_6_ (top) and Li_2.7_In _0.7_Zr_0.3_Cl_6_ (bottom), cif files are provided in the ESI files.† The anionic lattice is formed by cubic close packing of the chlorine atoms in Cl1 (8j) and Cl2 (4i). Li4 (4g) co-occupies the In2 (4g) site. The tetrahedral Li3 (8j) site could not be observed for dopant concentrations where *x* ≥ 0.3 in Li_3−*x*_In_1−*x*_Zr_*x*_Cl_6_. Upon substitution, Zr preferentially occupies the In1(2b) site and the In2(4g) occupancy is reduced. (d) Effective scattering length density map of the neutron diffraction data of Li_3_InCl_6_. Negative scattering length density is plotted in turquoise, positive scattering length density in yellow. The tetrahedral Li3 (8j) site is circled in black. For refinements using the other datasets, see ESI Fig. 2–6.†

The long-range structure of the samples was characterized by simultaneous Rietveld refinement using X-ray (Fig. 2a) and neutron (Fig. 2b) powder diffraction data (also see ESI Fig. S2–S6†). The materials crystallized in the monoclinic space group *C*2/*m* (12), consistent with previous work.^25,28^ The structure is based on cubic-close-packing of Cl anions. The cations occupy mostly octahedral sites and are arranged in layers (Fig. 2c). One layer is occupied by Li (between B and C), followed by a mixed cation Li/In/Zr layer (between layers A and C, A and B). The lattice parameter *a* showed a small increase (6.404–6.416 Å) with increasing Zr-dopant concentration, while *c* decreased (6.380–6.357 Å), which is consistent with literature^25^ (ESI Fig. S1†). The increase of the *a*-parameter could be related to the lower Li-content in the layer, leading to larger coulombic repulsion between the Cl atoms. The decrease in the *c*-parameter can be rationalized by the smaller size and higher charge of Zr compared to In, leading to a decrease in the interlayer distance. The angle beta showed only a slight increase (109.776–109.961°) while the lattice volume showed a maximum at *x* = 0.2, but with small absolute changes (within 1.5 Å^3^) (ESI Fig. S1†). For compositions with *x* = 0 and 0.2 we find the occupation of a tetrahedral site in the mixed cation layer (Li3 in Fig. 2c top image, in the M-layer). The occupancy of this site can be visualized from the effective neutron scattering-length-density map shown in Fig. 2d. An effective scattering length density map is the Fourier transform of the diffracted signal, combining the intensity information from the measurement and the phase information from the model obtained from Rietveld refinement. This is the neutron-equivalent of the electron density maps from X-ray diffraction data. Our measurements further show that the tetrahedral site disappears at higher dopant concentrations (*x* ≥ 0.3).

**Fig. 3:**
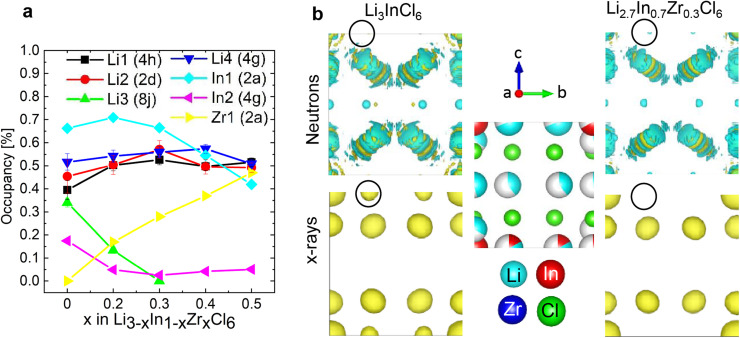
(a)Trends of the site occupancies as a function of the composition. (b) Effective scattering length density maps calculated from the neutron diffraction data (top; negative density in blue and positive density in yellow) and electron density maps calculated from the X-ray diffraction data (bottom; positive density in yellow). The unit cell was cut in half along the mirror plane, as visible in the atomic arrangement in the middle. The black circles are around the In2/Li4 (4g) site, illustrating that the opposite scattering length of the Li and the In cancel out the neutron signal for Li_3_InCl_6_ on that site.

To increase the accuracy of the refinement, elemental compositions measured with inductively-coupled-plasma optical emission spectroscopy (ICP-OES) (ESI Table S1†) were added as constraints to the model. The composition was calculated using charge neutrality and assuming all Cl-positions to be fully occupied. The Rietveld refinement based on the neutron diffraction data alone was not stable, despite good contrast between the elements. This difficulty is partly due to the opposite sign of the coherent scattering length of Li and In, as can be seen from the effective scattering length density maps (Fig. 3b). Li has a negative scattering length (*b*_Li_ = −1.9 fm) about half the magnitude of that of In (*b*_In_ = 4.065 fm).^42^ A negative scattering length means that the scattered neutrons have a 180° phase shift relative to a positive scattering length, and hence the waves scattered from Li and In on the same site will interfere destructively, resulting in an effective scattering length of the site, which is weighed by the site occupancy. If the ratio of the occupancies of Li : In is –b_In_ : b_Li_ (approximately 2 : 1), the effective scattering length approaches zero and thus the site will result in no scattered intensity. The site also does not have to be fully occupied, leaving the refinement under-defined. It is therefore crucial to use an additional contrast (X-ray diffraction data) to accurately determine the crystal structure. X-ray diffraction is sensitive to the occupancy of In and Zr (due to the number of electrons), which can also be distinguished using neutrons due to the larger scattering length of Zr compared to In (7.16 compared to 4.065 fm), and the neutron diffraction data also adds information about the lithium occupancies thanks to the negative scattering length.

This synergy is illustrated in the effective scattering length density map of Fig. 3b. Looking at the (electron) density calculated from the X-ray diffraction data, we can clearly see density on the In2(4g) site for Li_3_InCl_6_. For the doped material Li_2.7_In_0.7_Zr_0.3_Cl_6_, no electron density is observed on the same site (at the same isosurface level). In the effective neutron scattering length density map from the corresponding materials, there is no effective density in Li_3_InCl_6_ and some negative effective scattering length density is observed for Li_2.7_In_0.7_Zr_0.3_Cl_6_. Using both contrasts in a simultaneous refinement, the occupancies of the sites converge, the results of which are shown in Fig. 3a.

The site occupancies show three clear trends. (1) The Zr only occupies the In1 (2b) site, and hence the Zr-occupancy of the site increases linearly as the total In-occupancy drops. (2) Already at small dopant concentrations, the In occupancy on the In2 (4g) site decreases and then stays low around 5%. The non-zero occupancy is clearly visible from the X-ray diffraction data of Li_3_InCl_6_, and was also observed previously from single crystal X-ray diffraction.^29^ (3) For the lithium sites, we observe an approximately linear decrease of the tetrahedral Li3 (8j) site occupancy, as *x* increases. This indicates that the tetrahedral site is a higher energy site as it is abandoned upon lowering the overall Li concentration, which is consistent with the fact that many related halide structures that occur in nature only feature Li with octahedral coordination, as for example all LiX (X = Cl, Br, I) binary halide salts.

The structure solution found here differs from other solutions published in literature.

• In line with previous studies,^25,27^ the present work indicates Li occupancy of a tetrahedral site, based on neutron diffraction data, however at different location.

• In previous studies,^25,27^ the Zr is reported to occupy both In sites, whereas in the present study it is only found on the In1 site.

• The In-occupancy of the In2–Li4 site in the present work is higher for Li_3_InCl_6_ and reduces upon the introduction of Zr, a trend that is not reported in previous studies.^25,27^

• The present results indicate that the occupancy of the tetrahedral Li-site disappears at 30% Zr content already, which only occurs at a Zr-content of 80% in ref. 27 and is not reported in ref. 25.

The discrepancies with models in literature can arise, in principle, from differences in synthesis and therewith actual differences in structural details (as for example reported in ref. 37 for trigonal Li_3_YCl_6_). In ref. 25 and 27, the materials are synthesized by ball-milling and a low-temperature annealing step at 260 °C. In this contribution, the materials were synthesized by an ampoule synthesis, with a short ball-milling step to get a fine powder, and another, shorter thermal step to obtain a more crystalline material for diffraction (after the ball milling, the ionic conductivity of the powder was much lower and the Bragg-peaks broader). The use of both neutron as well as X-ray diffraction data can be another reason for the differences in atomic occupancies, considering the aforementioned correlations when refining with neutron diffraction data only, especially for the In2–Li4 occupancies.

**Fig. 4:**
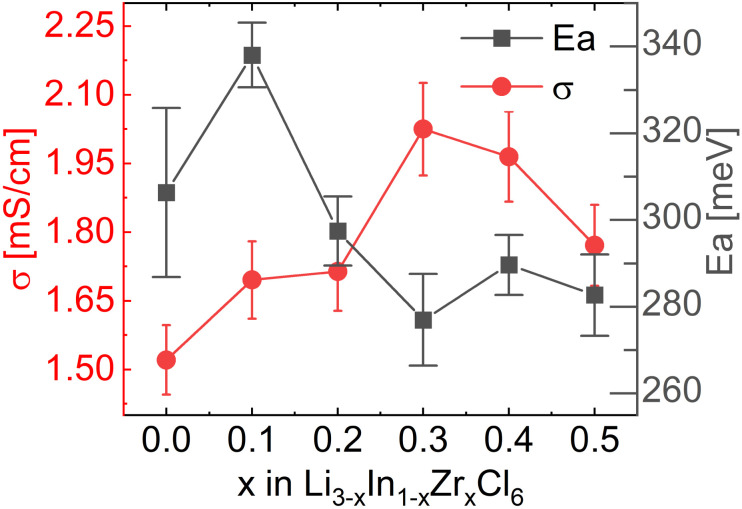
Ionic conductivities and activation energies extracted from the Arrhenius relationship obtained from AC-impedance spectroscopy measurements. For individual AC-impedance spectra fits, parameters and errors, see the report in the ESI files.† For the validity of the equivalent circuit models used, see the Kramers–Kronig relationship in ESI Fig. S7.† For the full Arrhenius diagrams, see ESI Fig. S8.† Errors of the ionic conductivity are estimated from Gaussian error propagation as suggested by Krasnikova *et al.*^43^ given in the ESI Table S2.† Errors in the activation energy errors are determined from the linear fit and given in the ESI Table S3.†

To measure the ionic conductivity with AC-impedance spectroscopy, the as-synthesized powders were cold-pressed into pellets. The ionic conductivity showed a maximum at *x* = 0.3 at 2.02 mS cm^−1^, an increase by a factor ∼1.6 compared to Li_3_InCl_6_ (1.52 mS cm^−1^) (Fig. 4; see ESI files† for the data, equivalent circuit model and fitted parameter with errors; see ESI Fig. S7† for the confirmation of validity of the equivalent circuits using the Kramers–Kronig relationship; see ESI Table S2† for error calculations). The maximum in ionic conductivity also corresponds with the lowest activation energy of 280 meV, though the differences in activation energy between the different compositions is small and not significant in most cases.

These values correspond remarkably well with values reported in ref. 27, who found 1.5 mS cm^−1^ for undoped Li_3_InCl_6_ and a maximum value of 2.1 mS cm^−1^ also at 30% Zr content. The conductivities found in ref. 25 are lower, with a maximum of 1.2 mS cm^−1^ found at 40% Zr content.

To obtain information about the dynamics at shorter timescales, we have conducted a series of solid-state NMR measurements. Spin-lattice relaxation measurements as a function of temperature can give information on timescales of the inverse of the Larmor frequency (*ω*_0_), which is in the order of 10^−9^ s for the presently applied fields. Such frequencies allow to probe diffusion on the scale of individual jump events, also including local back-and-forth hopping. This allows the separation of jump processes that differ significantly in frequency, the extraction of activation energies of jump processes (compared to activation energies of diffusion across a fabricated pellet, as in impedance), as well as providing insights into correlated diffusion. This is due to the fact that the temperature dependence of NMR relaxation rates depends on the Fourier transform of the correlation function of the diffusing atom usually referred to as the spectral density (see ESI Text 1†). A variety of spectral density functions can be found in literature,^44^ developed to represent different diffusion mechanisms.

In this work, four spectral densities were considered and compared:

(1) For uncorrelated three-dimensional motion, the Bloembergen, Purcell and Pound (BPP) spectral density function (BPP model) was developed^45^ in 1948. For BPP-type behaviour, the relaxation rates as a function of inverse temperature should show a symmetric curve with a maximum at the temperature where the hopping frequency is of the order of the Larmor frequency. Deviations from the BPP model lead to loss of symmetry between the high- and low-temperature limits.

(2) A semi-empirical model for 2D conduction has been derived by Richards^46^ by looking at the low- and high-temperature limits of the relaxation curve. Lower dimensional conduction affects the slope on temperatures above the maximum where *ω*_0_*τ_C_ ≪ 1.

(3) An empirical model was further developed for ion conduction in the layered sodium-ion conductor beta-alumina.^47,48^ This model is based on the BPP model, but introduces a fitting parameter *β*, to account for the deviation from the BPP model (see ESI Text 1†). The parameter has no physical basis.

(4) A similar parameter as in model (3) has been introduced to the semi-empirical model by Richards,^46^ to account for correlations between Coulomb interactions of the moving ion (see ESI Text 1†).

All of these spectral densities were tested for simultaneous fitting of the data measured at multiple Larmor frequencies in this work.

**Fig. 5:**
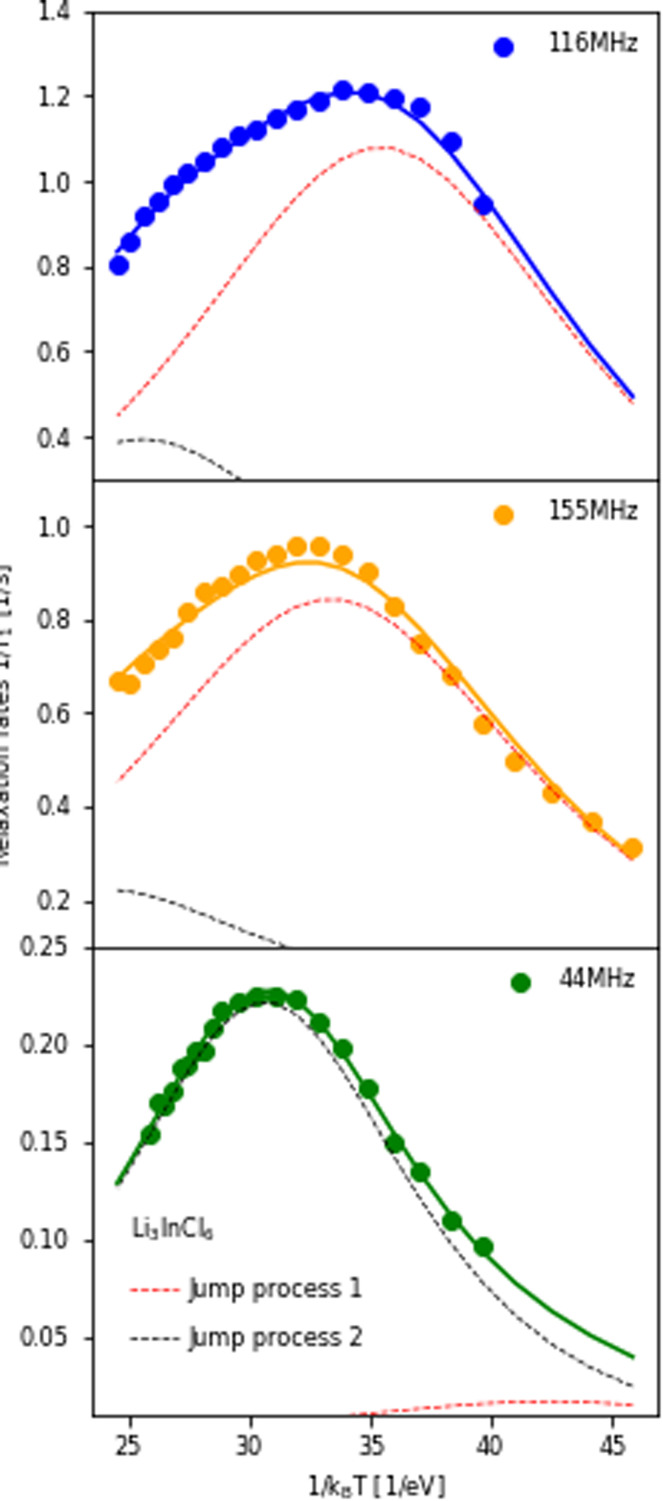
Relaxation rates of Li in Li_3_InCl_6_ measured at three different Larmor frequencies. 116 MHz corresponds to ^7^Li measured on a 300 MHz, 155 MHz to ^7^Li on a 400 MHz and 44 MHz to ^6^Li on a 300 MHz spectrometer. The fit of the relaxation rate (solid line) represents a model with two BPP-type jump processes, the Bayesian information criteria of this fit is −476.3 (compared to ∼−300 for other spectral densities, see ESI Fig. S10†). The contribution of the individual jump processes to the relaxation rate are shown in red and black (same processes in all plots, the sum of the two individual rates is the full rate) and illustrate which process the individual datasets are sensitive to. For the figure with logarithmic axis, see ESI Fig. S9.†

The temperature dependence of the ^6,7^Li spin-lattice relaxation times in Li_3_InCl_6_ was measured at three different Larmor frequencies as shown in Fig. 5. The dataset collected at the lowest Larmor frequency (*ω*_0_ = 44 MHz, ^6^Li on a 300 MHz spectrometer) shows a single maximum in the relaxation rate at a higher temperature than the measurements for ^7^Li, even though the Larmor frequency of ^6^Li is lower compared to ^7^Li. There is only one explanation for this *i.e.* the jump processes are not the same, suggesting multiple processes, as already suggested in ref. 25.

If the jump processes are (to some approximation) independent of each other, the relaxation rates (*R* = 1/*T*_1_) of the two processes are additive.^49^ Following this assumption, a model containing two BPP-type jump processes was constructed and fit to the three datasets simultaneously (Fig. 5), revealing activation energies of 0.189 eV for the slower jump process (Fig. 5, black dotted line) and 0.131 eV (Fig. 5, red dotted line) for the faster jump process. Comparison between fits using the standard spectral densities (1–4) are shown in the ESI Fig. S10.†

To investigate to what extent the two processes contribute to the total diffusivity, the diffusion constant was calculated for the distinct hopping process, assuming an octahedral–tetrahedral–octahedral (oct–tet–oct) diffusion path (see discussion below) with an average jump distance of 2.1 Å (similar to the procedure in ref. 50). The faster process (red line, *E*_a_ = 0.131 eV) (ESI Table S4†) leads to a diffusion coefficient of ∼10^−12^ m^2^ s^−1^ (ESI Table S6†), which is the same order of magnitude as the diffusivity calculated from impedance measurements using the Nernst–Einstein relationship (ESI Table S6†). The slower process (black line, *E*_a_ = 0.189 eV) leads to a diffusivity of the order ∼10^−13^ m^2^ s^−1^. Therefore, we can assume that the first jump process is the main contributor to macroscopic diffusion in Li_3_InCl_6_.

**Fig. 6:**
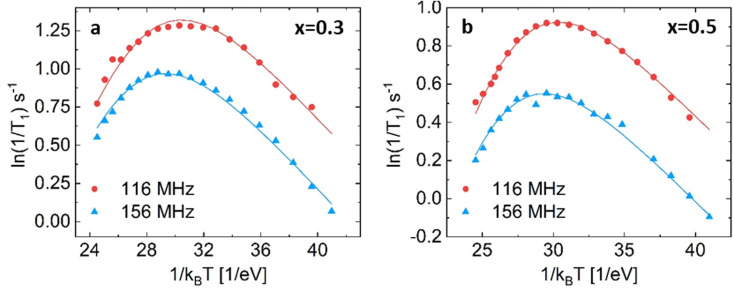
NMR relaxometry of Li_3−*x*_In_1−*x*_Zr_*x*_Cl *x* = 0.3 (a) and *x* = 0.5 (b) fit with spectral density (3), the empirically modified BPP model. For a comparison of how the different models fit the datasets, see ESI Fig. S11 and 12.† For the fitting parameters, see ESI Table S4.†

Both substituted materials with composition *x* = 0.3 and *x* = 0.5 (see Fig. 6) were best fit with the two empirical models ((3), (4) in the list above), which both fit the data almost equally well (Bayesian information criteria of −318 and −333, see Fig. S11 and S12†).

Spectral density (3) is the same spectral density as used in Helm *et al.* (see Table S4† for comparison of the obtained values).^25^ They reported a clear trend in the activation energies from the solid-state NMR spectral-density fitting, similar in shape to the trend observed from AC-impedance spectroscopy. Considering the high correlation among fitting parameter reproduced from their data (>|0.9|, see ESI Table S5†) and relatively few points on the high-temperature slope (Fig. S13†), the data does not contain the information needed to unambiguously fit activation energies (see the large difference in the values in ESI Table S5†).

In fact, even the regular BPP model (with one fitting parameter less), results in a very large absolute correlation between the pre-exponential factor and the activation energy (∼0.99) for the measurements in this work, indicating that the measurements should be performed for a large temperature range and at multiple Larmor frequencies for accurate determination of the correct spectral densities and activation energies. The development of a better method to estimate errors in these highly correlated systems is subject of future research.

For Li_3−*x*_In_1−*x*_Zr_*x*_Cl_6_, *x* = 0.3 the individual fits results in activation energies of 0.256 and 0.257 eV and *β* values of 0.365 and 0.427 respectively, whereas the combined fit yield an activation energy of 0.30 ± 0.02 eV and *β* of 0.29 ± 0.04. Despite the higher ionic conductivity measured from AC-impedance spectroscopy, the activation energy found at present is much higher (0.3 eV for *x* = 0.3) than the values found for the activation energies of unsubstituted Li_3_InCl_6_ (*E*_a_ = 0.131 for the fast process, 0.189 eV for the slow process).

In this case, the empirically modified spectral density that fits the observed relaxation rate well may lead to different activation energies than those found using the BPP model. It is not clear how the values obtained from these models can be compared, especially considering the possibility of multiple jump processes that can all assist total spin-lattice relaxation. Measuring at more Larmor frequencies or a larger temperature range may be beneficial, which is a subject of further study. From the data presented here, it can be concluded that the Zr-substituent also affects the high-frequency Li-ion motion, as indicated by the different shape of the relaxation curves.

The static NMR lineshape is already motionally narrowed at room temperature (ESI Fig. S14†). At higher temperatures sharp features appear in the satellites which can be fit by residual quadrupolar coupling (6 kHz) combined with chemical shift anisotropy (ESI Fig. S14,† span 3 ppm, skew *κ* of −0.76). Less pronounced but similar features are observed for the Zr-substituted samples (ESI Fig. S15†). This can be interpreted in two ways: either, the motion averages out the chemical shift anisotropy more effectively in the doped samples (but not fully), or this is an effect of the more disordered local environment due to the Zr-dopant.

**Fig. 7:**
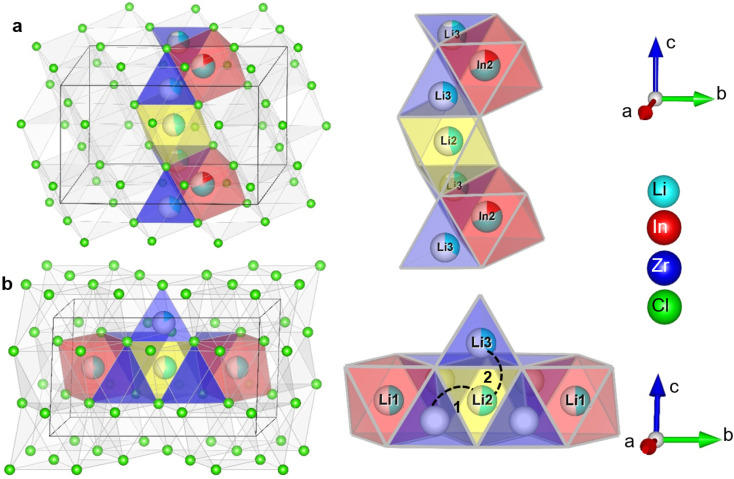
Illustration of the possible diffusion pathways in the crystal structure. (a) Diffusion along the *c*-direction involving the tetrahedral Li3 (8j) site the octahedral Li2 (2c) site and the octahedral In2/Li4 (4g) site (b) diffusion along the *ab* plane, illustrating the two different jump processes *i.e.* 1: jump through the empty tetrahedral site sharing faces with the lithium octahedral sites (Li1 (4i), Li2 (2c)); 2: jump between the Li2 (2c) octahedral site, the occupied tetrahedral Li3 (8j) site and can continue along the In2/Li4 site, as in (a). Diffusion in the M-layer in the *ab*-plane has similar possible diffusion paths, but with the In1 (2a) site blocking due to the high In/Zr occupancy, strongly reduces the number of free pathways.

It is interesting to relate these findings to the possible diffusion pathways in the crystal structure and structural changes as a function of the degree of substitution. In Fig. 7, two possible diffusion pathways have been illustrated. Both of them occur *via* a chain of face-sharing tetrahedral and octahedral sites, as is known to happen for cubic-close-packed lattices.^51^ One is along the *c*-direction, and involves the Li2 (2c) octahedra, the tetrahedral Li-site Li3(8j) observable from diffraction and the shared In2/Li4 (4g) octahedral site (Fig. 7a). The other path is along the Li-layer in the *ab*-plane (Fig. 7b), involving both octahedral Li-sites in the layer and vacant tetrahedrons.

**Fig. 8:**
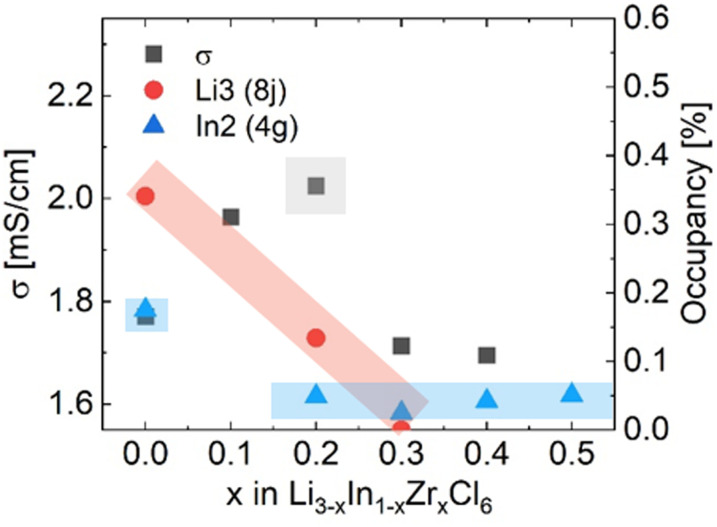
Comparison of the ionic conductivity *σ* as measured from impedance with the occupancies of the Li3 (8j) and In2 (4g) sites for Li_3−*x*_In_1−*x*_Zr_*x*_Cl_6_. The conductivity reaches its maximum as the tetrahedral Li3 (8j) site disappears. The occupancy of the In2 (4g) site drops to its minimum value already before the maximum in ionic conductivity is reached. Neutron diffraction data of the material with *x* = 0.1 was not measured due to limited beamtime.

Considering these two diffusion paths and comparing them to the structural changes found and the findings from AC-impedance (Fig. 8) and solid-state NMR spectroscopy, the following observations and considerations can be made.

(1) Pristine Li_3_InCl_6_ has the lowest ionic conductivity of the series, while it has the highest In2 (4g) site occupancy, the highest tetrahedral (8j) Li site occupancy and shows two jump processes as probed by solid-state NMR.

(2) The conduction in the *ab*-plane of the material (Fig. 7) occurs along oct–tet–oct paths, possible in both the Li- as well as the mixed cation layer. The large number of octahedra occupied by the In(iii) or Zr(iv) cations blocks conduction in the mixed cation layer, making long-range diffusion less probable. In addition, along the *c*-direction, there is a possible oct–tet–oct path, which connects the octahedra in the Li2(2c) octahedra with the tetrahedral Li3(8j) site and the In2/Li4 (4g) site. Due to the high cumulative occupancy of the In2/Li4 site (∼0.7), this path is also less probable compared to the oct–tet–oct in the Li-layer, (occupancies ∼0.4–0.45), because of the lower probability of having a vacant site to jump to. Specifically, all sites occupied by In can be considered as permanently blocking the conduction along the *c*-direction. From the activation energies of the spectral density fits (ESI Table S4†) the contribution to the diffusivity of both jump processes can be calculated (ESI Table S6†) and is found to be ∼1 : 10. Due to the lower probability of conduction in the *c*-direction, we infer that the faster diffusion process occurs in the *ab*-plane, and the slower process along the *c*-direction. Anisotropy of the diffusion is further supported by measurements of the static lineshapes (ESI Fig. S14 and S15†).

(3) Upon increasing amount of *x* in Li_3−*x*_In_1−*x*_Zr_*x*_Cl_6_, the In2(4g) site occupancy drops to ∼5%, after which it remains constant (Fig. 3a, Fig. 7). The Li that is removed for charge compensation is first removed from the tetrahedral site. These two trends, in principle, open up the path along the *c*-direction.

(4) From the NMR spectral density fits, the faster jump process (0.131 eV, red dotted line) observed in the pristine Li_3_InCl_6_ is not detected anymore for ^7^Li measured on the 300 MHZ spectrometer (*ω*_L_ = 116 MHz). This can have two limiting cases:

• The frequency of the faster jump process is reduced below what can be observed considering the Larmor frequencies and the temperature range measured.

• The frequencies of both jump processes cannot be distinguished in the temperature and frequency range measured. This is possible if the frequencies approach each other. This is as also suggested by the data in ref. 25, where also intermediate substituent concentrations were measured.

(5) Due to the empirical parameter *β*, as well as the possibility of another jump process contributing to relaxation, it is not straightforward to interpret the results from the spectral density fitting of the Li_3−*x*_In_1−*x*_Zr_*x*_Cl_6_*x* = 0.3 and *x* = 0.5 materials. All that can be concluded is that the jump process is not BPP-type, and that only one process appears in the temperature range and at the frequencies measured. The line shapes show a decrease in chemical shift anisotropy from the pristine material to the substituted ones, which can be due to improved averaging of the environments by the motional process or the larger distribution of local environments due to the Zr-dopant.

Summarizing all these factors, from the NMR spin-lattice relaxometry it is clear that the introduction of Zr affects the Li-ion motion on fast timescales. Considering the two jump processes for Li_3_InCl_6_, which have diffusivities in the ratio of ∼1 : 10, there is clearly one dominant jump process for the diffusion. Considering the possible diffusion paths in the crystal structure, these are likely in the *ab*-plane and along the *z*-direction.

Anisotropic conduction seems to apply at longer timescales, as seen from the residual chemical shift anisotropy in the static line shapes (time scale of T_2_ relaxometry^41^) at high temperatures. This is present for both Li_3_InCl_6_ as well as, at a lesser degree, the Zr-substituted samples. The chemical shift anisotropy could be lower either due to the larger distribution in local environments due to the introduction of Zr, or because of more effective averaging in three dimensions.

From the structural analysis, the monoclinic symmetry with the layered arrangement of the In and Zr also suggests a different motional process along the *ab*-plane and the *c*-direction (the crystallographically unique axis). On introducing the Zr dopant, the In2-occupancy drops, in principle opening up the path along the *c*-direction allowing for a decrease in the anisotropy. The optimum in ionic conductivity is therefore likely a combination of optimized charge charrier concentration as well as improved conduction pathways along the *c*-direction due to the removal of In atoms from the conduction path.

## Conclusions

The substitution series Li_3−*x*_In_1−*x*_Zr _x_Cl_6_ (*x* = [0–0.5], steps of 0.1) was synthesised, resulting in a maximal ionic conductivity at *x* = 0.3 of 2.02 mS cm^−1^ at room temperature. Correlations between the structural models, derived from combined Rietveld refinement using X-ray and neutron diffraction data, and the hopping processes, probed by solid-state NMR, reveal a complex diffusion mechanism. The authors would like to highlight the importance of measuring both neutron and X-ray diffraction, for accurate determination of atomic occupancies in this system from powders, as well as measuring NMR-relaxometry data at multiple Larmor frequencies, due to the complex shape of the curve of temperature-dependent relaxation rates and the complexity of models, resulting in highly correlated fitting parameter sets.

For Li_3_InCl_6_, we find two distinct motional processes where one of the processes appears to dominate the long range diffusion and transport. From the layered structure, the diffusion path analysis and consideration of the most likely jump events, we assign the fast diffusion jump process to jumps within the Li layer in the *ab*-plane, and the slower jump process to jumps along the *c*-direction. Upon Zr substitution, the path along the *c*-direction seems to open up, due to a reduction in the In occupancy on the mixed In2/Li4 site. The second jump process probed for the pristine sample disappears for the substituted versions, and they most likely approach the timescale of the first, indicating reduced anisotropy. Nevertheless, the NMR spectral density fitting did not lead to an unambiguous choice of a spectral density applicable to all compositions. The residual chemical shift anisotropy in the static lineshapes of the substituted materials is less than that observed for the pristine material, though it is not completely averaged out. While the reduction could be due to a larger distribution in local environments due to the introduction of Zr, the fact that there is residual chemical shift anisotropy indicates that also for the substituted materials, the motion does not include all sites equally, even on longer timescales, and is hence anisotropic.

This paper shows that aliovalent doping does not only affect the charge carrier concentration, but can also lead to changes in the distribution of cations in the cell, affecting diffusivity on different timescales. For future studies, it would be interesting to combine this strategy with other successful strategies such as for example halogen alloying,^66^ which can affect ionic conductivity due to a change in lattice parameter (bottleneck size), lattice polarizability as well as potentially the configurational entropy.

## Materials and methods

### Synthesis

The materials were synthesized from the precursors LiCl, InCl_3_ and ZrCl_4_. All precursors were bought anhydrous from Sigma Aldrich and used as received. The materials were handled in an Ar-filled glovebox. Stoichiometric mixtures of the precursors were weighed and mixed in an agate mortar, then sealed under 200 mbar argon in quartz ampoules. The ampoules were annealed at 450 °C for 24 h, and then cooled down to room temperature over a 24 h duration. The resulting aggregates were then ball-milled for one hour (12 times 5 minutes with 2 minutes break) in a ZrO_2_ ball-mill jar at 450 RPM, with 3-mm ZrO_2_ balls, a ball to sample weight ratio of 1 : 25 and a batch size of 4 g. The ball-milled mixture was reannealed in evacuated quartz ampoules for 8 h at 450 °C. The reason for this synthesis route is that the powder obtained from the first annealing step resulted in X-ray diffraction patterns with varying relative intensities, which were assumed to be due to preferential orientation. Therefore, the powder was ball-milled, to reduce the crystallite size. The ball-milled powder, however, had an order of magnitude lower ionic conductivity, and showed very broad peaks in the X-ray diffraction pattern. Therefore, the fine powder was annealed again, which gave the desired combination of high ionic conductivity, but an X-ray diffraction pattern of sufficient quality for Rietveld structure refinement. Recent research suggests that materials of the here investigated Li_3_MX_6_ structure families exhibit varying degrees of stacking faults.^52,53^ It can't be excluded that the reason for the varying intensities here are due to a different peak shape due to possible stacking faults, but in the final synthesis product stacking faults were not needed for the final Rietveld refinement.

### ICP-OES

Approximately 30 mg of each sample was dissolved in 4.5 ml 30% HCl + 1.5 ml 65% HNO_3_ (+0.2 ml 40% HF) using a microwave oven. The dissolution time in the microwave oven was 60 min. After destruction, the samples were diluted to 50 ml with Milli-Q water. The samples were also diluted by a factor of 20 for In, Zr, Li. The samples were analysed with ICP-OES 5300DV.

### X-ray diffraction

The X-ray diffraction patterns were collected on a Panalytical X'Pert Pro X-ray diffractometer with a Cu K-alpha source in a 2*θ* range of 10 to 100° in reflection geometry. Due to the moisture sensitivity of the samples, a custom-made airtight sample holder was used. The sample holder consisted of a zero-diffraction silicon wafer (SilTronix), closed off with a kapton half-cylinder. The instrument parameter file was created by measuring a LaB_6_ standard obtained from NIST.

### Neutron diffraction

Neutron diffraction measurements were performed at room temperature on the time of flight (TOF), high-flux, medium-resolution diffractometer Polaris at ISIS,^54^ Rutherford Appleton Laboratory, United Kingdom. 1g each of the Li_3−*x*_In_1−*x*_Zr_*x*_Cl_6_ (*x* = 0,0.2,0.3,0.4,0.5) samples were filled in cylindrical vanadium cans under argon atmosphere and sealed with indium wire. Normalized diffraction data collected in the highest resolution backscattering detector bank (bank 5, *Q*-range ∼2.25 to 16 Å were used in Rietveld structure refinement. Note the sample with *x* = 0.1 was not measured due to limitations in beamtime.

### Rietveld refinement

Combined Rietveld refinement using the X-ray and neutron diffraction data was performed with the software GSAS-II.^55^ The relative amounts of the elements In, Zr and Li was fixed to values obtained by ICP. The structure published by Bohnsack *et al.*^56^ was used as the starting structure. Both Li and Zr were initially put on the two partially occupied In sites, but Li clearly preferred a specific site (In2), and Zr the other indium (In1) site, hence the reversed positions were removed. We highlight the importance of using two contrasts here, as the co-occupancy of the In2 site with Li makes the refinement of the occupancies difficult from neutron diffraction data alone, due to the opposite sign scattering length density. Observed scattering-density maps of both the neutron and X-ray data were also calculated using GSAS-II.^55,57^ From the density maps calculated of the neutron diffraction data, an additional tetrahedral site was added for the materials Li_3_InCl_6_ and Li_2.8_In_0.8_Zr_0.2_Cl_6_ (Li3). Vesta^58^ was used for visualizations of the crystal structure and density maps.

### AC-impedance

The ionic conductivity of the materials was evaluated on dense cylindrical pellets. To make the pellets, 0.2 g of solid electrolyte powder was loaded in a home-made cell consisting of stainless steel plungers in an insulating Al_2_O_3_ ring of 1 cm diameter. The powder was compressed with uniaxial load of 1.6 tons, screwed tight and sealed with blu tack. The resulting pellets were 0.1 cm ± 5% thick. The impedance measurements were conducted on an Autolab FRA32M with the ECI10M high-frequency module in a frequency range from 1 Hz to 10 MHz using an excitation amplitude of 0.1 V. The individual AC-impedance spectra were fit using RelaxIS (see ESI files† for a full report with the data, fits to the data and fitted parameter with error). The validity of the equivalent circuit model was confirmed using the Kramers Krönig analysis also implemented in RelaxIS (ESI Fig. X†). The fitted values for the pellet resistance was used to calculate the ionic conductivity and Arrhenius relationship. For the ionic conductivity, the errors were calculated using Gaussian error propagation,^43^ for the activation energies from the fit to the Arrhenius behaviour (ESI Tables†).

AC-impedance measurements are the common standard to evaluate the ionic conductivity of solid electrolytes, as the time and length scales correspond the most with the relevant scales for battery operation. Unfortunately, research has shown that values of the ionic conductivity and activation energy can vary strongly for the same materials in different labs.^59^ Solid-state NMR can be used as a complementary probe for the motion the Li-ions on a much shorter timescale.

### Solid-state NMR

Solid-state NMR measurements were performed on an Agilent 400 MHz spectrometer (*B*_0_ = 9.4 T, 155.5 MHz for ^7^Li) and an Agilent 300 MHz spectrometer (*B*_0_ = 7.1 T, 116.6 MHz for ^7^Li and 44 MHz for ^6^Li). Chemical shifts were referenced with respect to a 0.1 M LiCl solution. Variable temperature measurements were performed using a 5 mm static goniometer probe. T_1_ relaxation times were determined at various temperatures using a saturation recovery experiment. The T_1_s as well as the static line-shapes were fit using the ssNAKE program.^60^ For a detailed explanation of the theory behind the spectral density fitting as well as the CSA, see ESI Text 1.†

## Conflicts of interest

The authors declare no competing interests.

## Supplementary Material

TA-011-D2TA08433C-s001

TA-011-D2TA08433C-s002

TA-011-D2TA08433C-s003
