# Supplementary material for: Re-investigating the structure–property relationship of the solid electrolytes Li 3−xIn1−xZrxCl6 and the impact of In–Zr(iv) substitution
Source: J Mater Chem A Mater. 2023 Jan 25;11(9):4559–71. doi: 10.1039/d2ta08433c (PMC9969333; doi:10.1039/d2ta08433c)
Supplement: TA-011-D2TA08433C-s001 [file TA-011-D2TA08433C-s001.pdf]

Electronic Supplementary Material (ESI) for Journal of Materials Chemistry A.
This journal is © The Royal Society of Chemistry 2023

Supplementary Information - Re-investigating the structure-property relationship of the solid electrolyte $Li_{3-x}In_{1-x}Zr_xCl_6$ and the impact of In-Zr(IV) substitution

Eveline van der Maas[1], Theodosios Famprikis,[1] Saskia Pieters[2], Jonas P. Dijkstra[2], Zhaolong Li[1], Steven R. Parnell[1], Ron Smith[3], Ernst R.H. van Eck[2], Swapna Ganapathy[1*], Marnix Wagemaker[1*]*

[1] Department of Radiation Science and Technology, Delft University of Technology, Delft, the Netherlands.

[2]Radboud university, Nijmegen, Netherlands

[3]ISIS facility, Rutherford Appleton Laboratory, Chilton, Didcot, Oxfordshire, United Kingdom, OX11 0QX

* Corresponding email: erhve@nmr.ru.nl, s.ganapathy@tudelft.nl, m.wagemaker@tudelft.nl

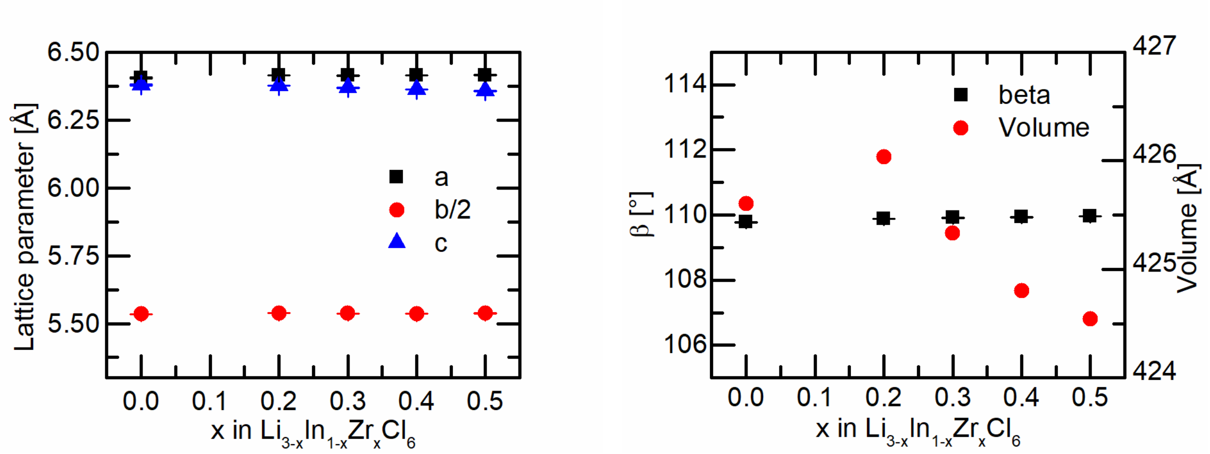

Figure S1: Lattice parameters (left) and unit cell angle and volume (right) of the Rietveld refinement of the neutron and the x-ray data. The error bars of the volume are smaller than the dots.

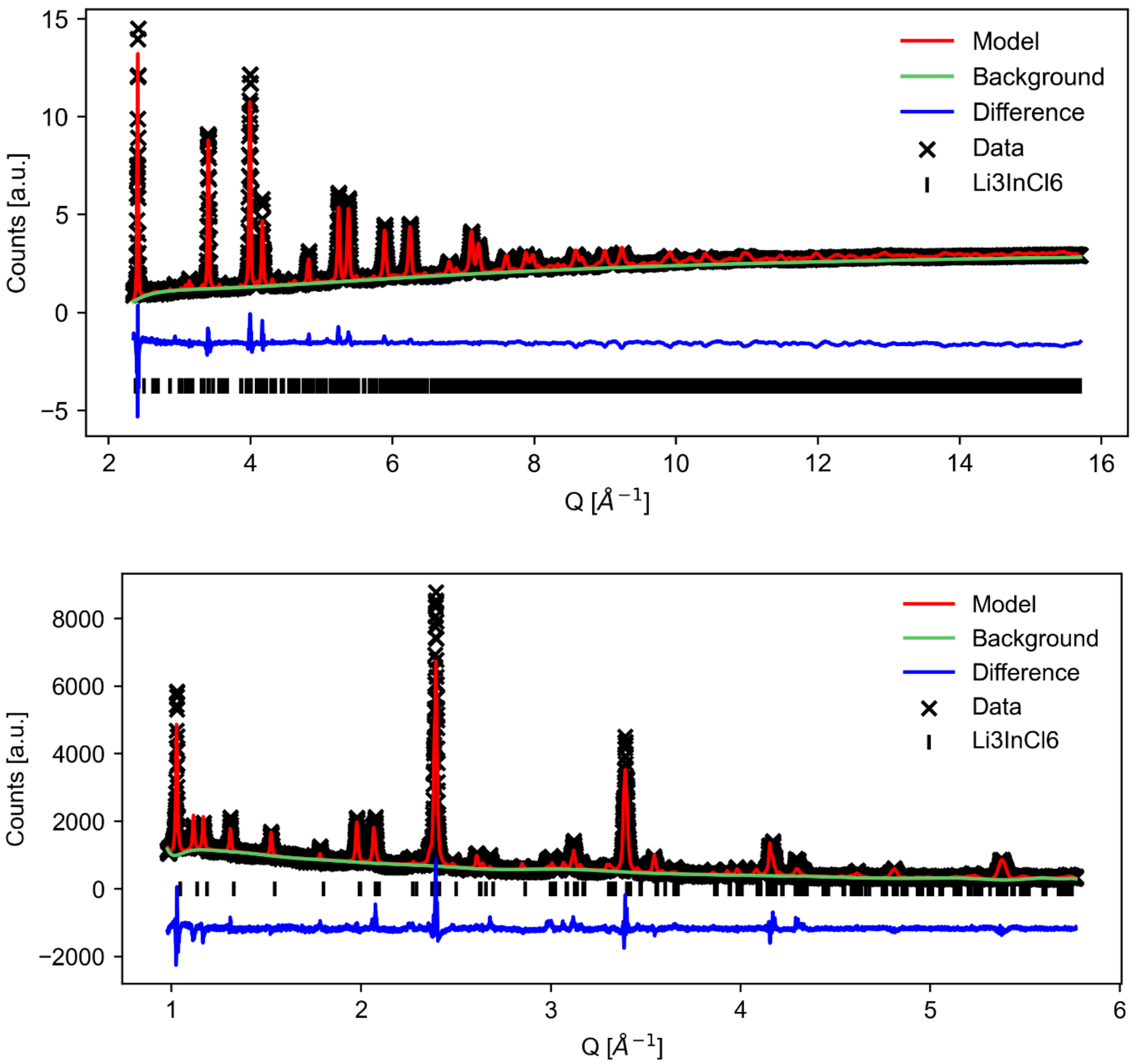

Figure S2: Combined Rietveld fit to the neutron diffraction data (top, bank 5) and x-ray diffraction data (bottom) of $Li_{3-x}In_{1-x}Zr_xCl_6$ with x=0.

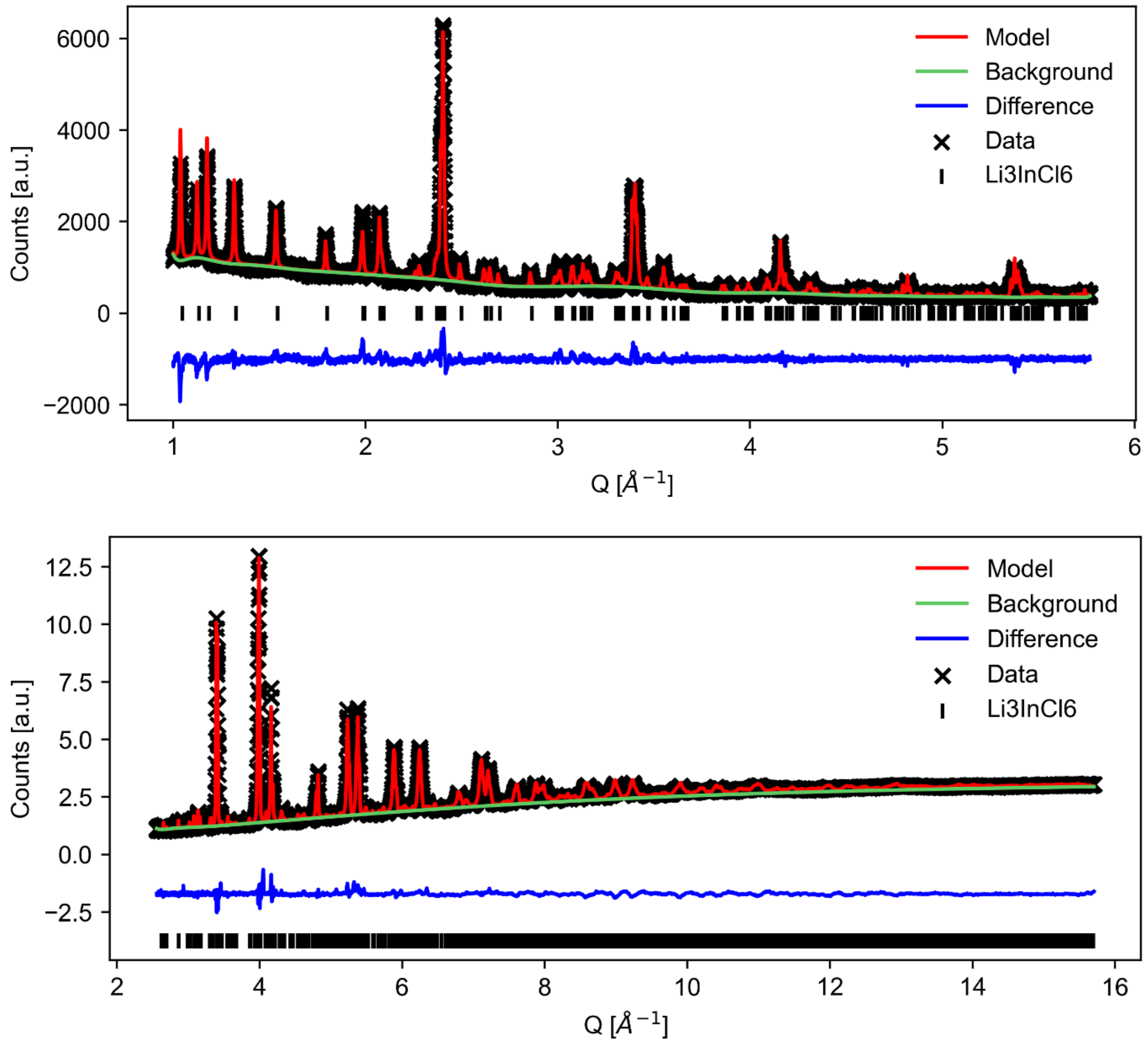

Figure S3: Combined Rietveld fit to the neutron diffraction data (top, bank 5) and x-ray diffraction data (bottom) of $Li_{3-x}In_{1-x}Zr_xCl_6$ with x=0.2.

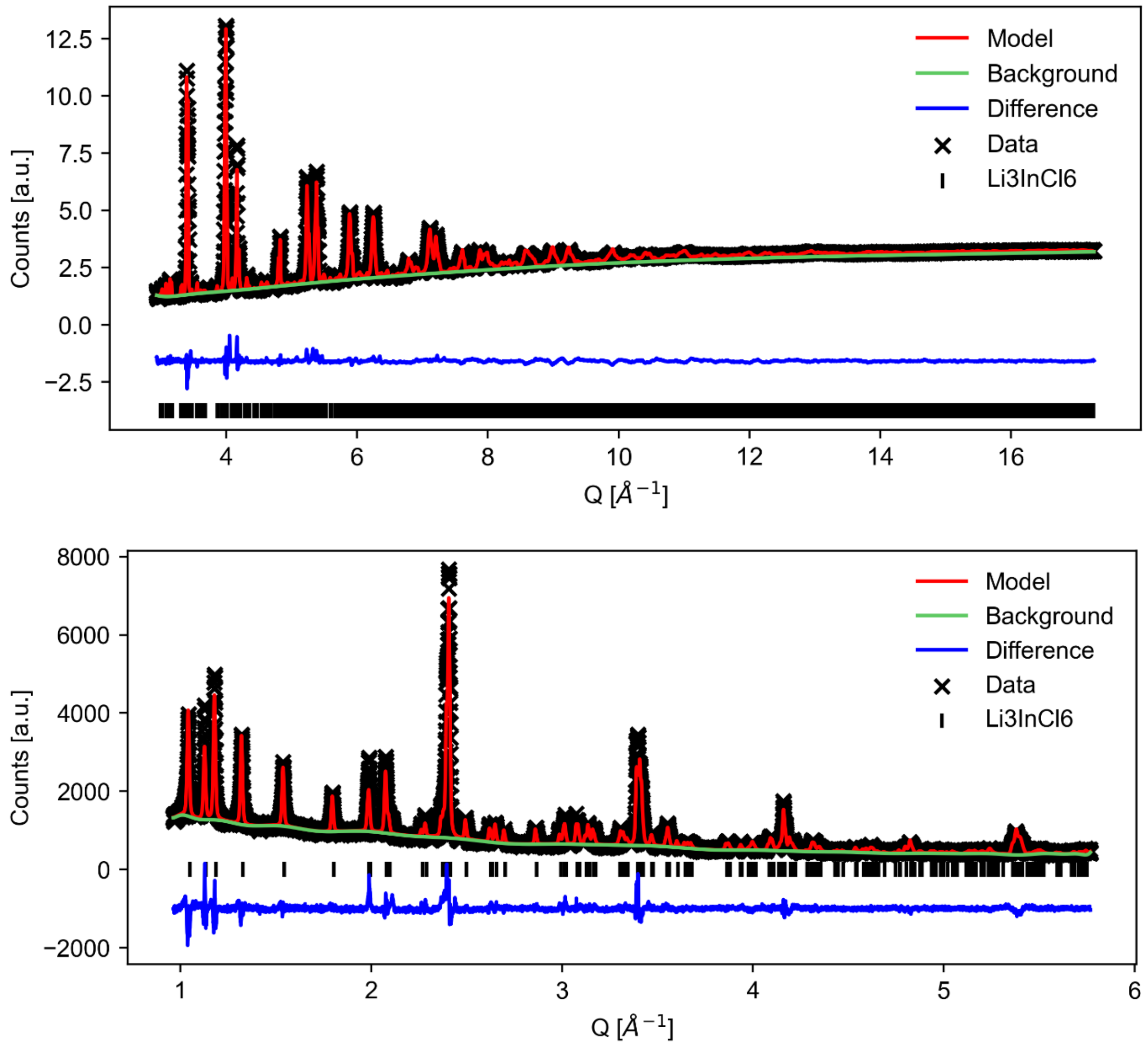

Figure S4: Combined Rietveld fit to the neutron diffraction data (top, bank 5) and x-ray diffraction data (bottom) of $Li_{3-x}In_{1-x}Zr_xCl_6$ with x=0.3.

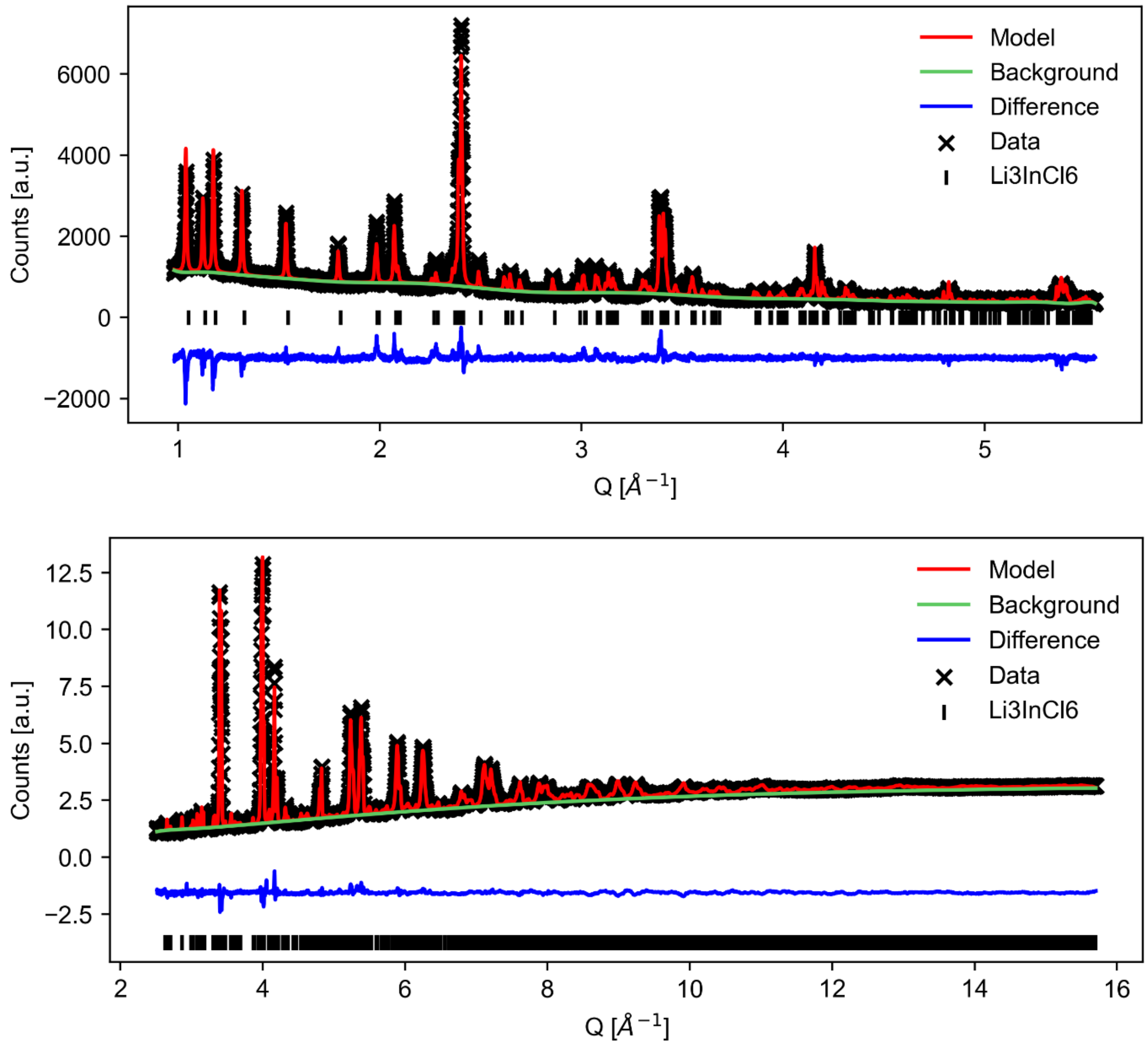

Figure S5: Combined Rietveld fit to the neutron diffraction data (top, bank 5) and x-ray diffraction data (bottom) of $Li_{3-x}In_{1-x}Zr_xCl_6$ with x=0.4.

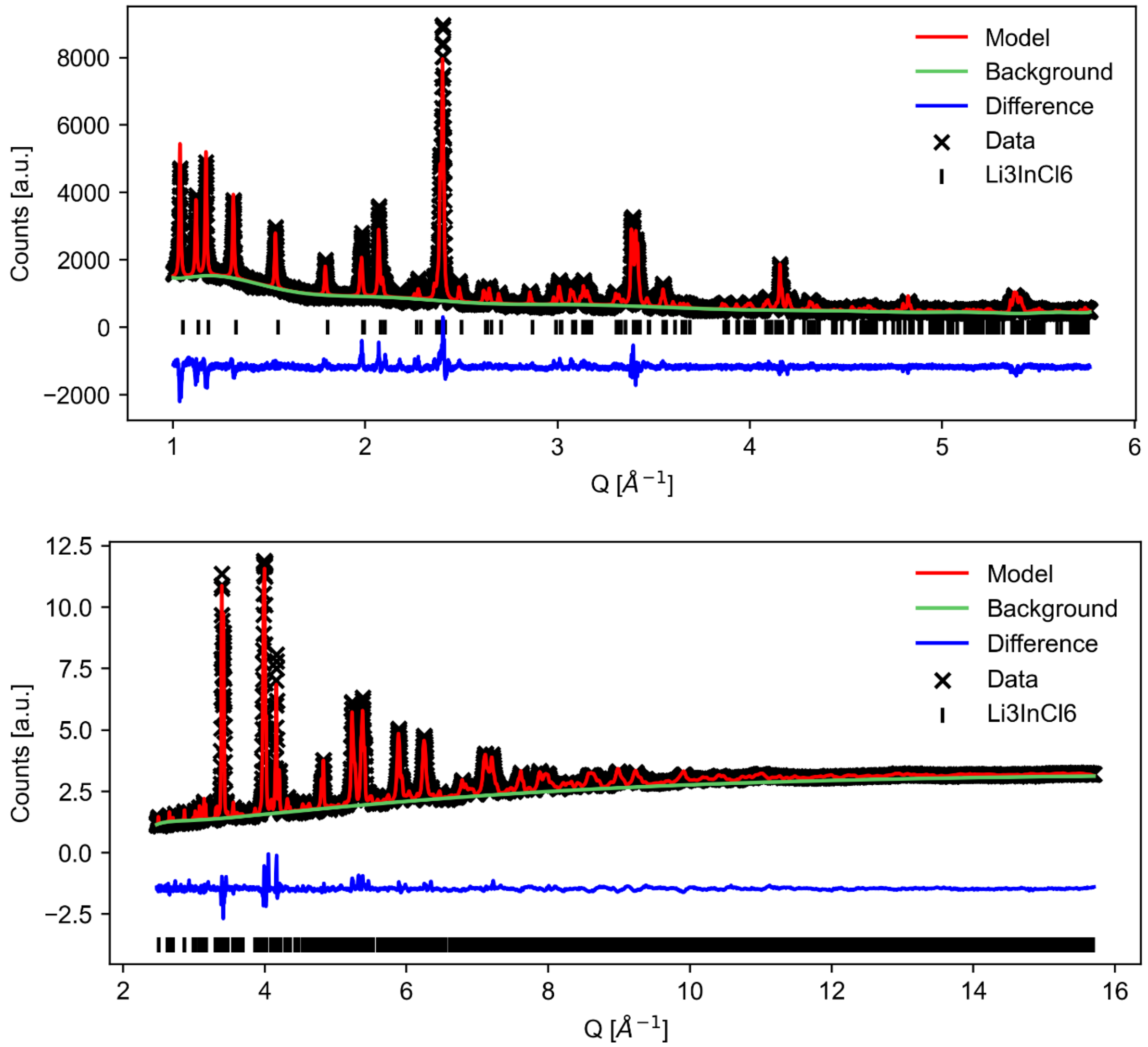

Figure S6: Combined Rietveld fit to the neutron diffraction data (top, bank 5) and x-ray diffraction data (bottom) of $Li_{3-x}In_{1-x}Zr_xCl_6$ with x=0.5.

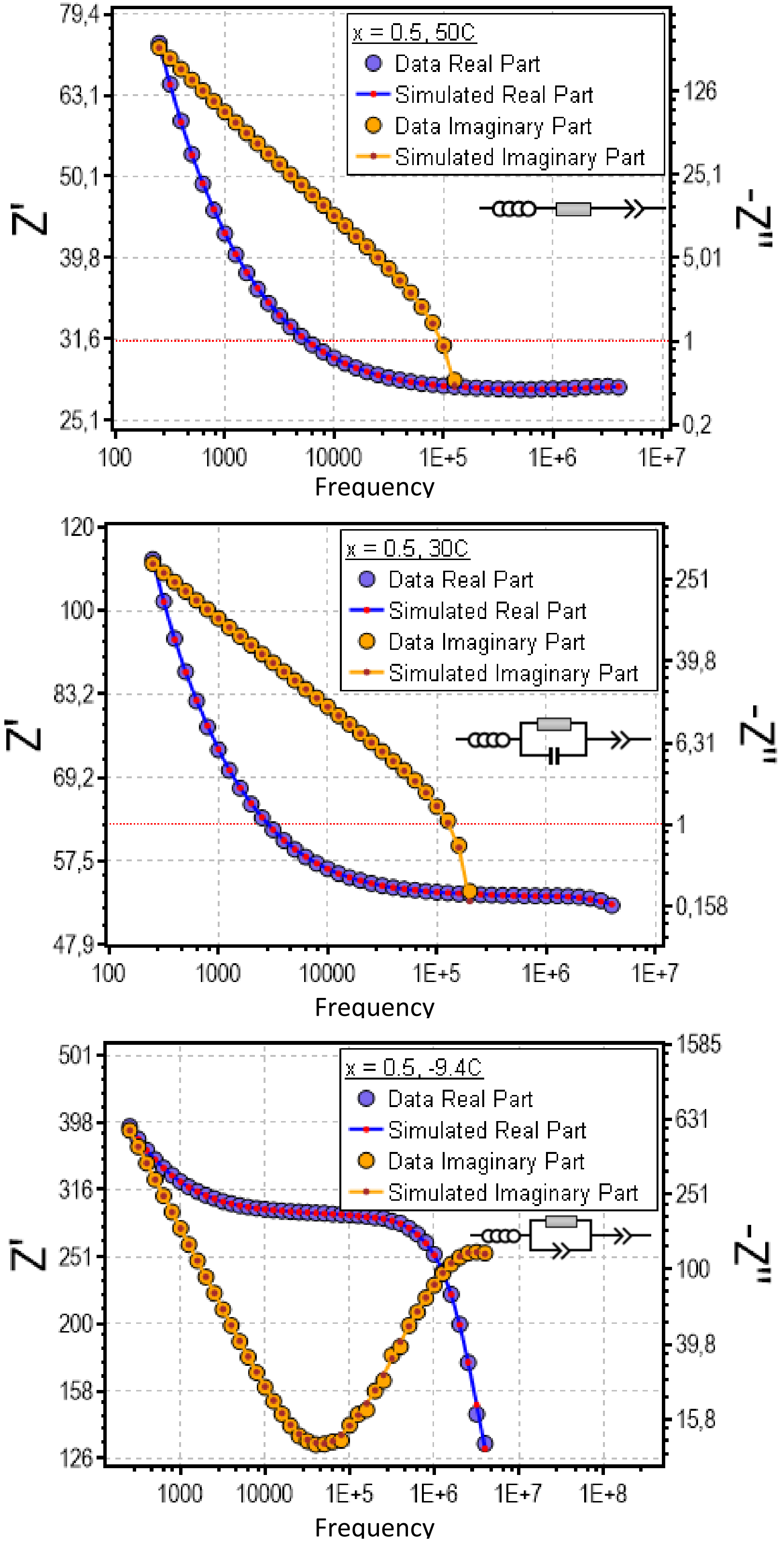

Figure S7: Confirmation of the validity of the chosen equivalent circuits to fit the AC-impedance spectra by the Kramers-Krönig analysis. The examples chosen here are from the material $Li_{3-x}In_{1-x}Zr_xCl_6$ with x=0.5 at 50 (high temperature L-R-CPE), 30 (medium temperature L-(R)(P)-CPE) and low temperature (L-(R)(CPE)-(CPE) at -9.4 °C.

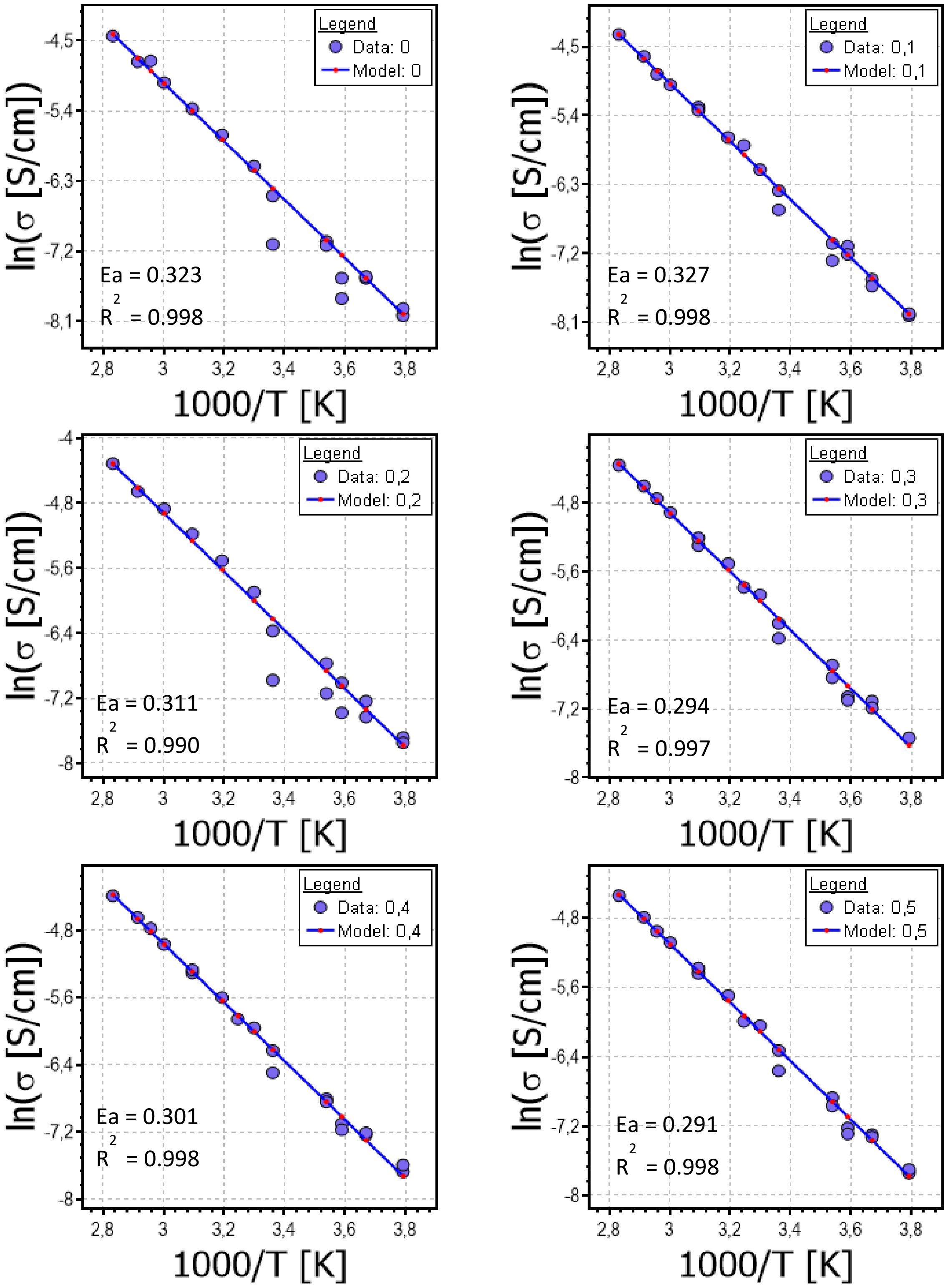

Figure S8: Arrhenius Relationship extracted from the AC-impedance measurements of the materials $Li_{3-x}In_{1-x}Zr_xCl_6$ for x=0-0.5.

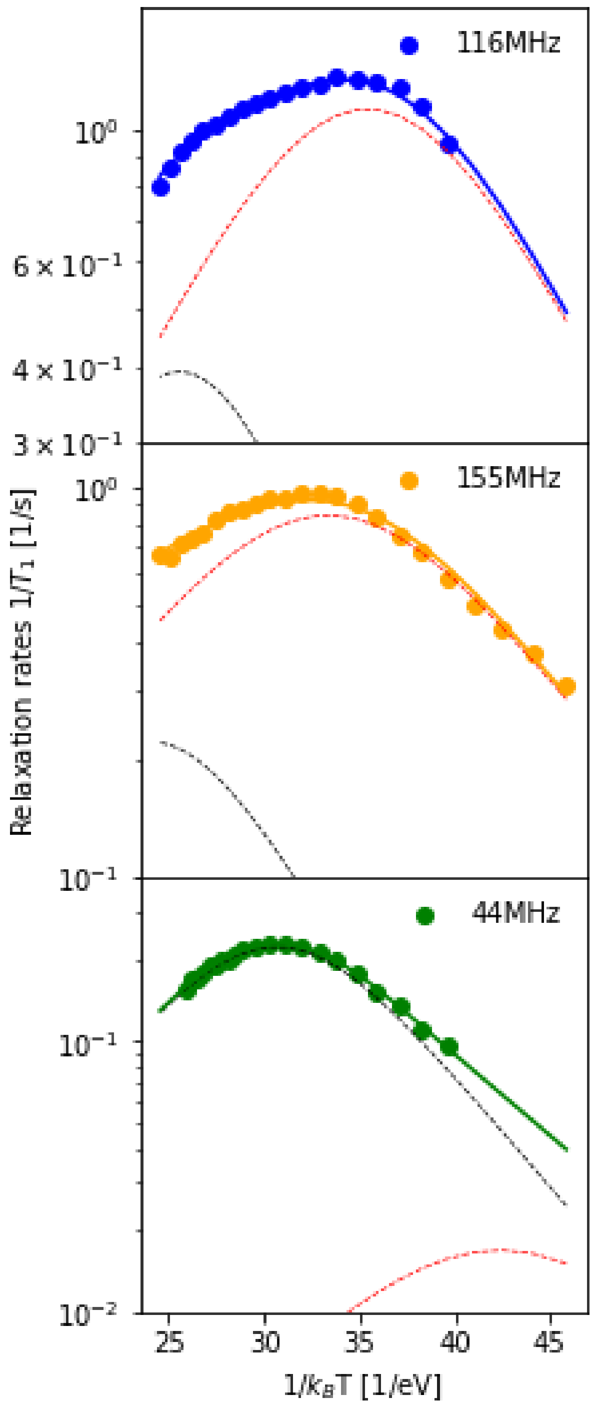

Figure S9: Relaxation rates of Li in Li_3InCl_6 measured at three different larmor frequencies plotted on logarithmic scale. 116MHz corresponds to ^{7}Li measured on a 300MHz, 155MHz to ^{7}Li on a 400MHz and 44MHz to ^{6}Li on a 300 MHz spectrometer. The contribution of the individual jump processes to the relaxation rate are shown in red and black (same processes in all plots) and illustrate which process the datasets are sensitive to.

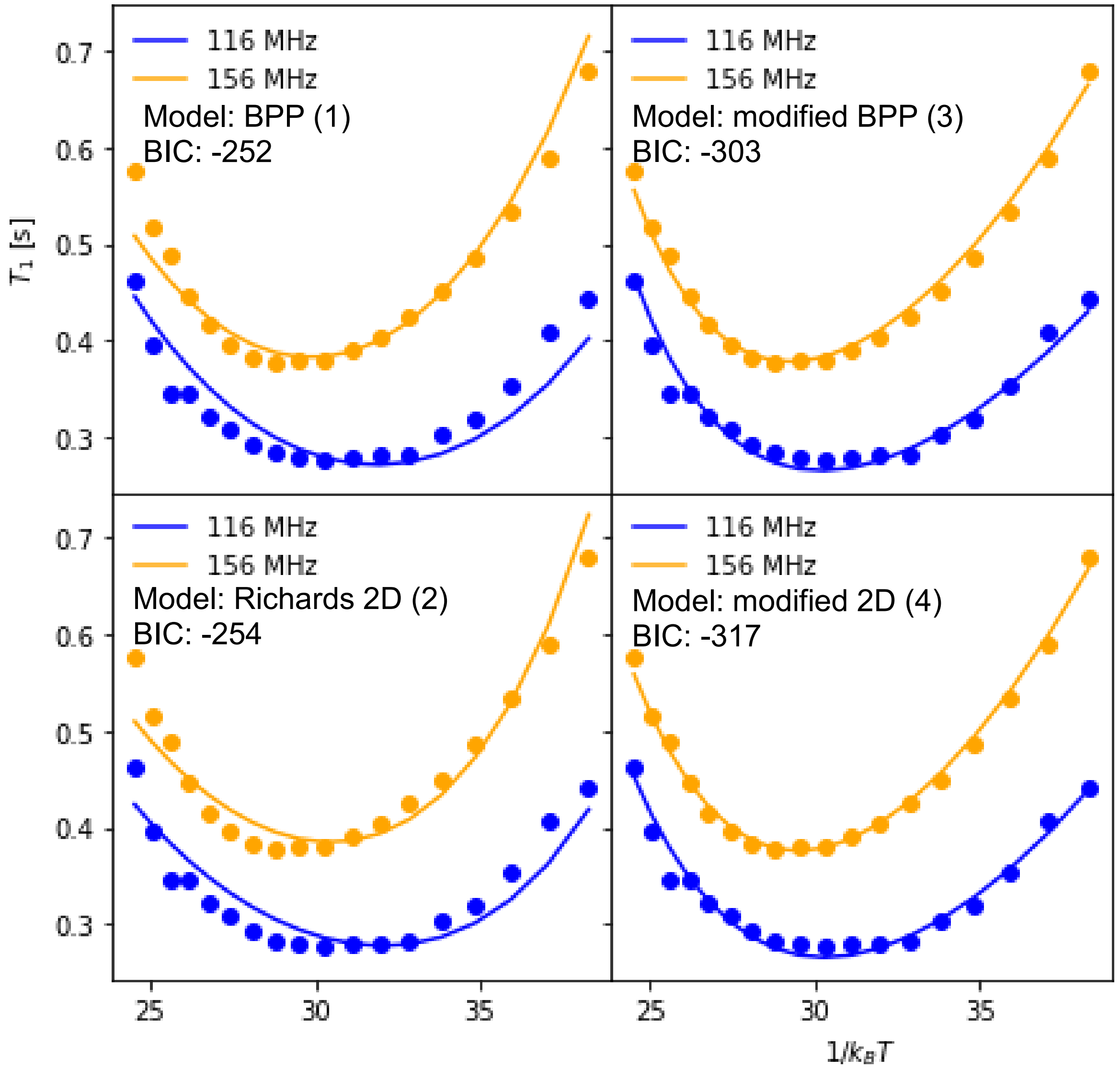

Figure S10: The relaxation data of $Li_{3-x}In_{1-x}Zr_xCl_6$ at x=0 and fits of the different models (1-4) as described in the main text (BPP model with one jump process only). The fits are visualized with the T_1 in the y-axis, as the differences in the models can be seen more clearly. The plot shows that both empirically modified datasets fit the data well, with a small preference for the modified 2D model (4) as indicated by the smaller BIC.

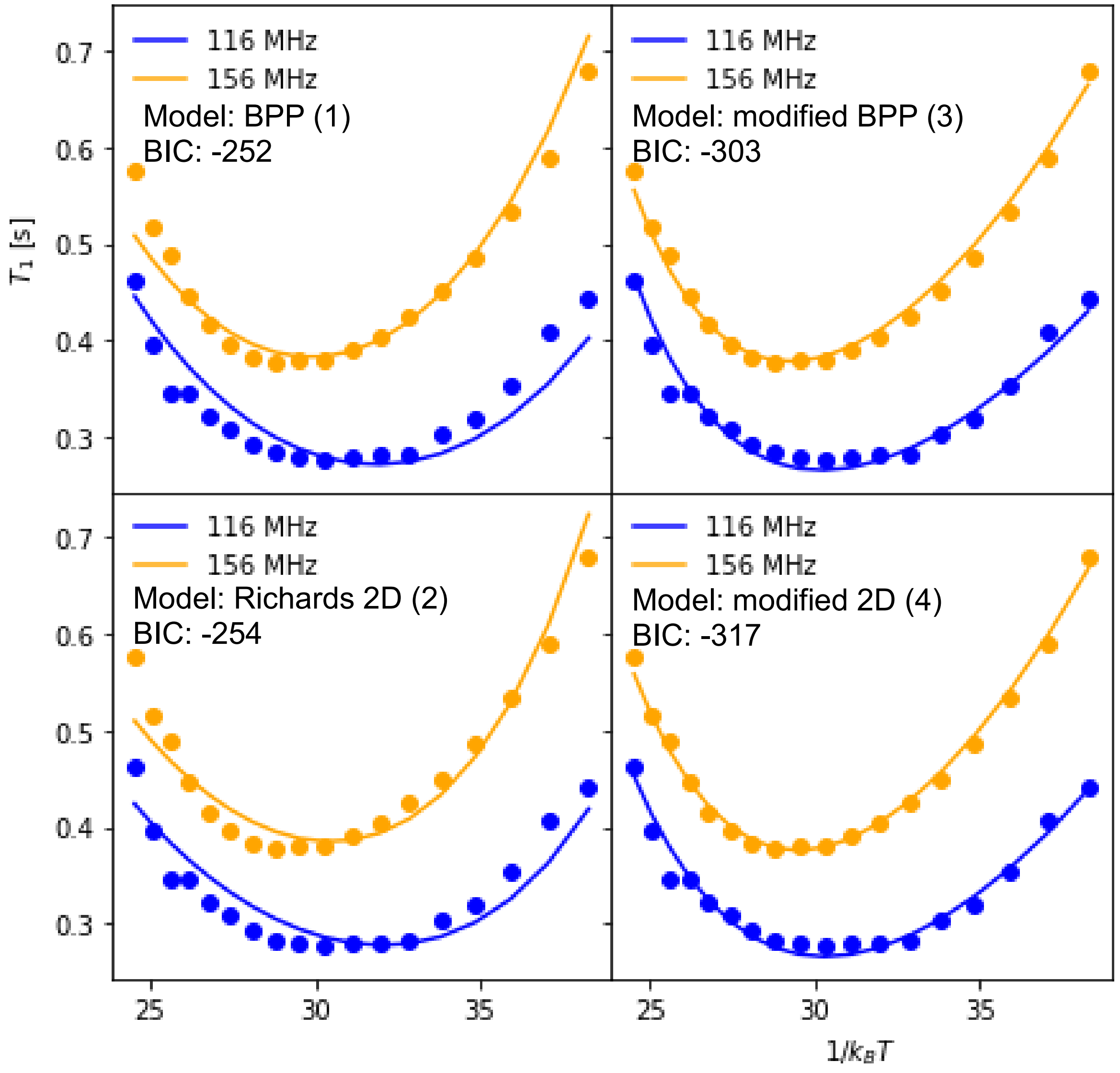

Figure S11: The relaxation data of $Li_{3-x}In_{1-x}Zr_xCl_6$ x=0.3 and fits of the different models (1-4) as described in the main text. The fits are visualized with the T_1 in the y-axis, as the differences in the models can be seen more clearly. The plot shows that both empirically modified datasets fit the data well, with a small preference for the modified 2D model (4) as indicated by the smaller BIC.

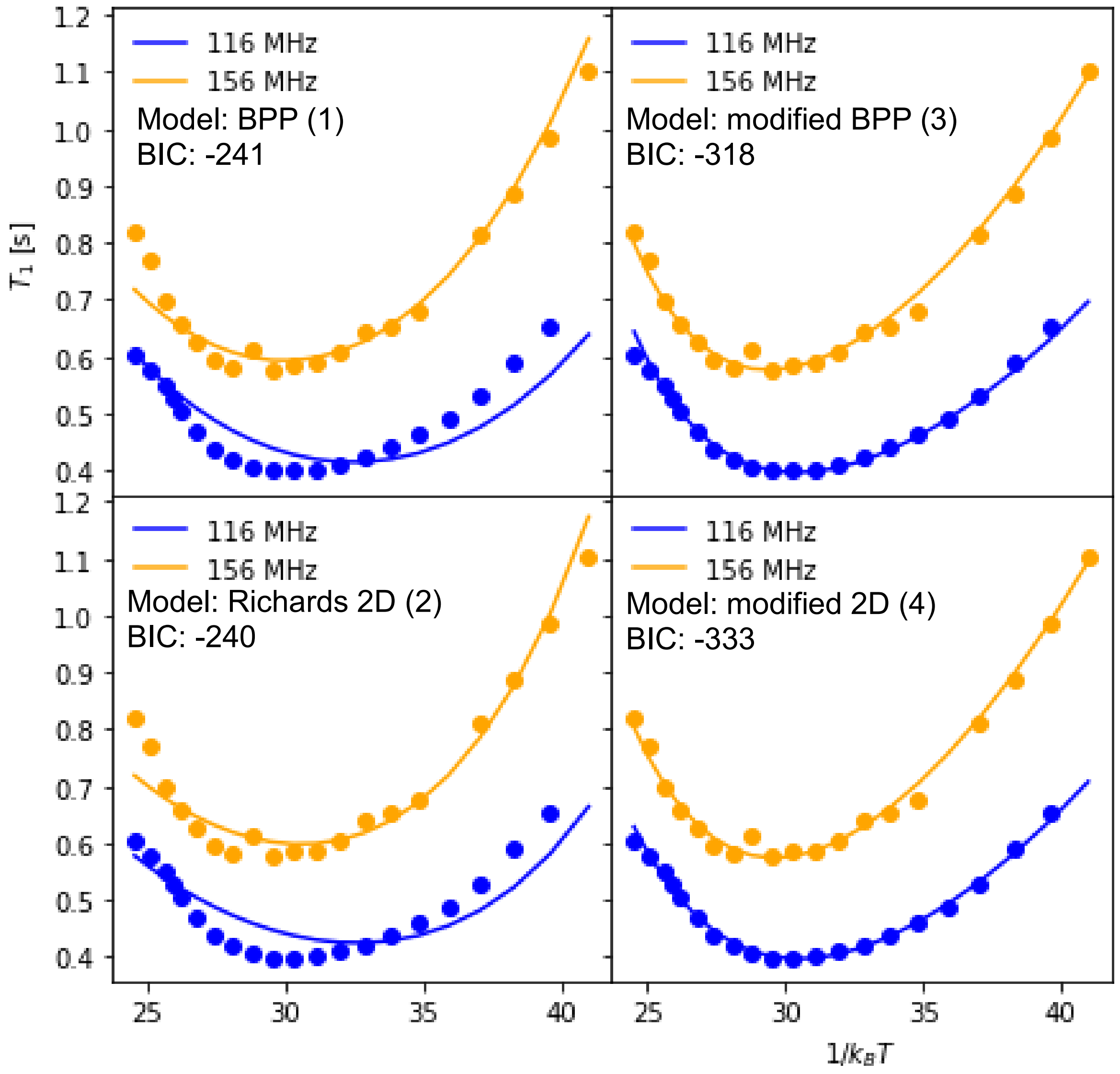

Figure S12: The relaxation data of $Li_{3-x}In_{1-x}Zr_xCl_6$ x=0.5 and fits of the different models (1-4) as described in the main text. The fits are visualized with the T_1 in the y-axis, as the differences in the models can be seen more clearly. The plot shows that both empirically modified datasets fit the data well, with a small preference for the modified 2D model (4) as indicated by the smaller BIC.

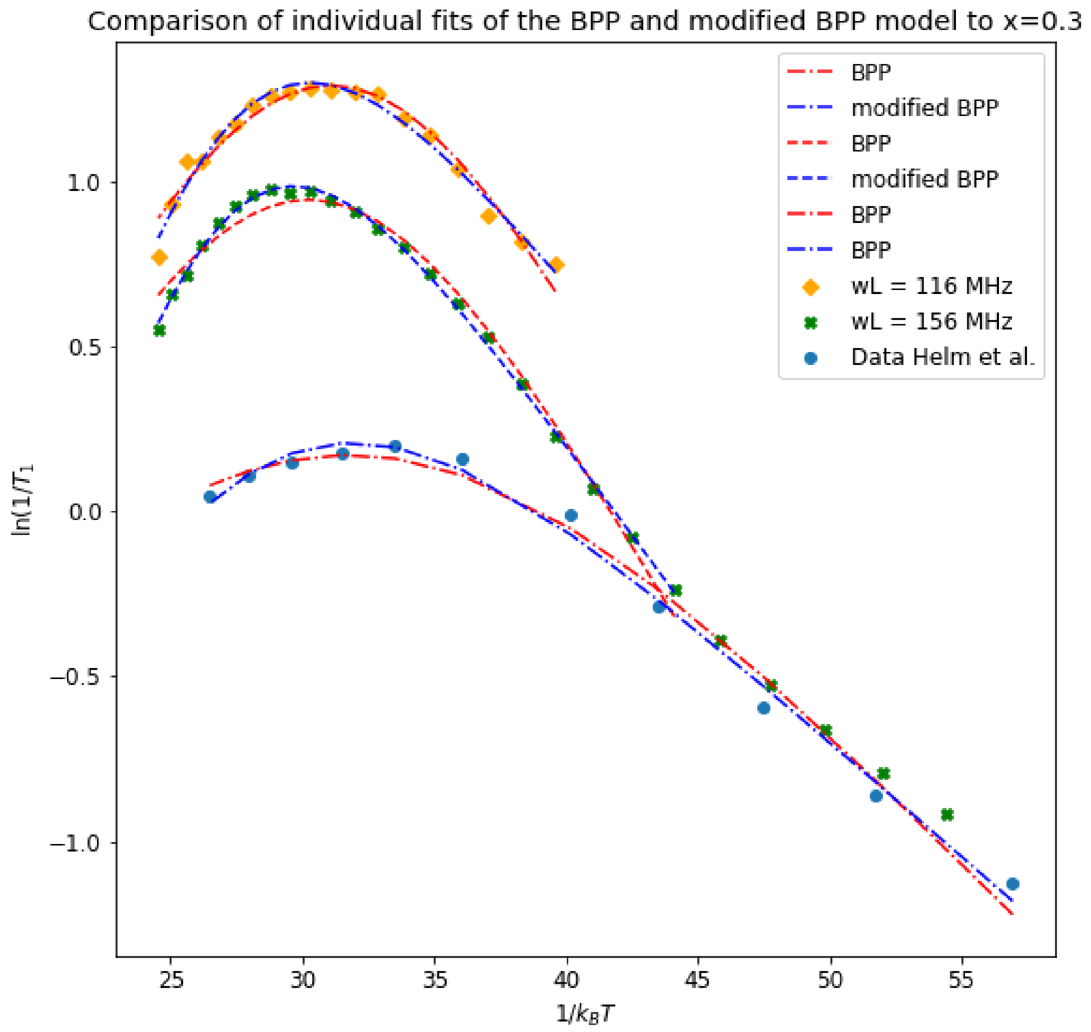

Figure S13: Comparison of the data for $Li_{3-x}In_{1-x}Zr_xCl_6$ (x=0.3). The data from Helm et al.[1] represents approximate values obtained using plot digitize

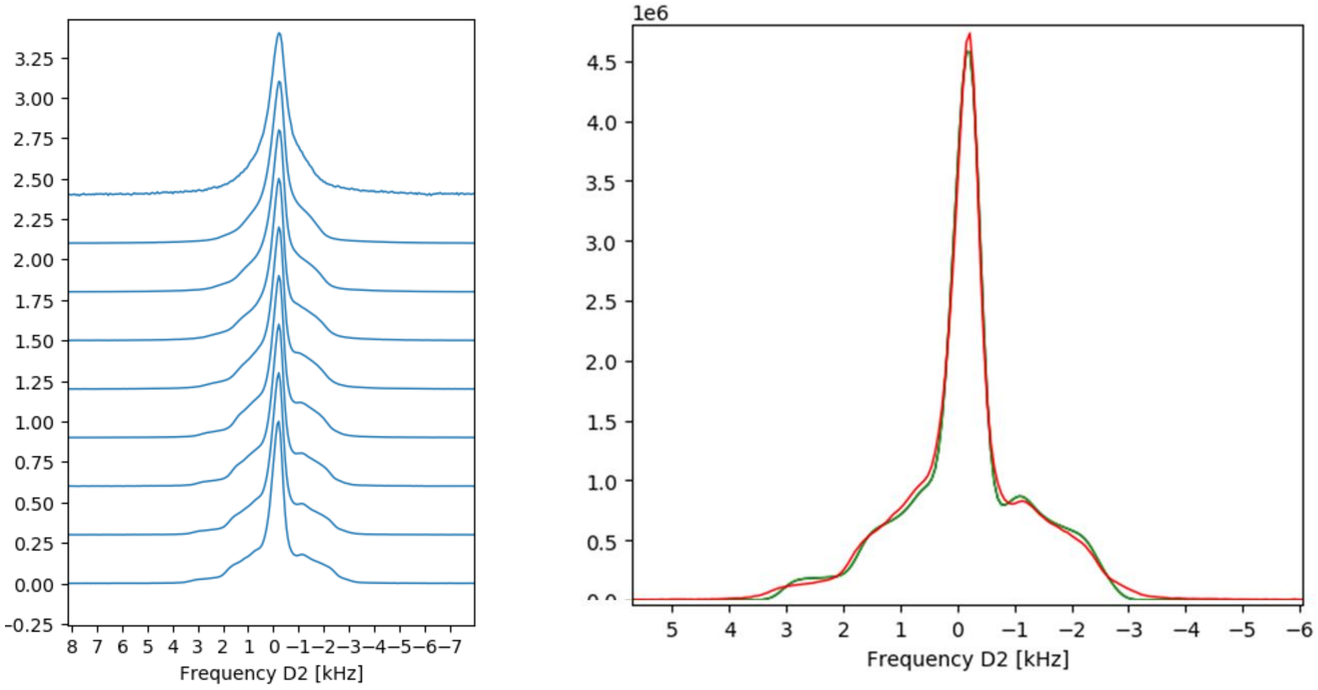

Figure S14: Left: Variable temperature ^{7}Li static NMR spectra of Li_3InCl_6 at 9.4T (156 MHz) at T = 233 K (top) and T = 493K (bottom). Already at 233K the line is motionally narrowed but at 493K the residual broadening is so small that sharp features emerge. Right: The high temperature spectrum at 493K can only be approximated by taking into account a residual quadrupolar coupling of about 6kHz as well as CSA with a span of 3 ppm and a skew of -0.76.

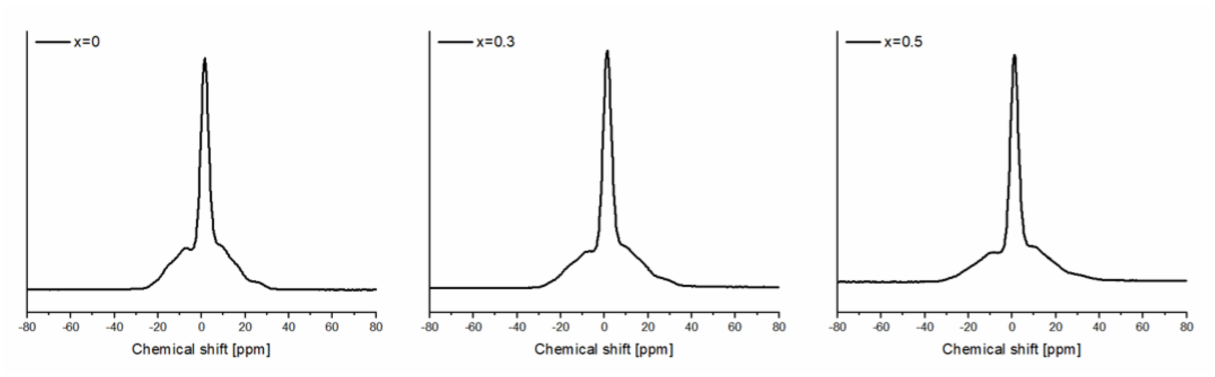

Figure S15: ^{7}Li static NMR spectra of $Li_{3-x}In_{1-x}Zr_xCl_6$, at 9.4T (156 MHz) at T = 493K. The central transition is motionally narrowed in all three samples. The shape of the satellites can only be approximated by a combination of quadrupolar coupling as well as chemical shift anisotropy, see Figure S14. The shape is most pronounced in Li_3InCl_6, and slightly flattens for the doped samples. The residual chemical shift anisotropy that resolves at the satellites indicates that the motion of the Li-ions across the different sites does not average out completely.

AN2020.12.002	Weighted amount [g]	Li[wt%]	In[wt%]	Zr[wt%]	destr.
Li3In(1-x)ZrxCl6 x=0	0,0295	4,8	27,0	5,9	AR+HF
Li3In(1-x)ZrxCl6 x=0.1	0,0249	5,7	29,6	2,6	AR+HF
Li3In(1-x)ZrxCl6 x=0.2	0,0257	5,5	25,8	4,8	AR+HF
Li3In(1-x)ZrxCl6 x=0.3	0,0282	5,3	22,7	7,2	AR+HF
Li3In(1-x)ZrxCl6 x=0.4	0,0301	5,2	20,3	9,8	AR+HF
Li3In(1-x)ZrxCl6 x=0.5	0,0298	5,1	17,0	12,6	AR+HF
ZrCl4	0,0207			37,2	AR+HF
InCl3	0,0248		48,2		AR+HF
Li3In(1-x)ZrxCl6 x=0	0,0288	5,6	31,7	0,0	AR
Li3In(1-x)ZrxCl6 x=0.1	0,0292	5,1	26,7	2,1	AR
Li3In(1-x)ZrxCl6 x=0.2	0,0290	5,6	26,1	4,6	AR
Li3In(1-x)ZrxCl6 x=0.3	0,0273	5,4	23,2	7,2	AR
Li3In(1-x)ZrxCl6 x=0.4	0,0206	5,2	20,6	9,5	AR
Li3In(1-x)ZrxCl6 x=0.5	0,0265	5,1	17,3	12,5	AR
ZrCl4	0,0280			37,7	AR
LiCl	0,0369	15,5			AR+HF

Table S1: Elemental compositions resulting from ICP (inductively coupled plasma) spectrometry. AR stands for Aqua Regia (HNO_3 + 3 HCl). The Zr dropped out as ZrF_4 when dissolved with AR+HF, which is why the measurements with AR were used

x	R [Ohm]	dR [Ohm]	thickness [cm]	dthickness [cm]	A [cm2]	sigma [S/cm]	dsigma/sigma [%]	dsigma [S/cm]
0,5	71,9	0,143	0,1	0,005	0,785	1,77E-03	5,004%	8,86E-05
0,4	64,8	0,184	0,1	0,005	0,785	1,96E-03	5,008%	9,84E-05
0,3	62,88	0,078	0,1	0,005	0,785	2,02E-03	5,002%	1,01E-04
0,2	74,29	0,056	0,1	0,005	0,785	1,71E-03	5,001%	8,57E-05
0,1	75,1	0,074	0,1	0,005	0,785	1,70E-03	5,001%	8,48E-05
0	83,72	0,178	0,1	0,005	0,785	1,52E-03	5,005%	7,61E-05

Table S2: Error propagation according to formula

$$\frac{\Delta\sigma}{\sigma} = \sqrt{\left(\frac{\Delta d}{d}\right)^2 + \left(\frac{\Delta R}{R}\right)^2 + 2\pi \times \left(\frac{\Delta r}{r}\right)^2}$$

Were d is the pellet thickness, R the parameter fit of the pellet resistance and r the radius of the pellet.The deviation of the radius was assumed to be zero and the errors of the parameter fits are small, rendering the error in the thickness to dominate the total error.

x	ΔE_Activation,rel / %	E_Activation [eV]	dEa [eV]
0.5	3.32	0.28271	0.00939
0.4	2.39	0.28964	0.00693
0.3	3.83	0.277	0.0106
0.2	2.67	0.29745	0.00796
0.1	2.20	0.33806	0.00742
0.0	6.36	0.30638	0.01949

Table S3: Error of the activation energies, obtained from the fits of the temperature dependent ionic conductivity, see Figure S8.

**$Li_{3-x}In_{1-x}Zr_xCl_6$**	**τ_0 [s]**	**E_a [eV]**	**β**	**$E_{a,slope}$ [eV]**	**Reference**
x=0	1.5e-11	0.189, 0.131	- BPP	0.106, 0.0212	This work
x=0.3	(3.3 ± 1.7) x10^{-13}	0.30±0.02	0.29±0.04	0.142	This work
x=0.5	(6.6 ± 2.5) x10^{-13}	0.28±0.02	0.26±0.02	0.12	This work
x=0	Not provided	0.291	0.22	I.d.	Helm et al[1]
x=0.1	Not provided	0.203	0.44	I.d.	Helm et al[1]
x=0.2	Not provided	0.143	0.63	I.d.	Helm et al[1]
x=0.3	Not provided	0.117	0.65	I.d.	Helm et al[1]
x=0.4	Not provided	0.116	0.66	I.d.	Helm et al[1]
x=0.5	Not provided	0.136	0.57	I.d.	Helm et al[1]

Table S4: Fitting parameters of the spectral densities used to fit the relaxation data (τ_0, E_a, β). The standard errors reported here are those that raise chi-square by 1. For comparison, activation energies extracted from the high temperature slope of the data ($E_{a,s}$), I.d. stands for insufficient datapoints (a minimum of 5 points were used to fit the line). Values from literature included for comparison (see column Reference).

$Li_{3-x}In_{1-x}Zr_xCl_6$	**τ_0 [s]**	**E_a [eV]**	**β**	**Corr τ_0 - E_a**	**Corr τ_0 - β**	**Corr E_a- β**	**Reference**
x=0.3 300MHz	$1.46 \pm 0.2 x 10^{-11}$	0.146±0.005	BPP	-0.993	-	-	This work
x=0.3 300MHz	$1.18 \pm 0.8 x 10^{-12}$	0.257±0.003	0.365	-0.997	0.962	-0.978	This work
x=0.3 400MHz	$1.49 \pm 0.1 x\ 10^{-11}$	0.140±0.0003	BPP	-0.991	-	-	This work
x=0.3 400MHz	$9.12 \pm 0.2 x 10^{-13}$	0.256±0.001	0.427	-0.998	0.971	-0.984	This work
x=0.3 300MHz	$1.00 \pm 0.2 x\ 10^{-10}$	0.082±0004	BPP	-0.98	-	-	Fit in this work, data digitized from Helm et al[1]
x=0.3 300MHz	$1.46 \pm x\ 10^{-11}$	0.16±0.004	0.437	-0.996	0.978	-0.991	Fit in this work, data digitized from Helm et al[1]
X =0.3 300MHz	-	0.117	0.65	-	-	-	Helm et al[1]

Table S5: Parameters of individual fits as shown in Figure S12 and their correlations.

Ionic conductivity impedance [S/m]	1.52E-01	
charge carrier per unit cell	6.00	
unit cell volume [A^3]	425.60	
unit cell volume [m^3]	4.26E-28	
n charge carrier concentration [#/m3]	1.41E+28	
e^2 [C^2]	2.56697E-38	
k_b [J/K]	1.38E-23	
$D_{impedance}$	1.72E-12	
Jump process NMR	A	B
E_a NMR [eV]	1.31E-01	1.89E-01
τ [s]	3.34E-09	3.71E-08
D_{NMR} [m^2/s]	2.44E-12	2.19E-13

Table S6: Calculation of the diffusion coefficient from the Impedance measurement at room temperature and the corresponding diffusivities calculated from the fitting parameters from the NMR fits (Table S4). The jump process A contributes ~10 more to the diffusivity than the jump process B.

SI Text 1: Solid-state NMR for diffusion

Solid-state NMR can yield information about the diffusion mechanism in the material using following probing mechanisms:

- Fitting of the ln(1/T_1) vs. 1/k_BT curves gives access to the spectral density function, which is the Fourier transform of the correlation function of motion. This gives access to motion on the order of the inverse larmor frequency ($1/\omega_L \sim 10^{-8}$ s), hence is, in contrast to impedance spectroscopy, sensitive to back and forth hopping.
- The field dependence of T_1 measurements can be an indication of diffusion dimensionality[2].
- Fast and three dimensional motion would lead to motional narrowing of the line-shapes. If there is chemical shift anisotropy (CSA) in the structure, and the motion is not three dimensional, the CSA does not average out completely.

While diffusion in a real system is probably not perfectly one , two or three dimensional, the use of such models is helpful as they can be considered as an idealized description of anisotropic materials[3].

Spin lattice relaxation NMR

Spin-lattice relaxation times T_1 were recorded with a saturation recovery pulse sequence and fit with a single exponential function using the program ssnake[4]. This was repeated for the temperature range accessible on the setup; 20-200 C at 116.6 MHz ^{7}Li frequency (44MHz ^{6}Li) and -60 – 200C at a ^{7}Li frequency of 155.5 MHz.

The temperature dependence of the relaxation time is a complex curve governed by the motional correlation function (or it's Fourier transform, the spectral density J), and a functional depending on the spin interaction(s) in the material. Spin interactions are interactions that can lead to energy transfer between the observed spin and the external heat bath (the lattice). Most interactions are magnetic in nature (dipolar, chemical shift anisotropy, spin rotation), except for the quadrupole interaction which describes the interaction of the quadrupole moment of the spin>1/2 with electric field gradients in the material, which is an interaction of electric nature. As the chemical shift range is generally small for Li in dielectrics, the chemical shift anisotropy can be expected to lead to a minor contribution[5]. There are also no rotating polyatomic units in the structure, so also spin rotation can be excluded as a relaxation mechanism. This leaves dipole and quadrupole relaxation due to translational motion. The small quadrupole moment of ^{6}Li and ^{7}Li lead the system to relax effectively via the dipolar mechanism, a generally accepted fact in Li-NMR-relaxometry literature.

Relaxation due to dipolar interaction:

For the dipolar relaxation, the functional for translational motion has following functional:

$$\frac{1}{T_{1,D}} = K_D[J_1(\omega_0) + 4J_2(2(\omega_0)]$$

$$K_D = \frac{9}{8}\left(\frac{\mu_0 \gamma^2 \frac{h}{2\pi}}{4\pi}\right)^2$$

In this paper, we assumed the shape of the functional to be

$$\frac{1}{T_{1,sat}} = C[J_1(\omega_0) + 4J_2(2(\omega_0)]$$

Where C was used as a fitting parameter.

The more difficult problem lies at identifying the correct spectral density function. Many useful spectral densities are introduced in the review by Beckman[6]. In this report, we have used three spectral density functions. The spectral density first developed by Bloembergen, Purcell and Pound (BPP model) for three dimensional uncorrelated motion[7].

$$J(\omega) = \frac{\tau_c}{1 + \omega^2 \tau_c^2}$$

The BPP spectral density is a normalized form of the Fourier transform of an exponential correlation function[6]. Further, an empirical spectral density function was used which is a modified version of the BPP spectral density (MBPP)[8]. The spectral density was developed for layered conductor Na-β-Alumina. A parameter β is introduced, which accounts for correlations of the motion of the observed nuclei with the lattice or itself

$$J(\omega) = \frac{\tau_c}{1 + (\omega \tau_C)^{1+\beta}}$$

Due to the mathematical form of spectral density, it is not possible to calculate the corresponding time domain, as it is for the BPP spectral density. The parameter β describes a modification of the BPP model, but no physical model is behind the parameter.

A semi-empirical model for two dimensional diffusion was supposed by Richards (MR)[9]. Taking the limiting values into account, the spectral density results in the empirical expression[9,10]

$$J(\omega, \tau) = C * ln\left(1 + \frac{1}{\omega^2 {\tau_C}^2}\right)$$

The problem becomes very complex, when dimensionality effects appear at the same time as correlations due to coulomb interactions of the moving ions. While the dimensionality effects affect the high temperature slope of the curve, such correlation effects result in a reduced slope on the low temperature side. If both such effects are present, the peak can appear almost symmetric peak (like expected for the 3D case) can appear[10]. Such a spectral density also introduces a similar parameter β (MMR)

$$J(\omega, \tau) = C * ln\left(1 + \frac{1}{\omega^\beta {\tau_C}^\beta}\right)$$

The correlation time τ_c is (assumed to be) an exponential function in all the above spectral densities:

$$\tau_c = \tau_0 \exp\left(\frac{E_a}{k_b T}\right)$$

When plotting the natural logarithm of the inverse of T_1 against inverse temperature, the models predict a curve with two slopes on the low and high temperature end with opposite sign, and a maximum at the temperature where the inverse of the correlation time is in the same order as the Larmor frequency. The high-temperature slope can be used to calculate jump activation energies.

Measuring at a different field, and hence a different Larmor frequency, will shift the curve and display processes at different frequencies. This can be used to improve accuracy of the model by combined fitting, or in materials with different jump processes to vary the time scale of the probe. Li has two NMR accessible nuclei, ^{6}Li and ^{7}Li, to access larger changes in frequency at the same fields.

Using the correlation times τ obtained from the fit, and an estimate for the average jump distance a, the diffusion coefficient can be calculated:

$$D_{NMR} = \frac{a^2}{2d\tau_c}$$

Where d stands for the dimensionality of the diffusion process. To compare this entity with results from AC-impedance measurements, the diffusion coefficient from the impedance measurements can be calculated using the Nernst-Einstein relationship:

$$\sigma_{AC} = \frac{ne^2z^2D_{AC}}{k_B T}$$

Where n is the charge carrier density, e the electronic charge and z the charge of the diffusing ion. The diffusion coefficients from NMR and AC-impedance are measured on different time and length scales and do not necessarily match.

The diffusion coefficient measured from NMR is on the timescale of 10^8Hz, and is hence most comparable to the hopping diffusion coefficient used in theoretical work[12]:

$$D_{hopp} = \frac{1}{2}d^2\Gamma$$

Where d is the distance between hops of two sites and Γ the frequency those hops occur. The diffusion coefficient measured with AC-impedance, is on the order of the frequencies of the DC-plateau, so approximately 10^3-10^6 Hz. This diffusion coefficient is closer to the tracer diffusion coefficient, arising from the mean square displacement of the diffusion atom $\langle x^2 \rangle$ within a time t[12]

$$D_{tracer} = \frac{\langle x^2 \rangle}{2t}$$

Those two diffusion coefficients are theoretically corrected by the correlation factor f[12]

$$f = \frac{D_{tracer}}{D_{hop}}$$

The value of f can give information about possible rate limitations, correlations or the diffusion mechanism and is . In experimental studies these exact measurements are not accessible, and it is usually talked about the Haven Ratio HR

$$HR = \frac{D_{AC}}{D_{NMR}} \approx \frac{D_{long\ range}}{D_{short\ range}}$$

The HR is usually between 0 and 1, where 0 means no long range diffusion and only local hopping, and 1 means every individual jump probed with NMR leads to long range diffusion.

References

(1) Helm, B.; Schlem, R.; Wankmiller, B.; Banik, A.; Gautam, A.; Ruhl, J.; Li, C.; Hansen, M. R.; Zeier, W. G. Exploring Aliovalent Substitutions in the Lithium Halide Superionic Conductor $Li_{3-x}In_{1-x}Zr_xCl_6$ ($0 \leq x \leq 0.5$). *Chem. Mater.* **2021**, *33* (12), 4773–4782. https://doi.org/10.1021/acs.chemmater.1c01348.

(2) *Physics of Superionic Conductors*; Salamon, M. B., Ed.; Topics in Current Physics; Springer Berlin Heidelberg: Berlin, Heidelberg, 1979; Vol. 15. https://doi.org/10.1007/978-3-642-81328-3.

(3) Bjorkstam, J. L.; Villa, M. Second-Order Quadrupolar and Low-Dimensionality Effects upon NMR Resonance Spectra. *Phys. Rev. B* **1980**, *22* (11), 5025–5032. https://doi.org/10.1103/PhysRevB.22.5025.

(4) van Meerten, S. G. J.; Franssen, W. M. J.; Kentgens, A. P. M. SsNake: A Cross-Platform Open-Source NMR Data Processing and Fitting Application. *Journal of Magnetic Resonance* **2019**, *301*, 56–66. https://doi.org/10.1016/j.jmr.2019.02.006.

(5) Dong, R. Y. NMR Relaxation Rates. In *Encyclopedia of Spectroscopy and Spectrometry*; Elsevier, 2017; pp 1855–1862.

(6) Beckmann, P. A. Spectral Densities and Nuclear Spin Relaxation in Solids. *Physics Reports* **1988**, *171* (3), 85–128. https://doi.org/10.1016/0370-1573(88)90073-7.

(7) Bloembergen, N.; Purcell, E. M.; Pound, R. V. Relaxation Effects in Nuclear Magnetic Resonance Absorption. *Phys. Rev.* **1948**, *73* (7), 679–712. https://doi.org/10.1103/PhysRev.73.679.

(8) Böhmer, R.; Jeffrey, K. R.; Vogel, M. Solid-State Li NMR with Applications to the Translational Dynamics in Ion Conductors. *Progress in Nuclear Magnetic Resonance Spectroscopy* **2007**, *50* (2–3), 87–174. https://doi.org/10.1016/j.pnmrs.2006.12.001.

(9) Richards, P. M. Effect of Low Dimensionality on Prefactor Anomalies in Superionic Conductors. *Solid state communications* **1977**, *25*, 1019–1021.

(10) Kiichler, W.; Heitjans, P.; Payer, A.; Schiillhorn, R. 7Li NMR Relaxation by Diffusion in Hexagonal and Cubic Li,Ti&. 5.

(11) Newville, M.; Stensitzki, T.; Allen, D. B.; Ingargiola, A. LMFIT: Non-Linear Least-Square Minimization and Curve-Fitting for Python (0.8.0). *Zenodo* **2014**. https://doi.org/https://doi.org/10.5281/zenodo.11813.

(12) Mehrer, H. *Diffusion in Solids: Fundamentals, Methods, Materials, Diffusion-Controlled Processes*; Springer- Verlag Berlin Heidelberg, 2007.
