# Supplementary material for: Re-investigating the structure–property relationship of the solid electrolytes Li 3−xIn1−xZrxCl6 and the impact of In–Zr(iv) substitution
Source: J Mater Chem A Mater. 2023 Jan 25;11(9):4559–71. doi: 10.1039/d2ta08433c (PMC9969333; doi:10.1039/d2ta08433c)
Supplement: TA-011-D2TA08433C-s002 [file TA-011-D2TA08433C-s002.pdf]

Electronic Supplementary Material (ESI) for Journal of Materials Chemistry A.
This journal is © The Royal Society of Chemistry 2023

RelaxIS 3.0.20.16 - Report

Datasource: 50C_2.5_1

Circuit: I-R-P

Type	Value
**Temperature:**	50,000000
**Free variable:**	0,5000000
**DC Voltage:**	N/A
**AC Voltage:**	N/A
**Time:**	46,430562
**Harmonic:**	N/A
**Free Variable 2:**	N/A
**Area:**	N/A
**Thickness:**	N/A

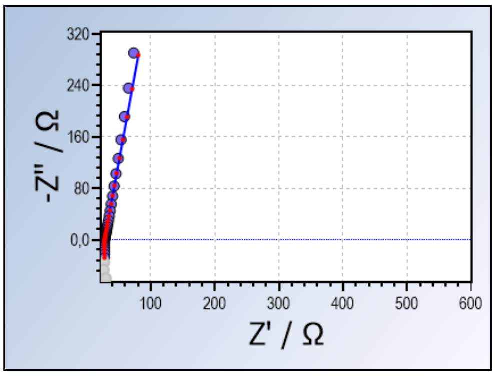

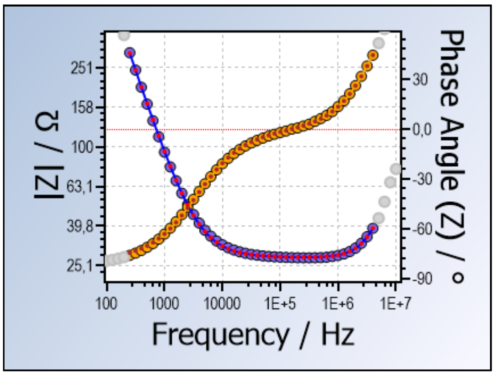

Fit Parameters:

Fix?	Name	Value	Error (Relative)
	Inductance 1	1,10E-006	7,04E-009 (0,6421160 %)
	Resistance 1	27,575348	0,0474837 (0,1721960 %)
	CPE Q 1	5,09E-006	6,55E-008 (1,2852221 %)
	CPE Alpha 1	0,8840629	0,0014415 (0,1630597 %)

RelaxIS 3.0.20.16 - Report

Datasource: 50C_2.6_1

Circuit: I-R-P

Type	Value
**Temperature:**	50,000000
**Free variable:**	0,4000000
**DC Voltage:**	N/A
**AC Voltage:**	N/A
**Time:**	46,279985
**Harmonic:**	N/A
**Free Variable 2:**	N/A
**Area:**	N/A
**Thickness:**	N/A

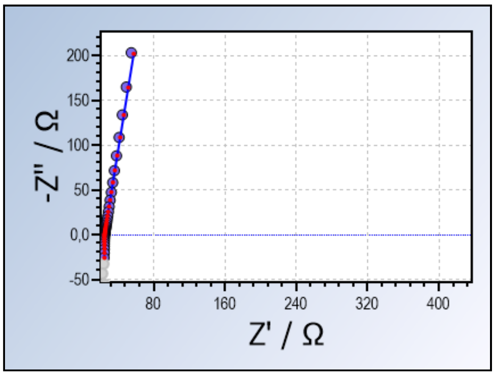

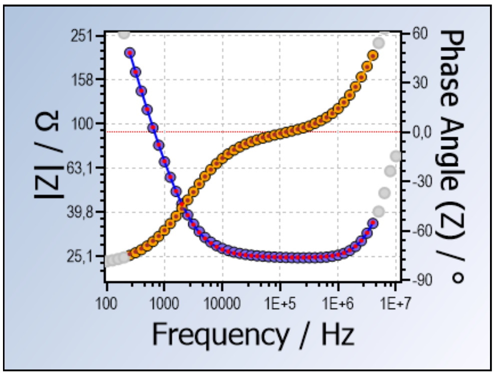

Fit Parameters:

Fix?	Name	Value	Error (Relative)
	Inductance 1	1,03E-006	4,60E-009 (0,4455372 %)
	Resistance 1	24,717289	0,0300316 (0,1215002 %)
	CPE Q 1	6,60E-006	6,54E-008 (0,9904691 %)
	CPE Alpha 1	0,8972797	0,0011272 (0,1256254 %)

RelaxIS 3.0.20.16 - Report

Datasource: 50C_2.7_1

Circuit: I-R-P

Type	Value
**Temperature:**	50,000000
**Free variable:**	0,3000000

**DC Voltage:**	N/A
**AC Voltage:**	N/A
**Time:**	46,569165
**Harmonic:**	N/A
**Free Variable 2:**	N/A
**Area:**	N/A
**Thickness:**	N/A

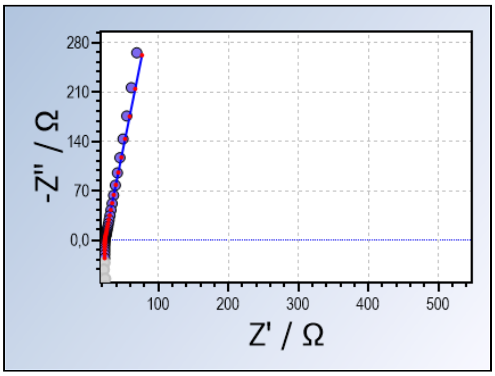

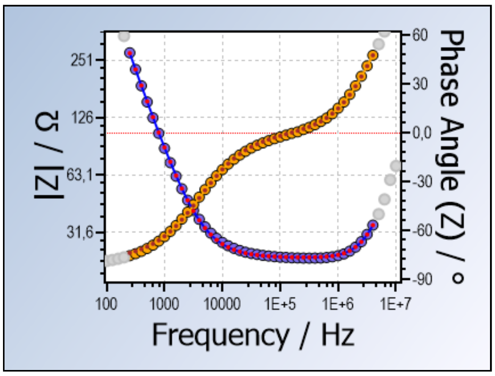

Fit Parameters:

Fix?	Name	Value	Error (Relative)
	Inductance 1	1,02E-006	7,45E-009 (0,7312372 %)
	Resistance 1	23,218833	0,0496692 (0,2139179 %)
	CPE Q 1	6,06E-006	9,28E-008 (1,5312715 %)
	CPE Alpha 1	0,8723153	0,0017075 (0,1957383 %)

RelaxIS 3.0.20.16 - Report

Datasource: 50C_2.8_1

Circuit: I-R-P

Type	Value
**Temperature:**	50,000000
**Free variable:**	0,2000000
**DC Voltage:**	N/A
**AC Voltage:**	N/A
**Time:**	46,580261
**Harmonic:**	N/A
**Free Variable 2:**	N/A
**Area:**	N/A
**Thickness:**	N/A

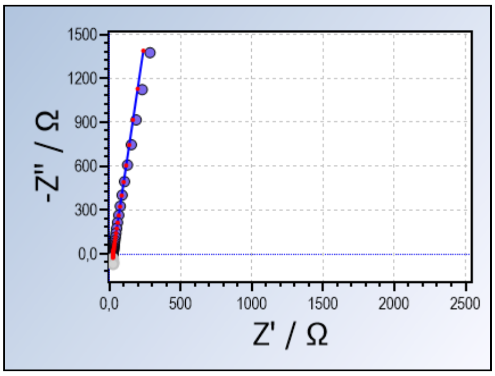

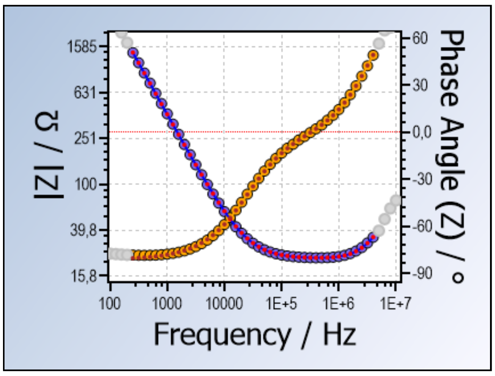

Fit Parameters:

Fix?	Name	Value	Error (Relative)
	Inductance 1	1,06E-006	5,46E-009 (0,5161454 %)
	Resistance 1	22,565144	0,0407126 (0,1804224 %)
	CPE Q 1	9,21E-007	7,56E-009 (0,8205577 %)
	CPE Alpha 1	0,9029763	0,0008340 (0,0923633 %)

RelaxIS 3.0.20.16 - Report

Datasource: 50C_2.9_1

Circuit: I-R-P

Type	Value
**Temperature:**	50,000000
**Free variable:**	0,1000000
**DC Voltage:**	N/A
**AC Voltage:**	N/A
**Time:**	46,486397
**Harmonic:**	N/A
**Free Variable 2:**	N/A
**Area:**	N/A
**Thickness:**	N/A

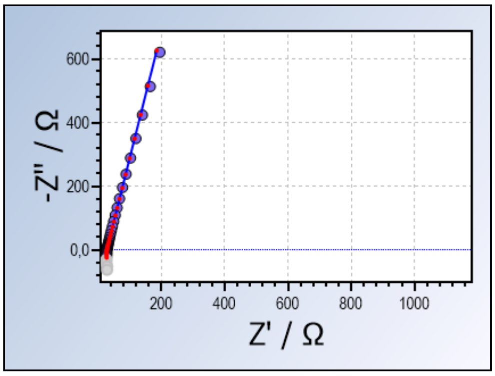

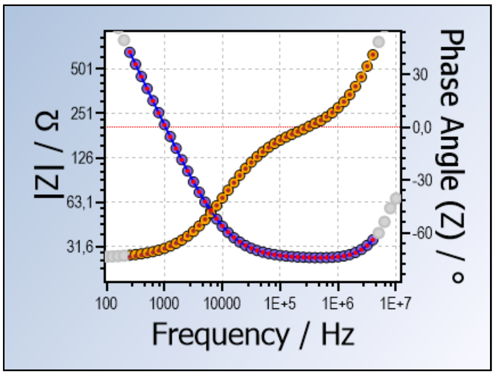

Fit Parameters:

Fix?	Name	Value	Error (Relative)
	Inductance 1	9,37E-007	3,41E-009 (0,3641185 %)
	Resistance 1	26,328812	0,0262509 (0,0997041 %)
	CPE Q 1	3,12E-006	1,72E-008 (0,5517557 %)
	CPE Alpha 1	0,8424853	0,0005860 (0,0695516 %)

RelaxIS 3.0.20.16 - Report

Datasource: 50C_3.0_1

Circuit: I-R-P

Type	Value
**Temperature:**	50,000000
**Free variable:**	0,0
**DC Voltage:**	N/A
**AC Voltage:**	N/A
**Time:**	46,483267
**Harmonic:**	N/A
**Free Variable 2:**	N/A
**Area:**	N/A
**Thickness:**	N/A

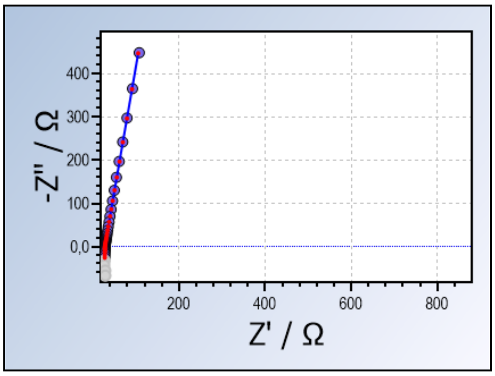

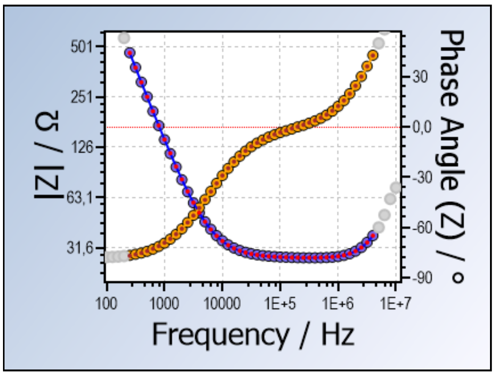

Fit Parameters:

Fix?	Name	Value	Error (Relative)
	Inductance 1	1,02E-006	2,52E-009 (0,2485828 %)
	Resistance 1	27,417958	0,0178738 (0,0651902 %)
	CPE Q 1	3,13E-006	1,34E-008 (0,4286441 %)
	CPE Alpha 1	0,8905184	0,0004688 (0,0526419 %)

RelaxIS 3.0.20.16 - Report

Datasource: 60C_2.5_1

Circuit: I-R-P

Type	Value
**Temperature:**	60,000000
**Free variable:**	0,5000000
**DC Voltage:**	N/A
**AC Voltage:**	N/A
**Time:**	46,529814
**Harmonic:**	N/A
**Free Variable 2:**	N/A
**Area:**	N/A
**Thickness:**	N/A

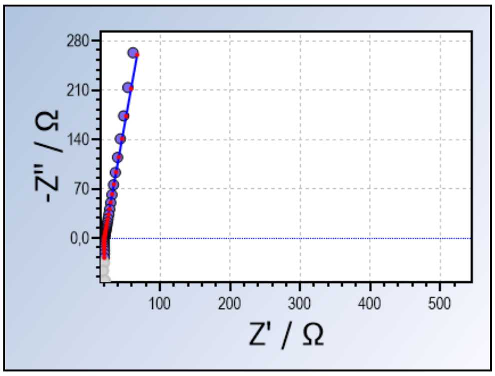

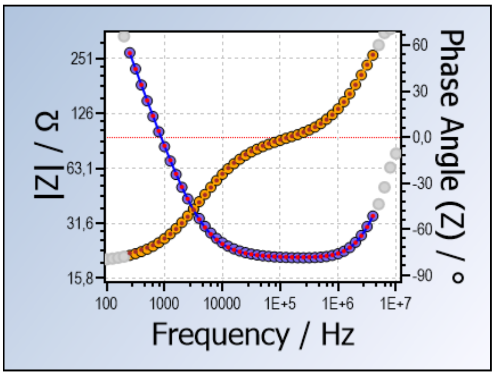

Fit Parameters:

Fix?	Name	Value	Error (Relative)
	Inductance 1	1,12E-006	5,93E-009 (0,5301031 %)
	Resistance 1	20,522551	0,0367478 (0,1790607 %)
	CPE Q 1	5,54E-006	6,87E-008 (1,2402096 %)
	CPE Alpha 1	0,8862980	0,0013765 (0,1553119 %)

RelaxIS 3.0.20.16 - Report

Datasource: 60C_2.6_1

Circuit: I-R-P

Type	Value
**Temperature:**	60,000000
**Free variable:**	0,4000000
**DC Voltage:**	N/A
**AC Voltage:**	N/A
**Time:**	46,464563
**Harmonic:**	N/A
**Free Variable 2:**	N/A
**Area:**	N/A
**Thickness:**	N/A

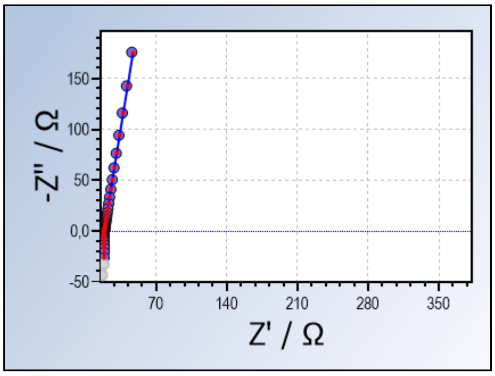

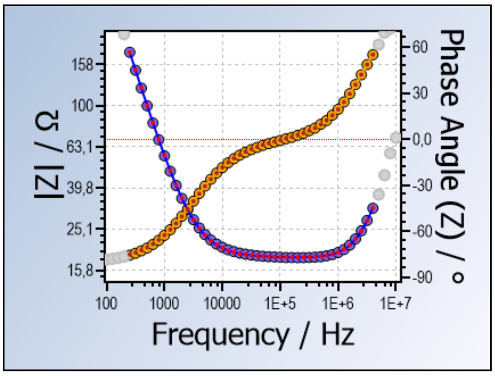

Fit Parameters:

Fix?	Name	Value	Error (Relative)
	Inductance 1	1,05E-006	2,72E-009 (0,2601292 %)
	Resistance 1	18,265904	0,0161445 (0,0883857 %)
	CPE Q 1	7,60E-006	5,11E-008 (0,6724827 %)
	CPE Alpha 1	0,8971356	0,0007586 (0,0845581 %)

RelaxIS 3.0.20.16 - Report

Datasource: 60C_2.7_1

Circuit: I-R-P

Type	Value
**Temperature:**	60,000000
**Free variable:**	0,3000000
**DC Voltage:**	N/A
**AC Voltage:**	N/A
**Time:**	46,024976
**Harmonic:**	N/A
**Free Variable 2:**	N/A
**Area:**	N/A
**Thickness:**	N/A

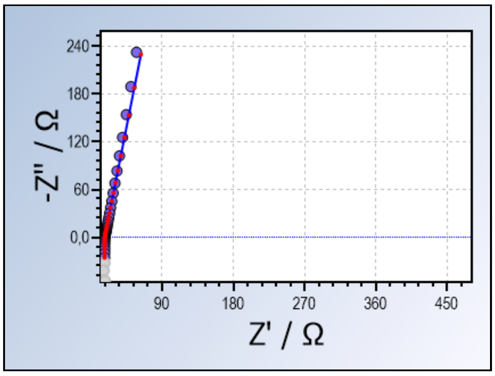

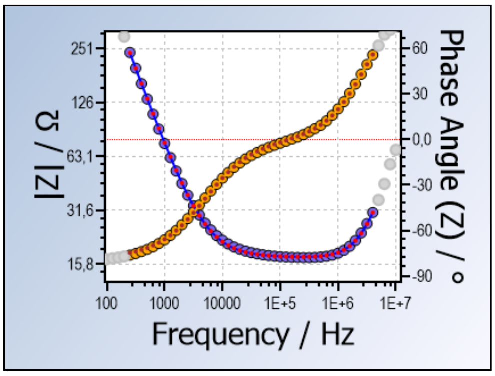

Fit Parameters:

Fix?	Name	Value	Error (Relative)
	Inductance 1	1,02E-006	5,91E-009 (0,5799018 %)
	Resistance 1	17,287058	0,0360573 (0,2085798 %)
	CPE Q 1	6,83E-006	9,56E-008 (1,4000171 %)
	CPE Alpha 1	0,8743751	0,0015477 (0,1770023 %)

RelaxIS 3.0.20.16 - Report

Datasource: 60C_2.8_1

Circuit: I-R-P

Type	Value
**Temperature:**	60,000000
**Free variable:**	0,2000000
**DC Voltage:**	N/A
**AC Voltage:**	N/A
**Time:**	46,100427
**Harmonic:**	N/A
**Free Variable**	N/A

**2:**	
**Area:**	N/A
**Thickness:**	N/A

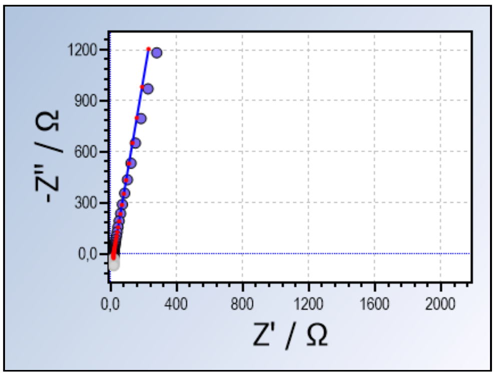

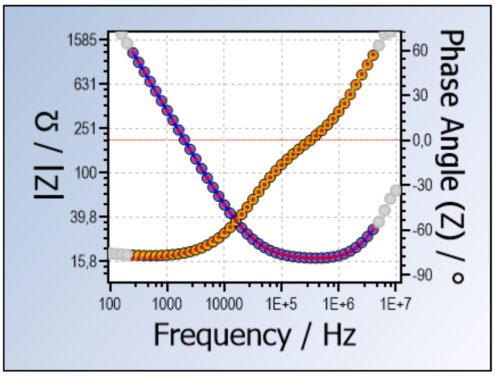

Fit Parameters:

Fix?	Name	Value	Error (Relative)
	Inductance 1	1,05E-006	6,40E-009 (0,6108628 %)
	Resistance 1	16,609563	0,0446261 (0,2686771 %)
	CPE Q 1	1,17E-006	1,31E-008 (1,1162578 %)
	CPE Alpha 1	0,8891532	0,0011216 (0,1261428 %)

RelaxIS 3.0.20.16 - Report

Datasource: 60C_2.9_1

Circuit: I-R-P

Type	Value
**Temperature:**	60,000000
**Free variable:**	0,1000000
**DC Voltage:**	N/A
**AC Voltage:**	N/A
**Time:**	46,083958
**Harmonic:**	N/A
**Free Variable 2:**	N/A
**Area:**	N/A
**Thickness:**	N/A

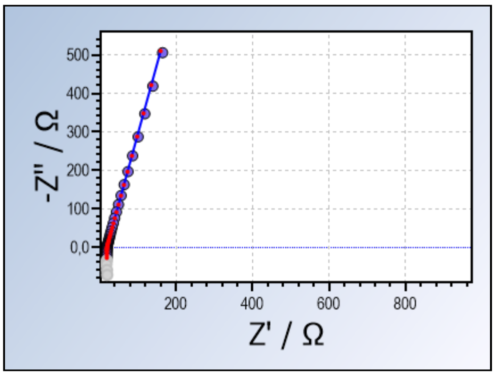

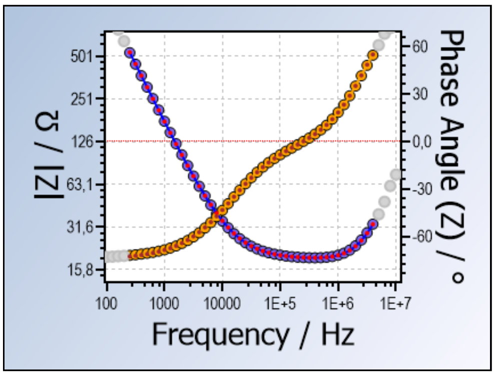

Fit Parameters:

Fix?	Name	Value	Error (Relative)
	Inductance 1	1,09E-006	2,67E-009 (0,2445345 %)
	Resistance 1	18,877392	0,0183223 (0,0970594 %)
	CPE Q 1	4,22E-006	2,07E-008 (0,4915953 %)
	CPE Alpha 1	0,8288661	0,0005174 (0,0624244 %)

RelaxIS 3.0.20.16 - Report

Datasource: 60C_3.0_1

Circuit: I-R-P

Type	Value
**Temperature:**	60,000000
**Free variable:**	0,0
**DC Voltage:**	N/A
**AC Voltage:**	N/A
**Time:**	46,504812
**Harmonic:**	N/A
**Free Variable 2:**	N/A
**Area:**	N/A
**Thickness:**	N/A

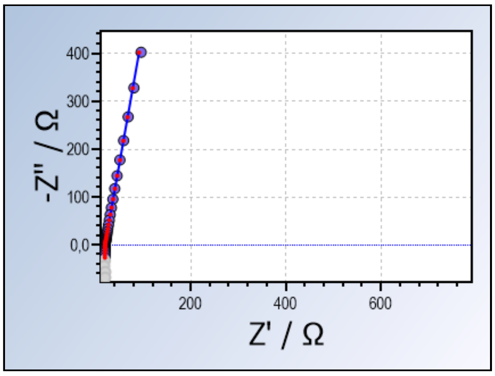

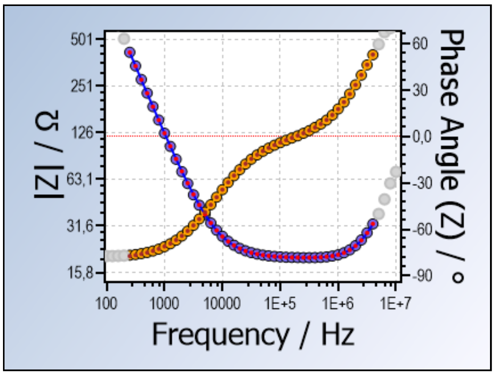

Fit Parameters:

Fix?	Name	Value	Error (Relative)
	Inductance 1	1,04E-006	1,91E-009 (0,1831540 %)
	Resistance 1	19,607559	0,0124369 (0,0634293 %)
	CPE Q 1	3,55E-006	1,35E-008 (0,3812391 %)
	CPE Alpha 1	0,8874981	0,0004114 (0,0463601 %)

RelaxIS 3.0.20.16 - Report

Datasource: 70C_2.5_1

Circuit: I-R-P

Type	Value
**Temperature:**	70,000000
**Free variable:**	0,5000000
**DC Voltage:**	N/A
**AC Voltage:**	N/A
**Time:**	46,375107
**Harmonic:**	N/A
**Free Variable 2:**	N/A
**Area:**	N/A
**Thickness:**	N/A

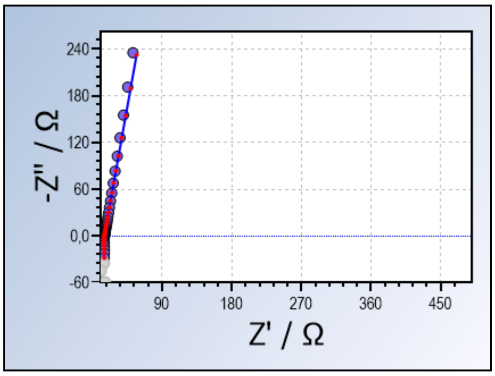

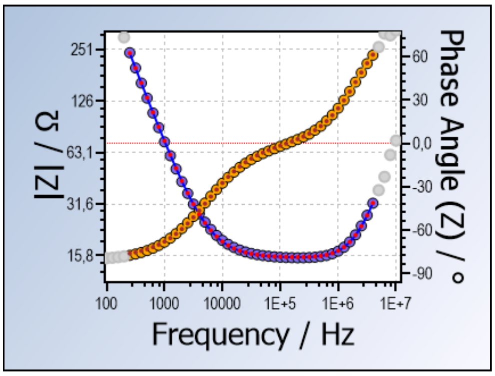

Fit Parameters:

Fix?	Name	Value	Error (Relative)
	Inductance 1	1,13E-006	5,22E-009 (0,4632273 %)
	Resistance 1	15,373573	0,0292011 (0,1899436 %)
	CPE Q 1	6,17E-006	7,55E-008 (1,2234295 %)
	CPE Alpha 1	0,8868513	0,0013448 (0,1516406 %)

RelaxIS 3.0.20.16 - Report

Datasource: 70C_2.6_1

Circuit: I-R-P

Type	Value
**Temperature:**	70,000000
**Free variable:**	0,4000000
**DC Voltage:**	N/A
**AC Voltage:**	N/A
**Time:**	48,077650
**Harmonic:**	N/A
**Free Variable 2:**	N/A
**Area:**	N/A
**Thickness:**	N/A

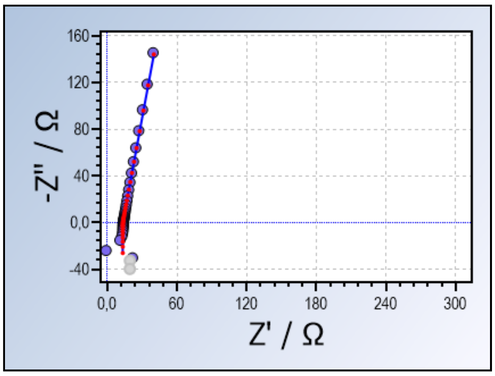

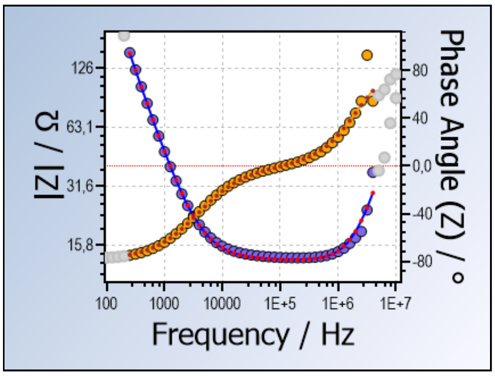

Fit Parameters:

Fix?	Name	Value	Error (Relative)
	Inductance 1	1,04E-006	4,10E-008 (3,9387795 %)
	Resistance 1	13,278711	0,2207090 (1,6621269 %)
	CPE Q 1	1,02E-005	1,20E-006 (11,697361 %)
	CPE Alpha 1	0,8828648	0,0130877 (1,4824101 %)

RelaxIS 3.0.20.16 - Report

Datasource: 70C_2.7_1

Circuit: I-R-P

Type	Value
**Temperature:**	70,000000
**Free variable:**	0,3000000
**DC Voltage:**	N/A
**AC Voltage:**	N/A
**Time:**	46,124254
**Harmonic:**	N/A
**Free Variable 2:**	N/A
**Area:**	N/A
**Thickness:**	N/A

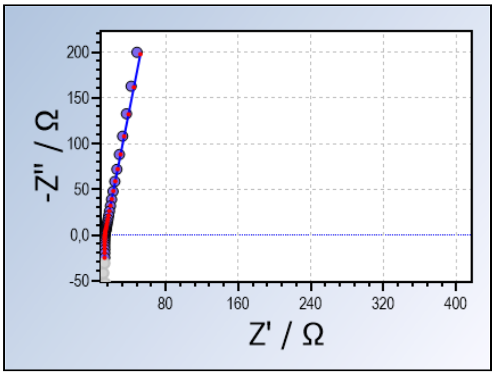

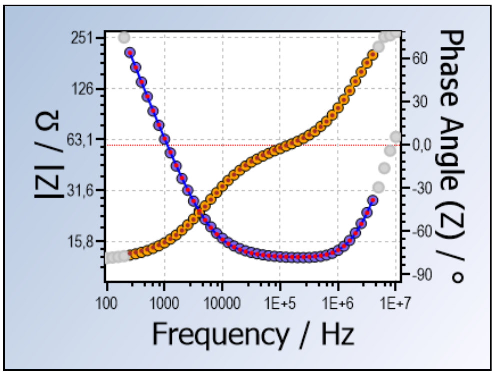

Fit Parameters:

Fix?	Name	Value	Error (Relative)
	Inductance 1	9,90E-007	4,39E-009 (0,4429653 %)
	Resistance 1	12,714339	0,0242062 (0,1903854 %)
	CPE Q 1	7,89E-006	9,41E-008 (1,1934853 %)
	CPE Alpha 1	0,8748934	0,0013078 (0,1494764 %)

RelaxIS 3.0.20.16 - Report

Datasource: 70C_2.8_1

Circuit: I-R-P

Type	Value
**Temperature:**	70,000000
**Free variable:**	0,2000000
**DC Voltage:**	N/A
**AC Voltage:**	N/A
**Time:**	46,447638
**Harmonic:**	N/A
**Free Variable 2:**	N/A
**Area:**	N/A
**Thickness:**	N/A

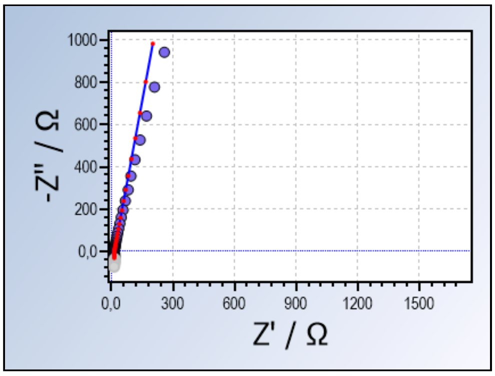

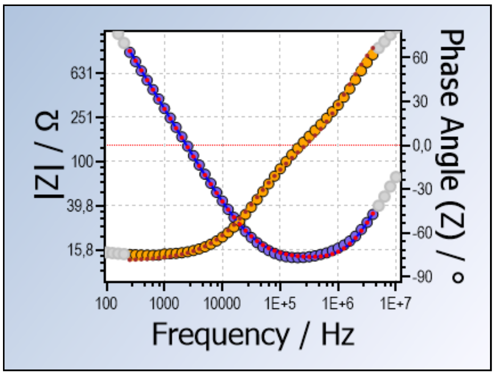

Fit Parameters:

Fix?	Name	Value	Error (Relative)
	Inductance 1	1,25E-006	2,31E-008 (1,8446422 %)
	Resistance 1	13,387204	0,1359515 (1,0155328 %)
	CPE Q 1	1,53E-006	6,08E-008 (3,9588168 %)
	CPE Alpha 1	0,8797645	0,0039707 (0,4513362 %)

RelaxIS 3.0.20.16 - Report

Datasource: 70C_2.9_1

Circuit: I-R-P

Type	Value
**Temperature:**	70,000000
**Free variable:**	0,1000000
**DC Voltage:**	N/A
**AC Voltage:**	N/A
**Time:**	47,902054
**Harmonic:**	N/A
**Free Variable**	N/A

**2:**	
**Area:**	N/A
**Thickness:**	N/A

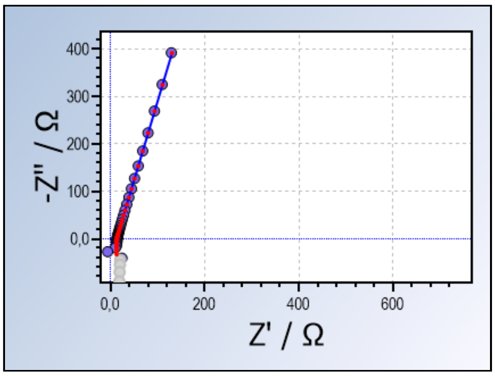

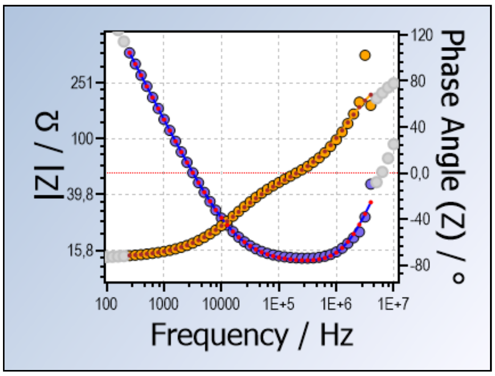

Fit Parameters:

Fix?	Name	Value	Error (Relative)
	Inductance 1	1,31E-006	5,17E-008 (3,9581274 %)
	Resistance 1	13,036310	0,2945726 (2,2596315 %)
	CPE Q 1	6,08E-006	6,19E-007 (10,186474 %)
	CPE Alpha 1	0,8143188	0,0106446 (1,3071755 %)

RelaxIS 3.0.20.16 - Report

Datasource: 70C_3.0_1

Circuit: I-R-P

Type	Value
**Temperature:**	70,000000
**Free variable:**	0,0
**DC Voltage:**	N/A
**AC Voltage:**	N/A
**Time:**	47,216634
**Harmonic:**	N/A
**Free Variable 2:**	N/A
**Area:**	N/A
**Thickness:**	N/A

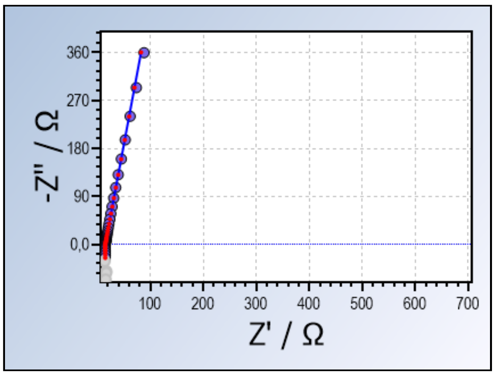

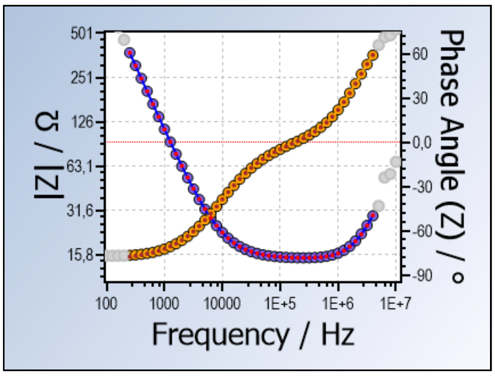

Fit Parameters:

Fix?	Name	Value	Error (Relative)
	Inductance 1	1,01E-006	2,09E-009 (0,2061825 %)
	Resistance 1	14,984800	0,0126779 (0,0846051 %)
	CPE Q 1	4,13E-006	1,96E-008 (0,4740170 %)
	CPE Alpha 1	0,8818344	0,0005066 (0,0574521 %)

RelaxIS 3.0.20.16 - Report

Datasource: 80C_2.5_1

Circuit: I-R-P

Type	Value
**Temperature:**	80,000000
**Free variable:**	0,5000000
**DC Voltage:**	N/A
**AC Voltage:**	N/A
**Time:**	46,101986
**Harmonic:**	N/A
**Free Variable 2:**	N/A
**Area:**	N/A
**Thickness:**	N/A

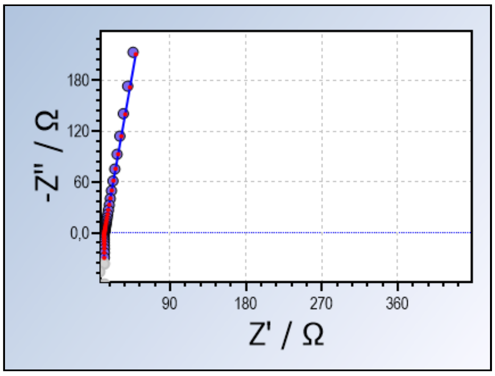

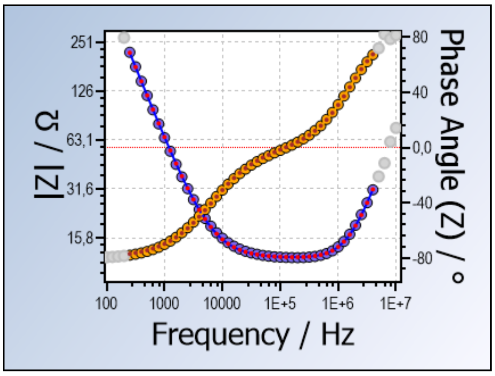

Fit Parameters:

Fix?	Name	Value	Error (Relative)
	Inductance 1	1,17E-006	4,46E-009 (0,3821629 %)
	Resistance 1	11,966199	0,0221461 (0,1850722 %)
	CPE Q 1	6,76E-006	7,54E-008 (1,1152395 %)
	CPE Alpha 1	0,8879814	0,0012159 (0,1369276 %)

RelaxIS 3.0.20.16 - Report

Datasource: 80C_2.6_1

Circuit: I-R-P

Type	Value
**Temperature:**	80,000000
**Free variable:**	0,4000000
**DC Voltage:**	N/A
**AC Voltage:**	N/A
**Time:**	48,139004
**Harmonic:**	N/A
**Free Variable 2:**	N/A
**Area:**	N/A
**Thickness:**	N/A

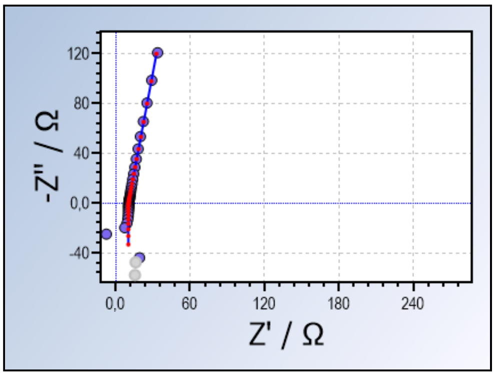

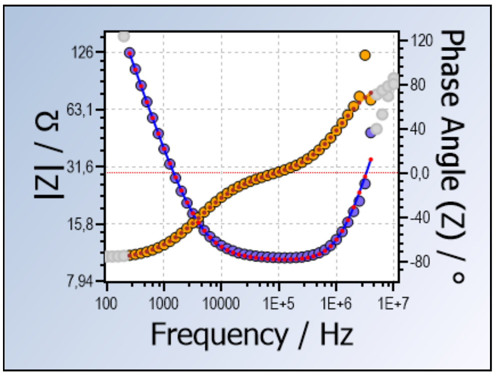

Fit Parameters:

Fix?	Name	Value	Error (Relative)
	Inductance 1	1,32E-006	4,69E-008 (3,5503349 %)
	Resistance 1	10,251250	0,1980530 (1,9319884 %)
	CPE Q 1	1,25E-005	1,59E-006 (12,713985 %)
	CPE Alpha 1	0,8809448	0,0141673 (1,6081909 %)

RelaxIS 3.0.20.16 - Report

Datasource: 80C_2.7_1

Circuit: I-R-P

Type	Value
**Temperature:**	80,000000
**Free variable:**	0,3000000
**DC Voltage:**	N/A
**AC Voltage:**	N/A
**Time:**	46,144798
**Harmonic:**	N/A
**Free Variable 2:**	N/A
**Area:**	N/A
**Thickness:**	N/A

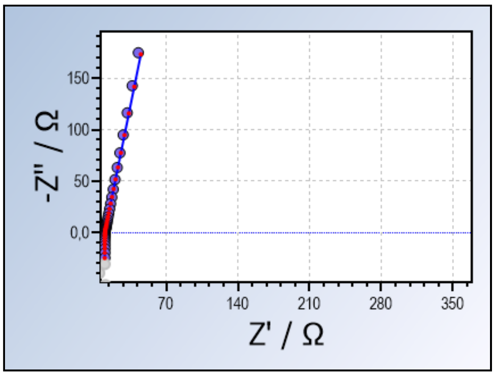

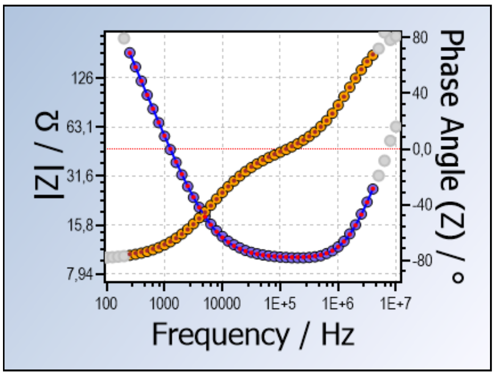

Fit Parameters:

Fix?	Name	Value	Error (Relative)
	Inductance 1	9,83E-007	3,61E-009 (0,3671863 %)
	Resistance 1	9,9551805	0,0180174 (0,1809851 %)
	CPE Q 1	9,17E-006	9,84E-008 (1,0724380 %)
	CPE Alpha 1	0,8722600	0,0011675 (0,1338463 %)

RelaxIS 3.0.20.16 - Report

Datasource: 80C_2.8_1

Circuit: I-R-P

Type	Value
**Temperature:**	80,000000
**Free variable:**	0,2000000
**DC Voltage:**	N/A
**AC Voltage:**	N/A
**Time:**	46,015404
**Harmonic:**	N/A
**Free Variable 2:**	N/A
**Area:**	N/A
**Thickness:**	N/A

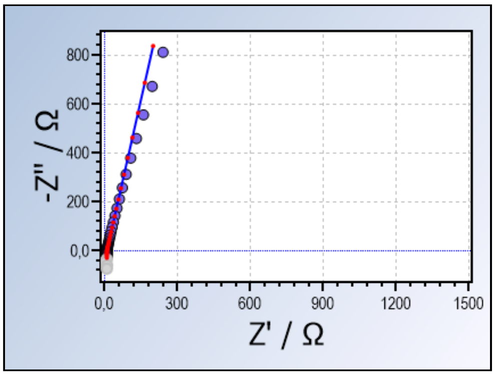

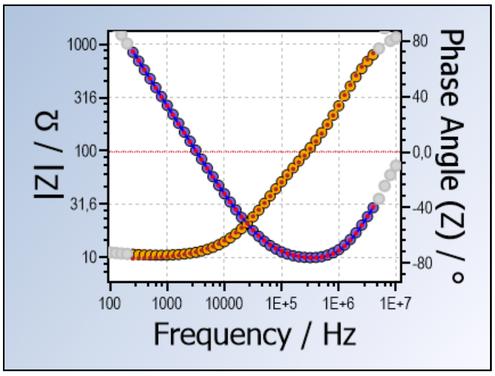

Fit Parameters:

Fix?	Name	Value	Error (Relative)
	Inductance 1	1,13E-006	7,94E-009 (0,7009272 %)
	Resistance 1	9,5033681	0,0448834 (0,4722894 %)
	CPE Q 1	2,11E-006	3,44E-008 (1,6347496 %)
	CPE Alpha 1	0,8575699	0,0016170 (0,1885511 %)

RelaxIS 3.0.20.16 - Report

Datasource: 80C_2.9_1

Circuit: I-R-P

Type	Value
**Temperature:**	80,000000
**Free variable:**	0,1000000
**DC Voltage:**	N/A
**AC Voltage:**	N/A
**Time:**	47,830360
**Harmonic:**	N/A
**Free Variable 2:**	N/A
**Area:**	N/A
**Thickness:**	N/A

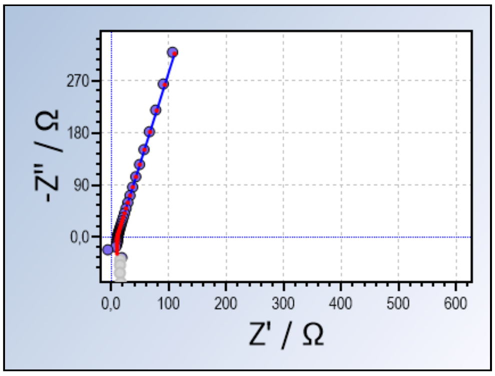

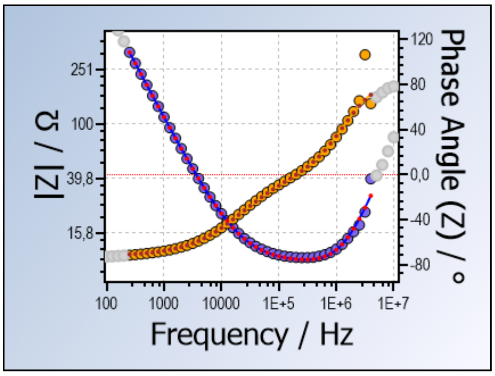

Fit Parameters:

Fix?	Name	Value	Error (Relative)
	Inductance 1	1,13E-006	4,27E-008 (3,7773694 %)
	Resistance 1	9,7763194	0,2325174 (2,3783736 %)
	CPE Q 1	8,00E-006	8,10E-007 (10,120395 %)
	CPE Alpha 1	0,8051586	0,0105191 (1,3064603 %)

RelaxIS 3.0.20.16 - Report

Datasource: 80C_3.0_1

Circuit: I-R-P

Type	Value
**Temperature:**	80,000000
**Free variable:**	0,0
**DC Voltage:**	N/A
**AC Voltage:**	N/A
**Time:**	46,134721
**Harmonic:**	N/A
**Free Variable**	N/A

**2:**	
**Area:**	N/A
**Thickness:**	N/A

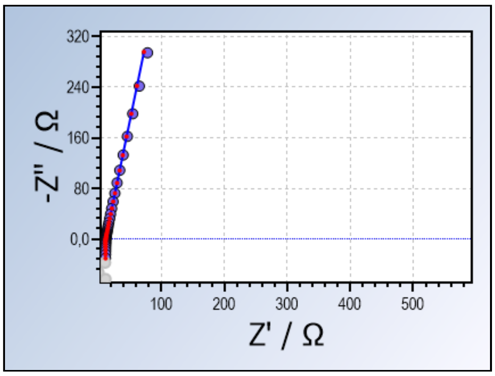

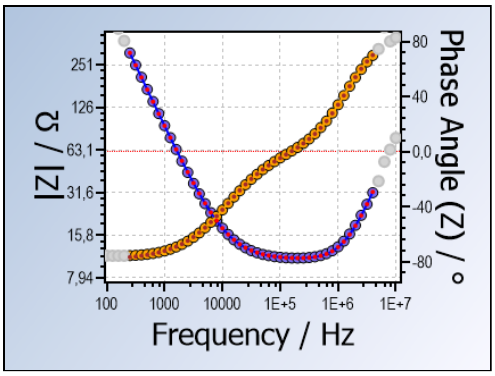

Fit Parameters:

Fix?	Name	Value	Error (Relative)
	Inductance 1	1,21E-006	2,61E-009 (0,2158660 %)
	Resistance 1	10,776954	0,0128730 (0,1194493 %)
	CPE Q 1	5,45E-006	3,31E-008 (0,6062470 %)
	CPE Alpha 1	0,8702271	0,0006423 (0,0738105 %)

RelaxIS 3.0.20.16 - Report

Datasource: 35C-2.5.txt_1

Circuit: I-(R)(C)-P

Type	Value
**Temperature:**	35,000000
**Free variable:**	0,5000000
**DC Voltage:**	N/A
**AC Voltage:**	N/A
**Time:**	46,426855
**Harmonic:**	N/A
**Free Variable 2:**	N/A
**Area:**	N/A
**Thickness:**	N/A

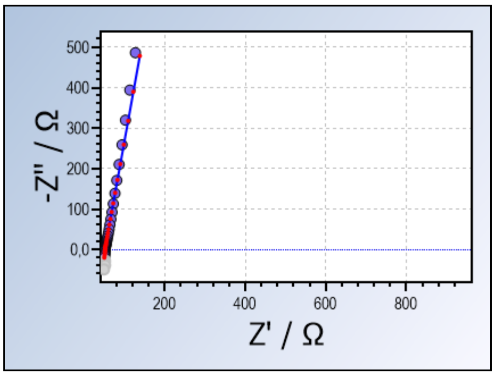

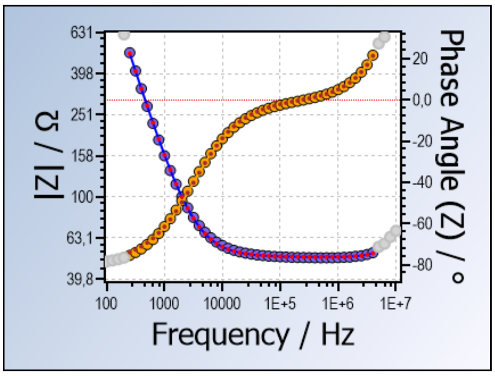

Fit Parameters:

Fix?	Name	Value	Error (Relative)
	Inductance 1	1,11E-006	4,91E-008 (4,4380929 %)
	Resistance 1	50,900516	0,0966160 (0,1898133 %)
	Capacitance 1	1,30E-010	1,92E-011 (14,763270 %)
	CPE Q 1	3,02E-006	4,20E-008 (1,3926969 %)
	CPE Alpha 1	0,8857453	0,0015721 (0,1774928 %)

RelaxIS 3.0.20.16 - Report

Datasource: 35C-2.6.txt_1

Circuit: I-(R)(C)-P

Type	Value
**Temperature:**	35,000000
**Free variable:**	0,4000000
**DC Voltage:**	N/A
**AC Voltage:**	N/A
**Time:**	46,654734
**Harmonic:**	N/A
**Free Variable 2:**	N/A
**Area:**	N/A
**Thickness:**	N/A

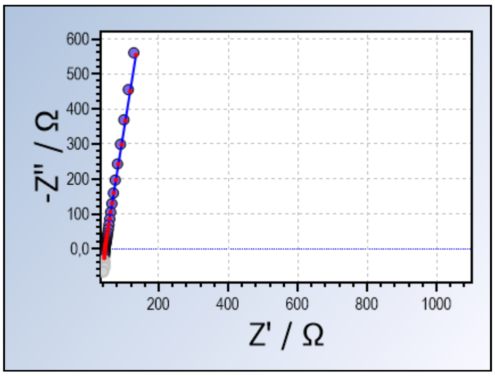

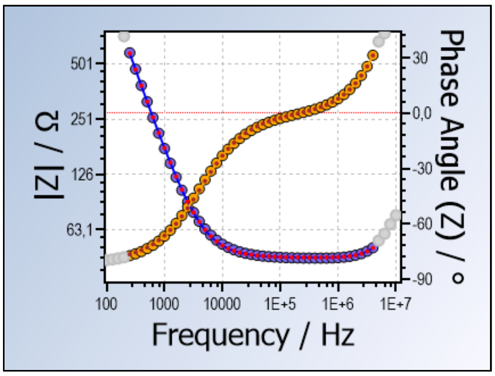

Fit Parameters:

Fix?	Name	Value	Error (Relative)
	Inductance 1	1,38E-006	3,36E-008 (2,4294139 %)
	Resistance 1	44,506398	0,0730773 (0,1641951 %)
	Capacitance 1	1,73E-010	1,72E-011 (9,9681174 %)
	CPE Q 1	2,40E-006	2,66E-008 (1,1110486 %)
	CPE Alpha 1	0,8976343	0,0012362 (0,1377128 %)

RelaxIS 3.0.20.16 - Report

Datasource: 35C-2.7.txt_1

Circuit: I-(R)(C)-P

Type	Value
**Temperature:**	35,000000
**Free variable:**	0,3000000
**DC Voltage:**	N/A
**AC Voltage:**	N/A
**Time:**	46,744449
**Harmonic:**	N/A
**Free Variable 2:**	N/A
**Area:**	N/A
**Thickness:**	N/A

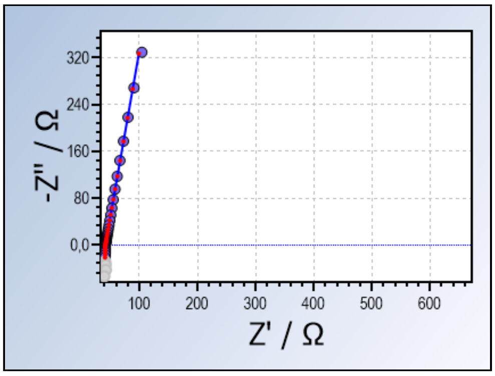

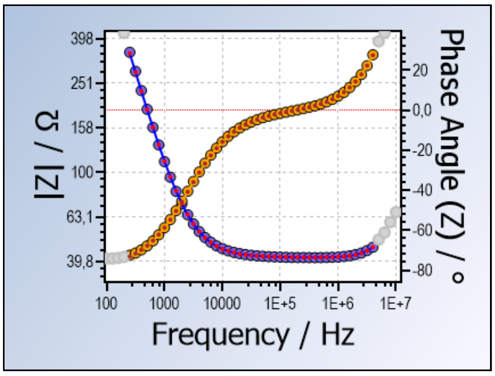

Fit Parameters:

Fix?	Name	Value	Error (Relative)
	Inductance 1	1,04E-006	2,31E-008 (2,2301713 %)
	Resistance 1	41,420811	0,0290327 (0,0700921 %)
	Capacitance 1	1,07E-010	1,35E-011 (12,693360 %)
	CPE Q 1	4,33E-006	2,37E-008 (0,5473253 %)
	CPE Alpha 1	0,8887754	0,0006232 (0,0701190 %)

RelaxIS 3.0.20.16 - Report

Datasource: 35C-2.9.txt_1

Circuit: I-(R)(C)-P

Type	Value
**Temperature:**	35,000000
**Free variable:**	0,1000000
**DC Voltage:**	N/A
**AC Voltage:**	N/A
**Time:**	46,569143
**Harmonic:**	N/A
**Free Variable 2:**	N/A
**Area:**	N/A

**Thickness:**	N/A

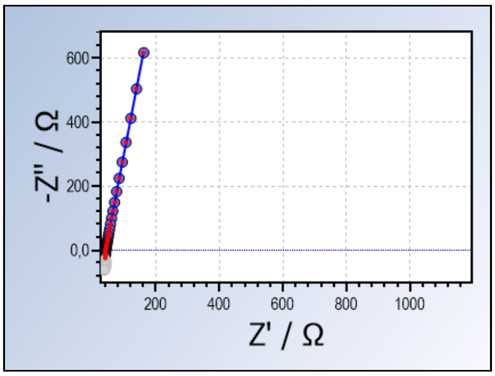

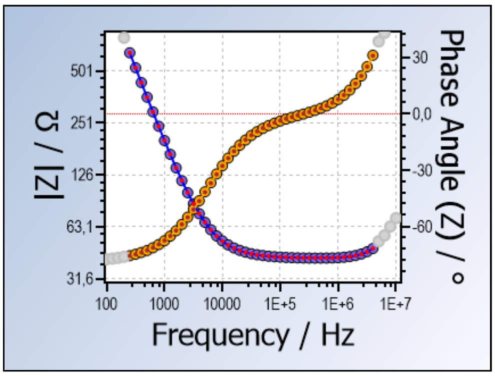

Fit Parameters:

Fix?	Name	Value	Error (Relative)
	Inductance 1	1,23E-006	1,22E-008 (0,9904096 %)
	Resistance 1	41,535215	0,0224969 (0,0541634 %)
	Capacitance 1	1,52E-010	7,16E-012 (4,7216485 %)
	CPE Q 1	2,46E-006	8,35E-009 (0,3389644 %)
	CPE Alpha 1	0,8786473	0,0003725 (0,0423911 %)

RelaxIS 3.0.20.16 - Report

Datasource: 50C-2.5.txt_1

Circuit: I-(R)(C)-P

Type	Value
**Temperature:**	50,000000
**Free variable:**	0,5000000
**DC Voltage:**	N/A
**AC Voltage:**	N/A
**Time:**	46,555848
**Harmonic:**	N/A
**Free Variable 2:**	N/A
**Area:**	N/A
**Thickness:**	N/A

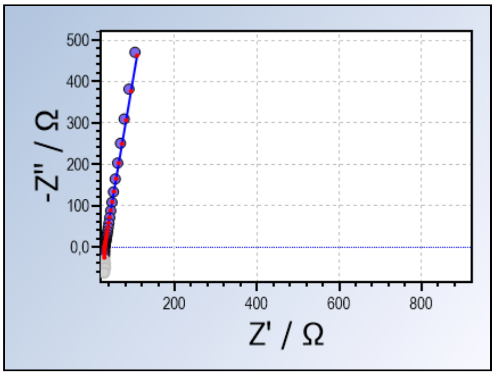

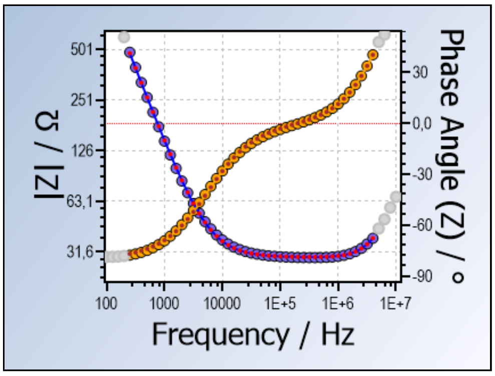

Fit Parameters:

Fix?	Name	Value	Error (Relative)
	Inductance 1	1,11E-006	5,47E-008 (4,9455645 %)
	Resistance 1	29,312834	0,0585825 (0,1998527 %)
	Capacitance 1	1,37E-010	6,40E-011 (46,772049 %)
	CPE Q 1	2,99E-006	3,71E-008 (1,2433083 %)
	CPE Alpha 1	0,8920742	0,0013642 (0,1529285 %)

RelaxIS 3.0.20.16 - Report

Datasource: 50C-2.6.txt_1

Circuit: I-(R)(C)-P

Type	Value
**Temperature:**	50,000000
**Free variable:**	0,4000000
**DC Voltage:**	N/A
**AC Voltage:**	N/A
**Time:**	46,448463
**Harmonic:**	N/A
**Free Variable 2:**	N/A
**Area:**	N/A

**Thickness:**	N/A

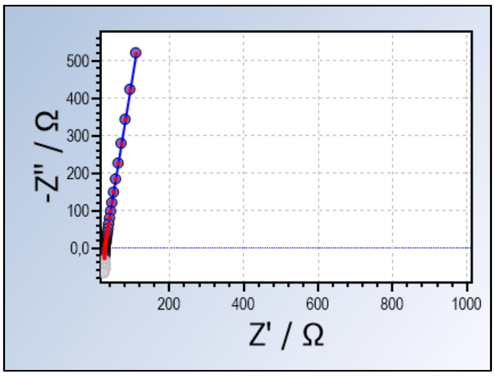

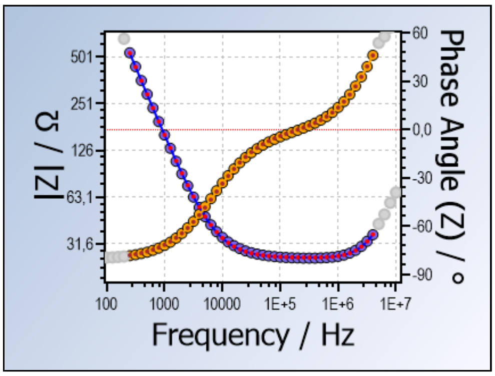

Fit Parameters:

Fix?	Name	Value	Error (Relative)
	Inductance 1	1,20E-006	2,64E-008 (2,2075399 %)
	Resistance 1	25,674864	0,0375345 (0,1461917 %)
	Capacitance 1	2,18E-010	4,04E-011 (18,505613 %)
	CPE Q 1	2,58E-006	2,18E-008 (0,8439895 %)
	CPE Alpha 1	0,8963801	0,0009138 (0,1019385 %)

RelaxIS 3.0.20.16 - Report

Datasource: 50C-2.7.txt_1

Circuit: I-(R)(C)-P

Type	Value
**Temperature:**	50,000000
**Free variable:**	0,3000000
**DC Voltage:**	N/A
**AC Voltage:**	N/A
**Time:**	46,363335
**Harmonic:**	N/A
**Free Variable 2:**	N/A
**Area:**	N/A
**Thickness:**	N/A

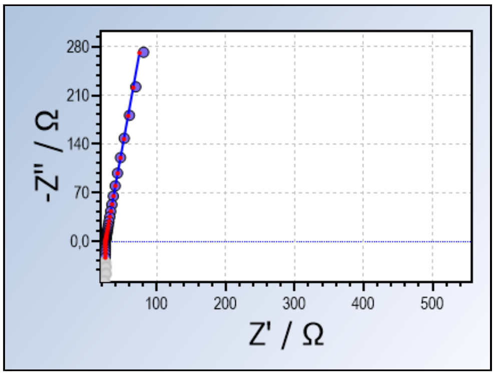

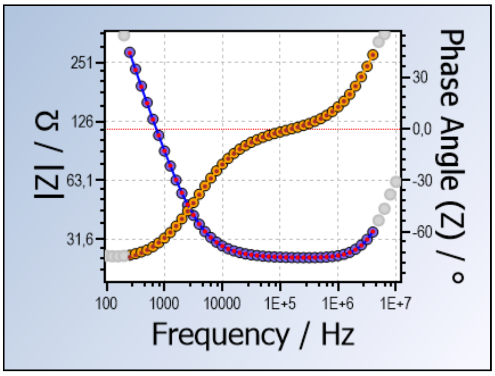

Fit Parameters:

Fix?	Name	Value	Error (Relative)
	Inductance 1	1,01E-006	3,74E-008 (3,6979405 %)
	Resistance 1	25,444099	0,0235919 (0,0927207 %)
	Capacitance 1	9,78E-011	5,78E-011 (59,156822 %)
	CPE Q 1	5,36E-006	3,47E-008 (0,6479978 %)
	CPE Alpha 1	0,8850393	0,0007254 (0,0819622 %)

RelaxIS 3.0.20.16 - Report

Datasource: 50C-2.9.txt_1

Circuit: I-(R)(C)-P

Type	Value
**Temperature:**	50,000000
**Free variable:**	0,1000000
**DC Voltage:**	N/A
**AC Voltage:**	N/A
**Time:**	46,959170
**Harmonic:**	N/A
**Free Variable 2:**	N/A
**Area:**	N/A

**Thickness:**	N/A

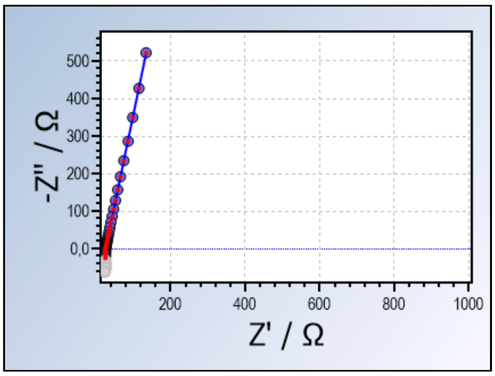

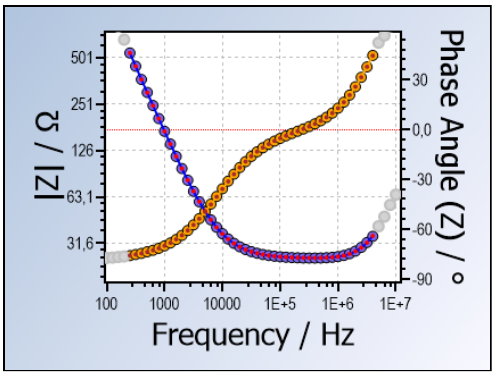

Fit Parameters:

Fix?	Name	Value	Error (Relative)
	Inductance 1	1,06E-006	1,61E-008 (1,5098371 %)
	Resistance 1	25,220597	0,0111304 (0,0441320 %)
	Capacitance 1	1,03E-010	2,53E-011 (24,462230 %)
	CPE Q 1	3,13E-006	7,63E-009 (0,2438954 %)
	CPE Alpha 1	0,8686692	0,0002627 (0,0302397 %)

RelaxIS 3.0.20.16 - Report

Datasource: 65C-2.5.txt_1

Circuit: I-(R)(C)-P

Type	Value
**Temperature:**	65,000000
**Free variable:**	0,5000000
**DC Voltage:**	N/A
**AC Voltage:**	N/A
**Time:**	46,834743
**Harmonic:**	N/A
**Free Variable 2:**	N/A
**Area:**	N/A
**Thickness:**	N/A

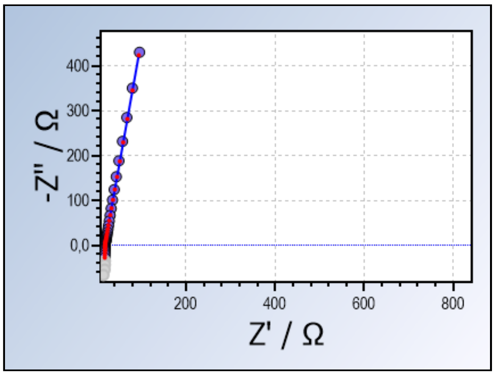

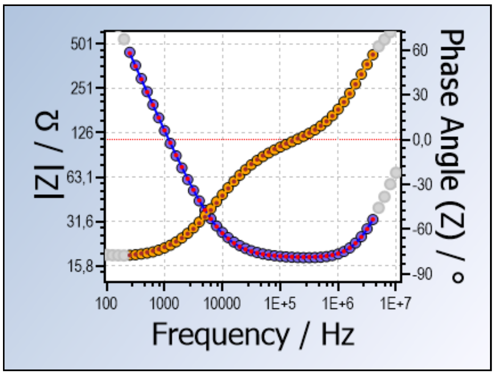

Fit Parameters:

Fix?	Name	Value	Error (Relative)
	Inductance 1	1,18E-006	4,07E-008 (3,4498690 %)
	Resistance 1	18,022201	0,0388497 (0,2155656 %)
	Capacitance 1	2,44E-010	1,26E-010 (51,673279 %)
	CPE Q 1	3,39E-006	3,94E-008 (1,1638223 %)
	CPE Alpha 1	0,8869013	0,0012475 (0,1406561 %)

RelaxIS 3.0.20.16 - Report

Datasource: 65C-2.6.txt_1

Circuit: I-(R)(C)-P

Type	Value
**Temperature:**	65,000000
**Free variable:**	0,4000000
**DC Voltage:**	N/A
**AC Voltage:**	N/A
**Time:**	46,850815
**Harmonic:**	N/A
**Free Variable 2:**	N/A
**Area:**	N/A

**Thickness:**	N/A

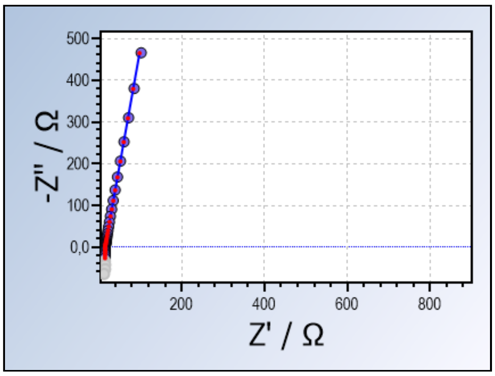

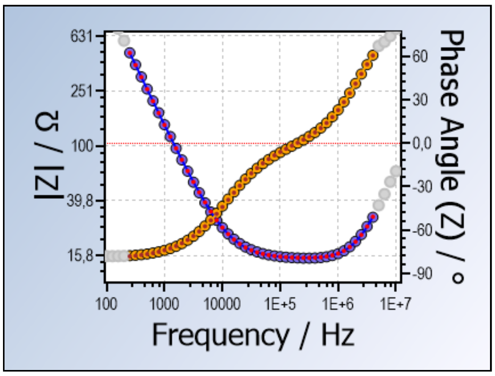

Fit Parameters:

Fix?	Name	Value	Error (Relative)
	Inductance 1	1,12E-006	2,19E-008 (1,9500771 %)
	Resistance 1	15,083111	0,0184834 (0,1225436 %)
	Capacitance 1	2,70E-010	9,68E-011 (35,922029 %)
	CPE Q 1	3,07E-006	1,86E-008 (0,6045673 %)
	CPE Alpha 1	0,8873002	0,0006382 (0,0719292 %)

RelaxIS 3.0.20.16 - Report

Datasource: 65C-2.7.txt_1

Circuit: I-(R)(C)-P

Type	Value
**Temperature:**	65,000000
**Free variable:**	0,3000000
**DC Voltage:**	N/A
**AC Voltage:**	N/A
**Time:**	46,345473
**Harmonic:**	N/A
**Free Variable 2:**	N/A
**Area:**	N/A
**Thickness:**	N/A

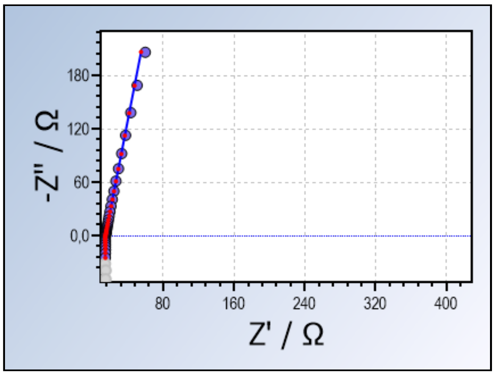

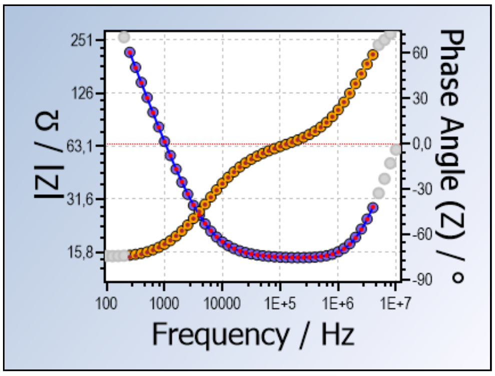

Fit Parameters:

Fix?	Name	Value	Error (Relative)
	Inductance 1	9,82E-007	1,30E-007 (13,199880 %)
	Resistance 1	14,677919	0,0152765 (0,1040784 %)
	Capacitance 1	1,11E-015	6,00E-010 (5,41E+007 %)
	CPE Q 1	7,45E-006	5,09E-008 (0,6838111 %)
	CPE Alpha 1	0,8770801	0,0007536 (0,0859179 %)

RelaxIS 3.0.20.16 - Report

Datasource: 65C-2.9.txt_1

Circuit: I-(R)(C)-P

Type	Value
**Temperature:**	65,000000
**Free variable:**	0,1000000
**DC Voltage:**	N/A
**AC Voltage:**	N/A
**Time:**	47,007935
**Harmonic:**	N/A
**Free Variable 2:**	N/A
**Area:**	N/A

**Thickness:**	N/A

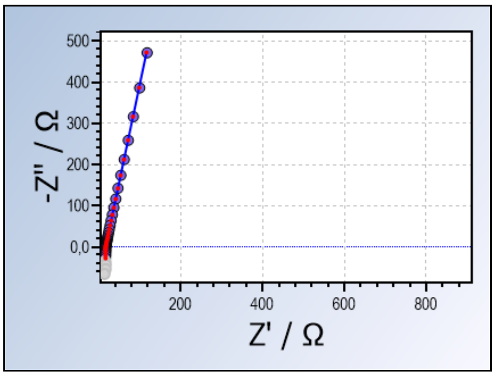

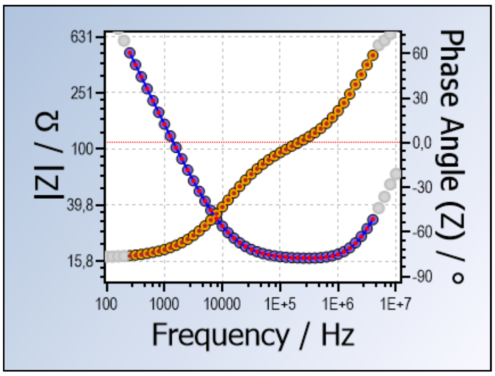

Fit Parameters:

Fix?	Name	Value	Error (Relative)
	Inductance 1	1,09E-006	9,54E-008 (8,7773680 %)
	Resistance 1	16,410146	0,0096309 (0,0586889 %)
	Capacitance 1	1,06E-015	3,55E-010 (3,35E+007 %)
	CPE Q 1	3,49E-006	1,07E-008 (0,3067717 %)
	CPE Alpha 1	0,8670409	0,0003247 (0,0374546 %)

RelaxIS 3.0.20.16 - Report

Datasource: 65C-3.0.txt_1

Circuit: I-(R)(C)-P

Type	Value
**Temperature:**	65,000000
**Free variable:**	0,0
**DC Voltage:**	N/A
**AC Voltage:**	N/A
**Time:**	46,867587
**Harmonic:**	N/A
**Free Variable 2:**	N/A
**Area:**	N/A
**Thickness:**	N/A

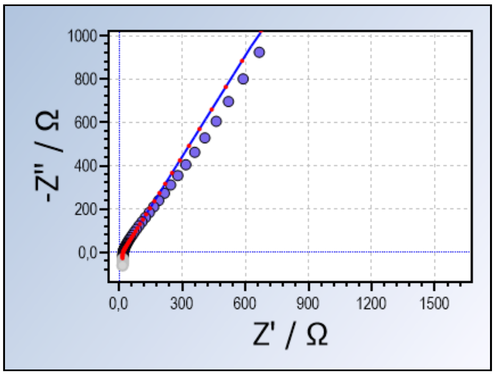

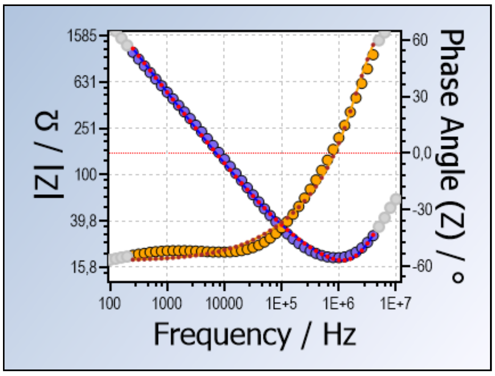

Fit Parameters:

Fix?	Name	Value	Error (Relative)
	Inductance 1	1,11E-006	1,41E-006 (127,80851 %)
	Resistance 1	14,813050	0,3757537 (2,5366395 %)
	Capacitance 1	1,00E-015	6,39E-009 (6,39E+008 %)
	CPE Q 1	7,60E-006	3,91E-007 (5,1543454 %)
	CPE Alpha 1	0,6358814	0,0049669 (0,7810972 %)

RelaxIS 3.0.20.16 - Report

Datasource: 30C_2.5_1

Circuit: I-(R)(C)-P

Type	Value
**Temperature:**	30,000000
**Free variable:**	0,5000000
**DC Voltage:**	N/A
**AC Voltage:**	N/A
**Time:**	46,475525
**Harmonic:**	N/A
**Free Variable 2:**	N/A
**Area:**	N/A

**Thickness:**	N/A

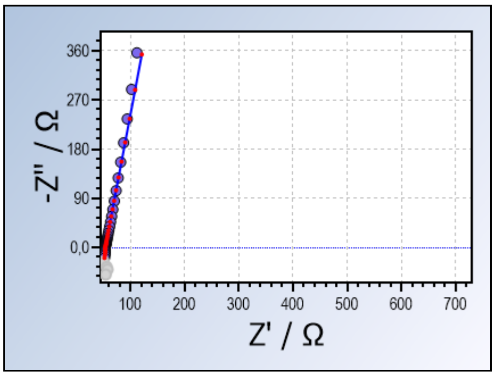

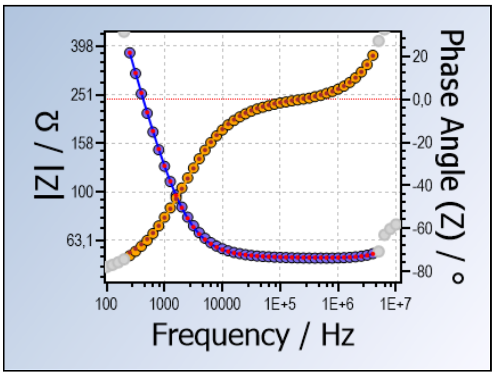

Fit Parameters:

Fix?	Name	Value	Error (Relative)
	Inductance 1	1,12E-006	3,94E-008 (3,5095418 %)
	Resistance 1	53,562010	0,0773724 (0,1444539 %)
	Capacitance 1	1,25E-010	1,39E-011 (11,136964 %)
	CPE Q 1	4,26E-006	5,06E-008 (1,1880637 %)
	CPE Alpha 1	0,8800706	0,0013668 (0,1553110 %)

RelaxIS 3.0.20.16 - Report

Datasource: 30C_2.6_1

Circuit: I-(R)(C)-P

Type	Value
**Temperature:**	30,000000
**Free variable:**	0,4000000
**DC Voltage:**	N/A
**AC Voltage:**	N/A
**Time:**	45,954356
**Harmonic:**	N/A
**Free Variable 2:**	N/A
**Area:**	N/A
**Thickness:**	N/A

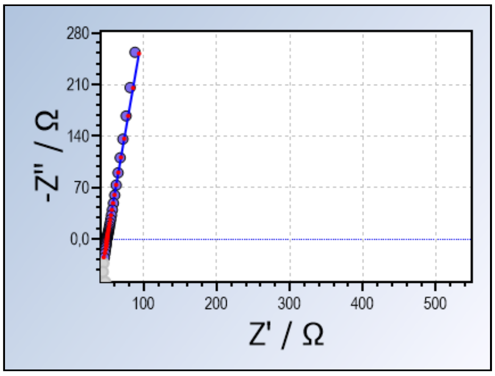

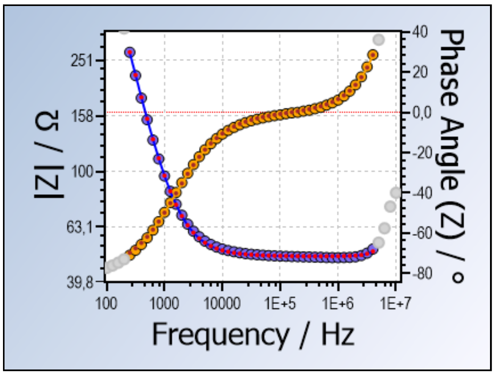

Fit Parameters:

Fix?	Name	Value	Error (Relative)
	Inductance 1	1,54E-006	2,53E-008 (1,6447429 %)
	Resistance 1	49,459236	0,0837050 (0,1692404 %)
	Capacitance 1	2,45E-010	1,07E-011 (4,3790093 %)
	CPE Q 1	5,57E-006	8,59E-008 (1,5410958 %)
	CPE Alpha 1	0,8897323	0,0017987 (0,2021575 %)

RelaxIS 3.0.20.16 - Report

Datasource: 30C_2.7_1

Circuit: I-(R)(C)-P

Type	Value
**Temperature:**	30,000000
**Free variable:**	0,3000000
**DC Voltage:**	N/A
**AC Voltage:**	N/A
**Time:**	45,913954
**Harmonic:**	N/A
**Free Variable 2:**	N/A
**Area:**	N/A

**Thickness:**	N/A

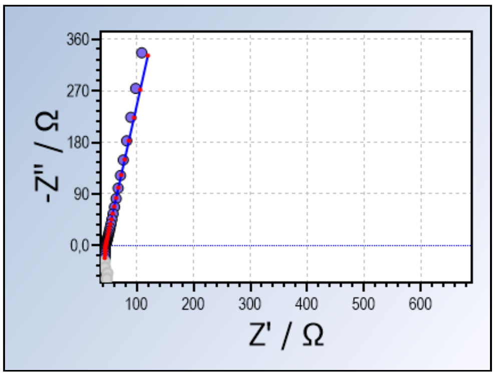

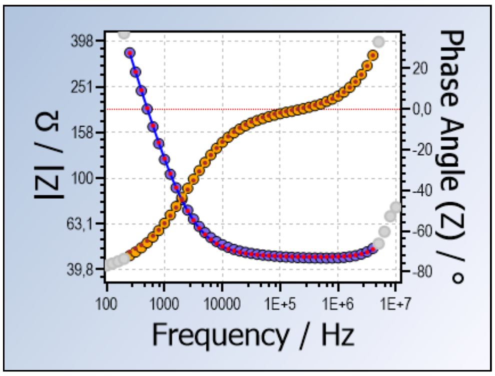

Fit Parameters:

Fix?	Name	Value	Error (Relative)
	Inductance 1	1,18E-006	5,19E-008 (4,4082517 %)
	Resistance 1	45,080765	0,1049622 (0,2328315 %)
	Capacitance 1	1,55E-010	2,59E-011 (16,663034 %)
	CPE Q 1	5,25E-006	9,47E-008 (1,8045934 %)
	CPE Alpha 1	0,8593816	0,0020598 (0,2396835 %)

RelaxIS 3.0.20.16 - Report

Datasource: 30C_2.8_1

Circuit: I-(R)(C)-P

Type	Value
**Temperature:**	30,000000
**Free variable:**	0,2000000
**DC Voltage:**	N/A
**AC Voltage:**	N/A
**Time:**	46,170036
**Harmonic:**	N/A
**Free Variable 2:**	N/A
**Area:**	N/A
**Thickness:**	N/A

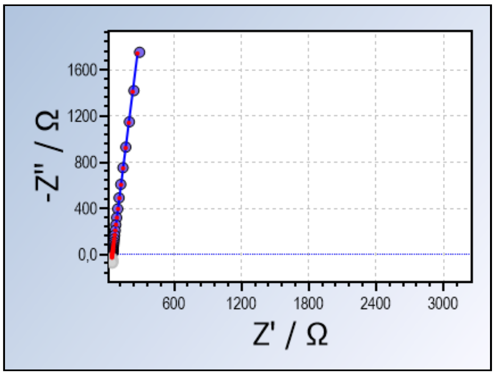

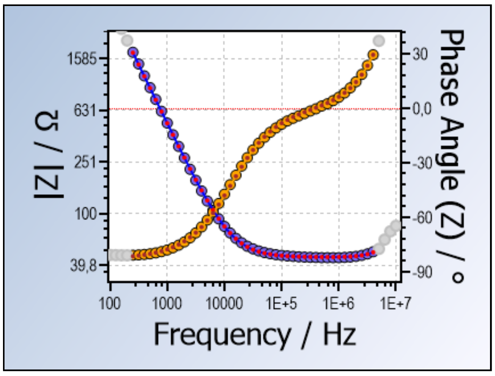

Fit Parameters:

Fix?	Name	Value	Error (Relative)
	Inductance 1	1,42E-006	2,97E-008 (2,0984660 %)
	Resistance 1	46,121203	0,0853852 (0,1851322 %)
	Capacitance 1	2,02E-010	1,43E-011 (7,0774835 %)
	CPE Q 1	6,58E-007	6,08E-009 (0,9246321 %)
	CPE Alpha 1	0,9182072	0,0009692 (0,1055490 %)

RelaxIS 3.0.20.16 - Report

Datasource: 30C_2.9_1

Circuit: I-(R)(C)-P

Type	Value
**Temperature:**	30,000000
**Free variable:**	0,1000000
**DC Voltage:**	N/A
**AC Voltage:**	N/A
**Time:**	46,276883
**Harmonic:**	N/A
**Free Variable 2:**	N/A
**Area:**	N/A

**Thickness:**	N/A

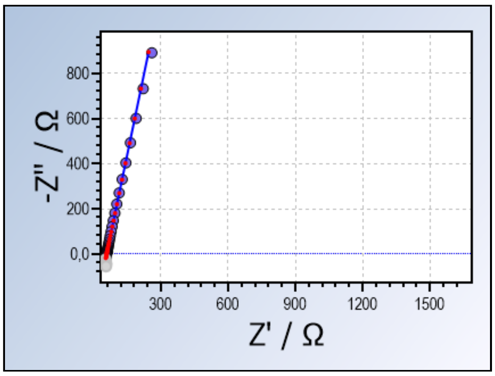

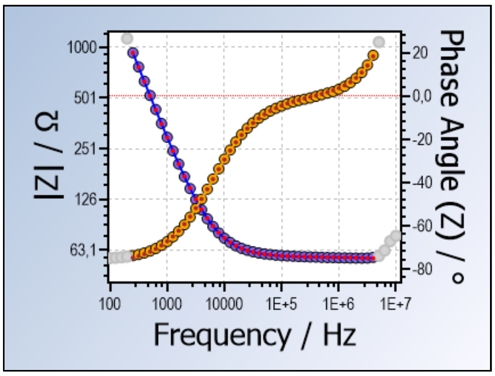

Fit Parameters:

Fix?	Name	Value	Error (Relative)
	Inductance 1	1,27E-006	2,00E-008 (1,5709481 %)
	Resistance 1	57,102532	0,0660076 (0,1155949 %)
	Capacitance 1	1,82E-010	6,32E-012 (3,4654594 %)
	CPE Q 1	1,82E-006	1,29E-008 (0,7050253 %)
	CPE Alpha 1	0,8685528	0,0007710 (0,0887663 %)

RelaxIS 3.0.20.16 - Report

Datasource: 30C_3.0_1

Circuit: I-(R)(C)-P

Type	Value
**Temperature:**	30,000000
**Free variable:**	0,0
**DC Voltage:**	N/A
**AC Voltage:**	N/A
**Time:**	46,085674
**Harmonic:**	N/A
**Free Variable 2:**	N/A
**Area:**	N/A
**Thickness:**	N/A

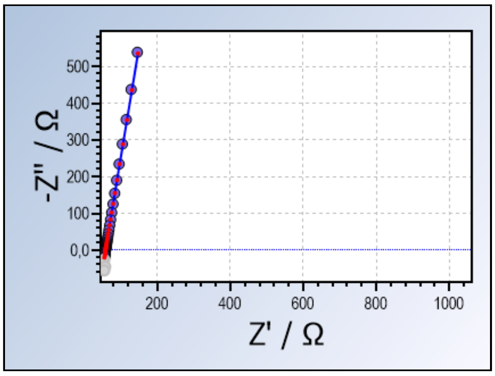

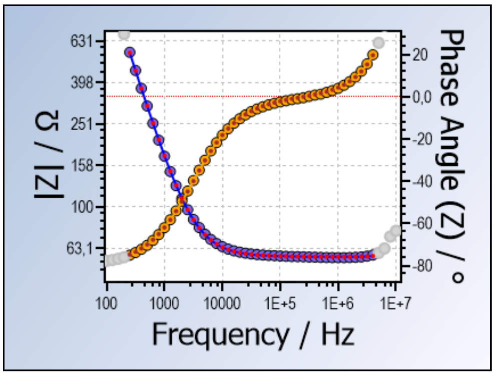

Fit Parameters:

Fix?	Name	Value	Error (Relative)
	Inductance 1	1,29E-006	2,27E-008 (1,7671797 %)
	Resistance 1	57,365955	0,0619682 (0,1080226 %)
	Capacitance 1	1,60E-010	7,07E-012 (4,4239271 %)
	CPE Q 1	2,53E-006	2,03E-008 (0,8009807 %)
	CPE Alpha 1	0,8949093	0,0009050 (0,1011292 %)

RelaxIS 3.0.20.16 - Report

Datasource: 40C_2.5_1

Circuit: I-(R)(C)-P

Type	Value
**Temperature:**	40,000000
**Free variable:**	0,5000000
**DC Voltage:**	N/A
**AC Voltage:**	N/A
**Time:**	46,347910
**Harmonic:**	N/A
**Free Variable 2:**	N/A
**Area:**	N/A

**Thickness:**	N/A

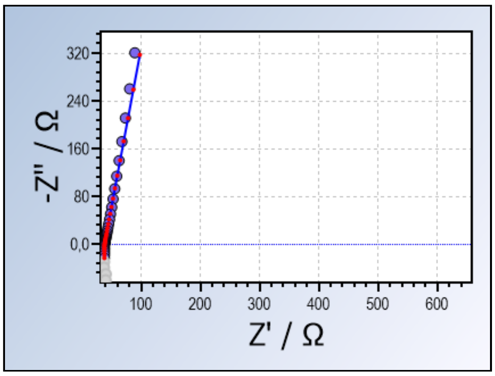

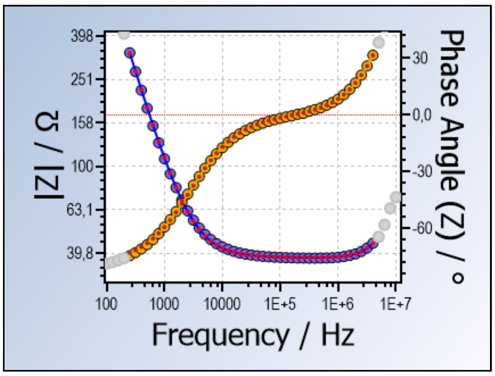

Fit Parameters:

Fix?	Name	Value	Error (Relative)
	Inductance 1	1,03E-006	7,93E-008 (7,6691190 %)
	Resistance 1	37,899483	0,0638533 (0,1684805 %)
	Capacitance 1	7,63E-011	5,53E-011 (72,486119 %)
	CPE Q 1	4,68E-006	5,96E-008 (1,2731047 %)
	CPE Alpha 1	0,8816664	0,0014461 (0,1640236 %)

RelaxIS 3.0.20.16 - Report

Datasource: 40C_2.6_1

Circuit: I-(R)(C)-P

Type	Value
**Temperature:**	40,000000
**Free variable:**	0,4000000
**DC Voltage:**	N/A
**AC Voltage:**	N/A
**Time:**	45,967963
**Harmonic:**	N/A
**Free Variable 2:**	N/A
**Area:**	N/A
**Thickness:**	N/A

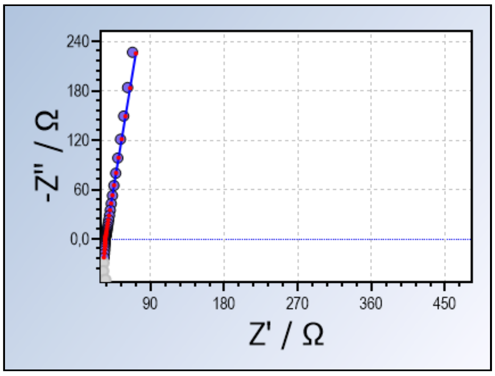

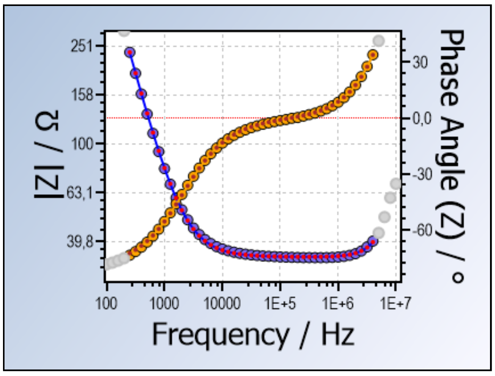

Fit Parameters:

Fix?	Name	Value	Error (Relative)
	Inductance 1	1,17E-006	2,17E-008 (1,8522538 %)
	Resistance 1	34,420283	0,0501915 (0,1458196 %)
	Capacitance 1	2,52E-010	1,87E-011 (7,4144160 %)
	CPE Q 1	6,01E-006	7,29E-008 (1,2146268 %)
	CPE Alpha 1	0,8950259	0,0013998 (0,1563955 %)

RelaxIS 3.0.20.16 - Report

Datasource: 40C_2.7_1

Circuit: I-(R)(C)-P

Type	Value
**Temperature:**	40,000000
**Free variable:**	0,3000000
**DC Voltage:**	N/A
**AC Voltage:**	N/A
**Time:**	46,168176
**Harmonic:**	N/A
**Free Variable 2:**	N/A
**Area:**	N/A

**Thickness:**	N/A

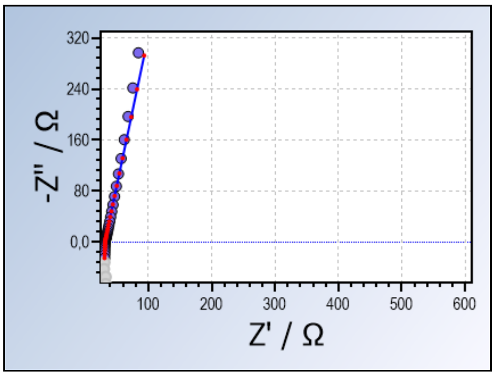

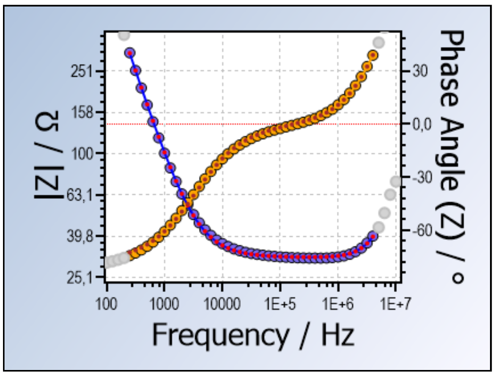

Fit Parameters:

Fix?	Name	Value	Error (Relative)
	Inductance 1	1,17E-006	5,08E-008 (4,3343092 %)
	Resistance 1	31,380855	0,0749389 (0,2388044 %)
	Capacitance 1	1,78E-010	5,20E-011 (29,257801 %)
	CPE Q 1	5,64E-006	9,65E-008 (1,7103917 %)
	CPE Alpha 1	0,8670406	0,0019287 (0,2224435 %)

RelaxIS 3.0.20.16 - Report

Datasource: 40C_2.8_1

Circuit: I-(R)(C)-P

Type	Value
**Temperature:**	40,000000
**Free variable:**	0,2000000
**DC Voltage:**	N/A
**AC Voltage:**	N/A
**Time:**	46,458004
**Harmonic:**	N/A
**Free Variable 2:**	N/A
**Area:**	N/A
**Thickness:**	N/A

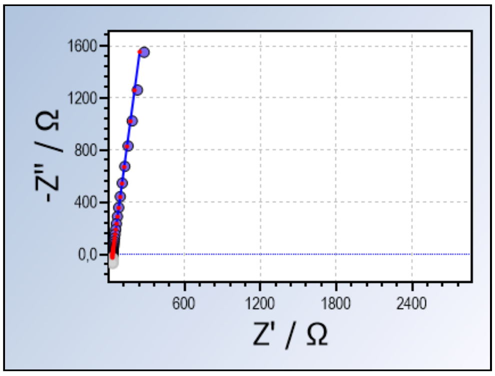

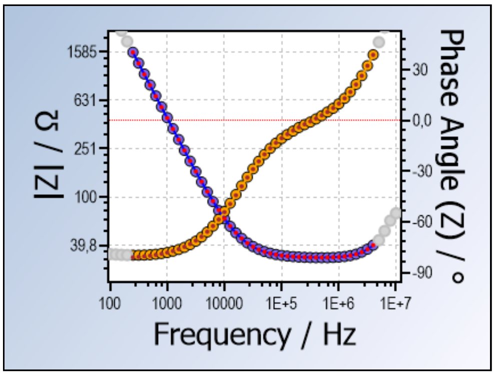

Fit Parameters:

Fix?	Name	Value	Error (Relative)
	Inductance 1	1,12E-006	3,96E-008 (3,5204950 %)
	Resistance 1	31,334641	0,0436912 (0,1394341 %)
	Capacitance 1	1,19E-010	4,05E-011 (34,012102 %)
	CPE Q 1	7,69E-007	4,89E-009 (0,6351030 %)
	CPE Alpha 1	0,9124039	0,0006550 (0,0717918 %)

RelaxIS 3.0.20.16 - Report

Datasource: 40C_2.9_1

Circuit: I-(R)(C)-P

Type	Value
**Temperature:**	40,000000
**Free variable:**	0,1000000
**DC Voltage:**	N/A
**AC Voltage:**	N/A
**Time:**	46,611480
**Harmonic:**	N/A
**Free Variable 2:**	N/A
**Area:**	N/A

**Thickness:**	N/A

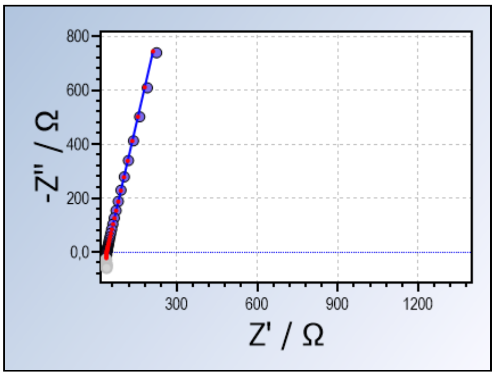

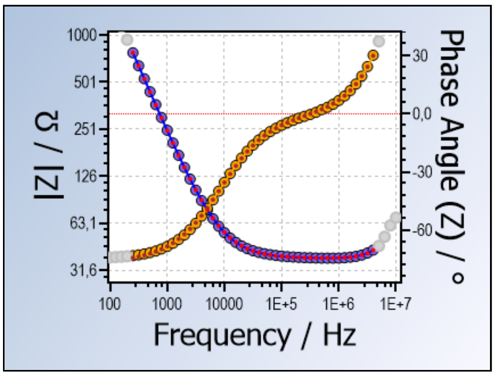

Fit Parameters:

Fix?	Name	Value	Error (Relative)
	Inductance 1	1,08E-006	2,21E-008 (2,0397528 %)
	Resistance 1	37,543047	0,0418399 (0,1114450 %)
	Capacitance 1	1,64E-010	1,58E-011 (9,6283275 %)
	CPE Q 1	2,43E-006	1,50E-008 (0,6164824 %)
	CPE Alpha 1	0,8542492	0,0006638 (0,0777092 %)

RelaxIS 3.0.20.16 - Report

Datasource: 40C_3.0_1

Circuit: I-(R)(C)-P

Type	Value
**Temperature:**	40,000000
**Free variable:**	0,0
**DC Voltage:**	N/A
**AC Voltage:**	N/A
**Time:**	46,417170
**Harmonic:**	N/A
**Free Variable 2:**	N/A
**Area:**	N/A
**Thickness:**	N/A

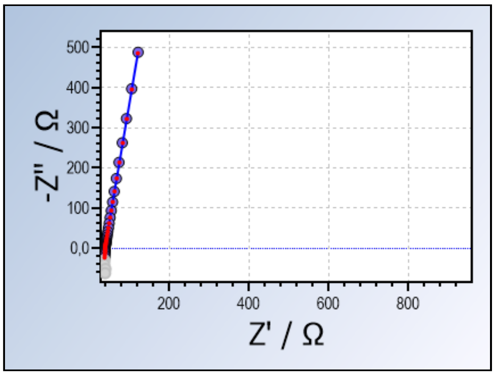

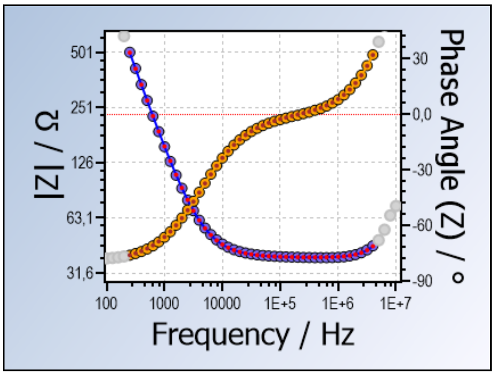

Fit Parameters:

Fix?	Name	Value	Error (Relative)
	Inductance 1	1,18E-006	1,88E-008 (1,5957212 %)
	Resistance 1	38,410978	0,0333380 (0,0867929 %)
	Capacitance 1	1,63E-010	1,29E-011 (7,9018954 %)
	CPE Q 1	2,84E-006	1,65E-008 (0,5802658 %)
	CPE Alpha 1	0,8925741	0,0006446 (0,0722125 %)

RelaxIS 3.0.20.16 - Report

Datasource: 5.5C-2.5.txt_1

Circuit: I-(R)(P)-P

Type	Value
**Temperature:**	5,5000000
**Free variable:**	0,5000000
**DC Voltage:**	N/A
**AC Voltage:**	N/A
**Time:**	46,997648
**Harmonic:**	N/A
**Free Variable 2:**	N/A
**Area:**	N/A

**Thickness:**	N/A

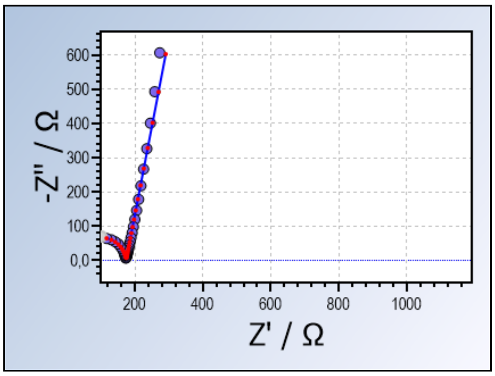

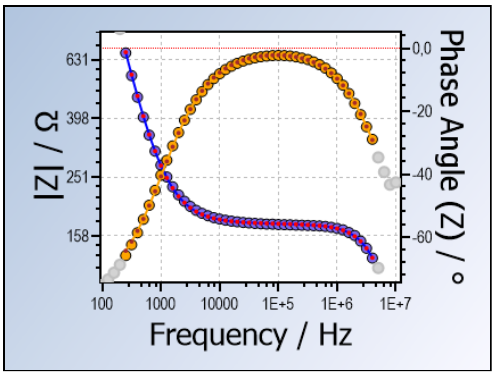

Fit Parameters:

Fix?	Name	Value	Error (Relative)
	Inductance 1	3,65E-007	4,98E-008 (13,643980 %)
	Resistance 1	174,13832	0,2489680 (0,1429714 %)
	CPE Q 1	4,39E-010	5,46E-011 (12,454811 %)
	CPE Alpha 1	0,9391211	0,0078746 (0,8385071 %)
	CPE Q 2	2,53E-006	3,45E-008 (1,3635809 %)
	CPE Alpha 2	0,8782744	0,0016269 (0,1852399 %)

RelaxIS 3.0.20.16 - Report

Datasource: 5.5C-2.6.txt_1

Circuit: I-(R)(P)-P

Type	Value
**Temperature:**	5,5000000
**Free variable:**	0,4000000
**DC Voltage:**	N/A
**AC Voltage:**	N/A
**Time:**	46,566658
**Harmonic:**	N/A
**Free Variable 2:**	N/A
**Area:**	N/A
**Thickness:**	N/A

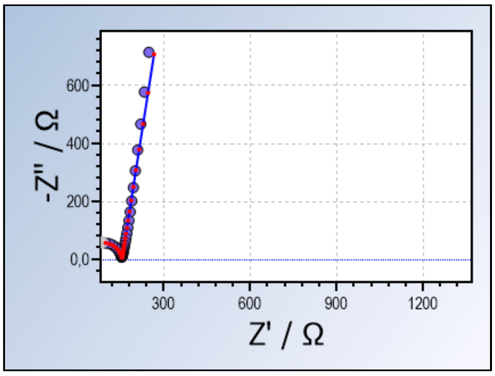

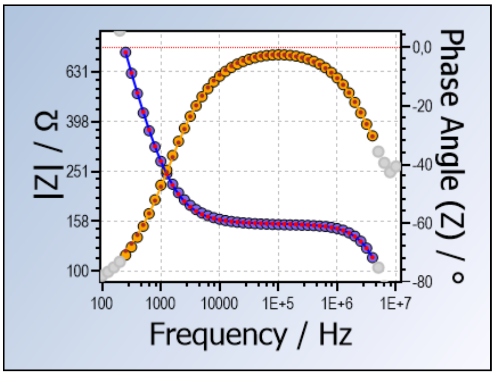

Fit Parameters:

Fix?	Name	Value	Error (Relative)
	Inductance 1	3,67E-007	4,69E-008 (12,785243 %)
	Resistance 1	154,78611	0,2612942 (0,1688099 %)
	CPE Q 1	6,33E-010	8,48E-011 (13,391077 %)
	CPE Alpha 1	0,9294711	0,0084050 (0,9042812 %)
	CPE Q 2	1,84E-006	2,67E-008 (1,4500303 %)
	CPE Alpha 2	0,9006717	0,0017092 (0,1897695 %)

RelaxIS 3.0.20.16 - Report

Datasource: 5.5C-2.7.txt_1

Circuit: I-(R)(P)-P

Type	Value
**Temperature:**	5,5000000
**Free variable:**	0,3000000
**DC Voltage:**	N/A
**AC Voltage:**	N/A
**Time:**	48,079039
**Harmonic:**	N/A
**Free Variable**	N/A

**2:**	
**Area:**	N/A
**Thickness:**	N/A

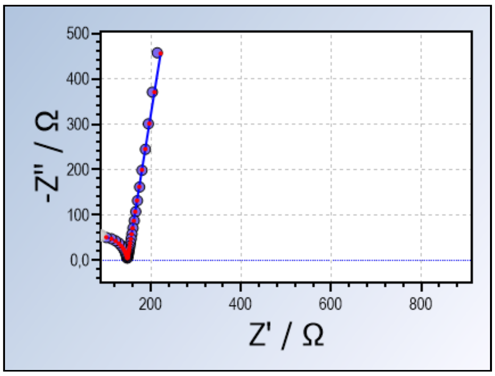

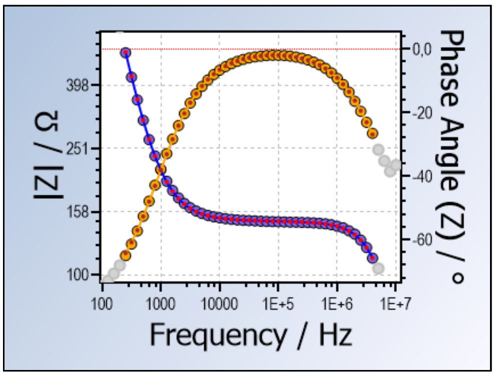

Fit Parameters:

Fix?	Name	Value	Error (Relative)
	Inductance 1	4,21E-007	2,38E-008 (5,6510426 %)
	Resistance 1	147,80115	0,1138522 (0,0770306 %)
	CPE Q 1	5,51E-010	3,89E-011 (7,0639802 %)
	CPE Alpha 1	0,9333977	0,0044767 (0,4796159 %)
	CPE Q 2	2,91E-006	2,28E-008 (0,7851064 %)
	CPE Alpha 2	0,8979384	0,0009423 (0,1049413 %)

RelaxIS 3.0.20.16 - Report

Datasource: 5.5C-2.8.txt_1

Circuit: I-(R)(P)-P

Type	Value
**Temperature:**	5,5000000
**Free variable:**	0,2000000
**DC Voltage:**	N/A
**AC Voltage:**	N/A
**Time:**	48,793091
**Harmonic:**	N/A
**Free Variable 2:**	N/A
**Area:**	N/A
**Thickness:**	N/A

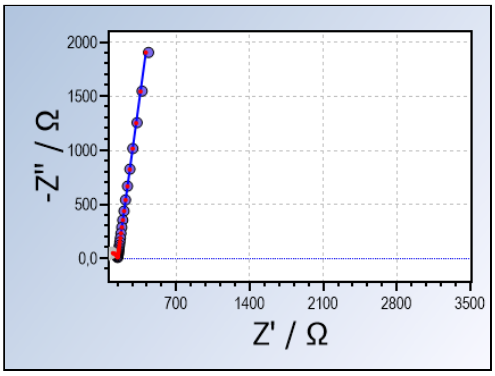

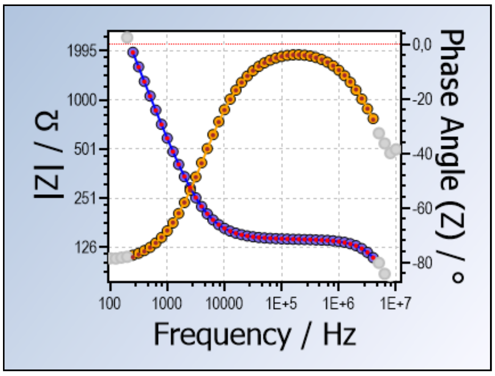

Fit Parameters:

Fix?	Name	Value	Error (Relative)
	Inductance 1	3,75E-007	1,89E-008 (5,0315300 %)
	Resistance 1	140,75398	0,0994573 (0,0706604 %)
	CPE Q 1	4,92E-010	2,92E-011 (5,9217571 %)
	CPE Alpha 1	0,9428162	0,0037500 (0,3977467 %)
	CPE Q 2	6,36E-007	2,65E-009 (0,4175967 %)
	CPE Alpha 2	0,9105914	0,0004647 (0,0510370 %)

RelaxIS 3.0.20.16 - Report

Datasource: 5.5C-2.9.txt_1

Circuit: I-(R)(P)-P

Type	Value
**Temperature:**	5,5000000
**Free variable:**	0,1000000
**DC Voltage:**	N/A
**AC Voltage:**	N/A
**Time:**	46,359560
**Harmonic:**	N/A
**Free Variable**	N/A

**2:**	
**Area:**	N/A
**Thickness:**	N/A

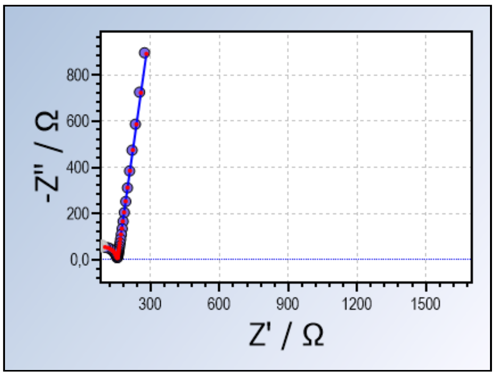

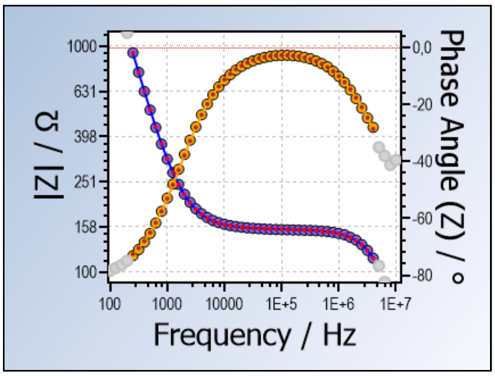

Fit Parameters:

Fix?	Name	Value	Error (Relative)
	Inductance 1	3,69E-007	2,79E-008 (7,5582102 %)
	Resistance 1	154,91822	0,1507429 (0,0973048 %)
	CPE Q 1	7,40E-010	6,01E-011 (8,1182381 %)
	CPE Alpha 1	0,9169615	0,0051127 (0,5575710 %)
	CPE Q 2	1,36E-006	1,04E-008 (0,7649625 %)
	CPE Alpha 2	0,9102857	0,0008916 (0,0979428 %)

RelaxIS 3.0.20.16 - Report

Datasource: 5.5C-3.0.txt_1

Circuit: I-(R)(P)-P

Type	Value
**Temperature:**	5,5000000
**Free variable:**	0,0
**DC Voltage:**	N/A
**AC Voltage:**	N/A
**Time:**	49,782693
**Harmonic:**	N/A
**Free Variable 2:**	N/A
**Area:**	N/A
**Thickness:**	N/A

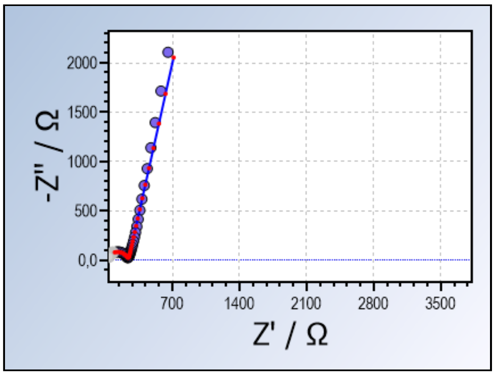

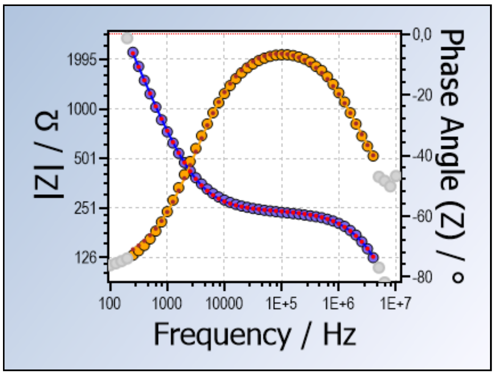

Fit Parameters:

Fix?	Name	Value	Error (Relative)
	Inductance 1	1,98E-007	7,14E-008 (36,079494 %)
	Resistance 1	240,89473	0,9517032 (0,3950702 %)
	CPE Q 1	5,97E-009	9,00E-010 (15,067652 %)
	CPE Alpha 1	0,8043790	0,0091631 (1,1391506 %)
	CPE Q 2	8,63E-007	1,96E-008 (2,2646418 %)
	CPE Alpha 2	0,8566549	0,0025900 (0,3023430 %)

RelaxIS 3.0.20.16 - Report

Datasource: 24.1C-2.5.txt_1

Circuit: I-(R)(P)-P

Type	Value
**Temperature:**	24,400000
**Free variable:**	0,5000000
**DC Voltage:**	N/A
**AC Voltage:**	N/A
**Time:**	46,225245
**Harmonic:**	N/A
**Free Variable**	N/A

2:	
Area:	N/A
Thickness:	N/A

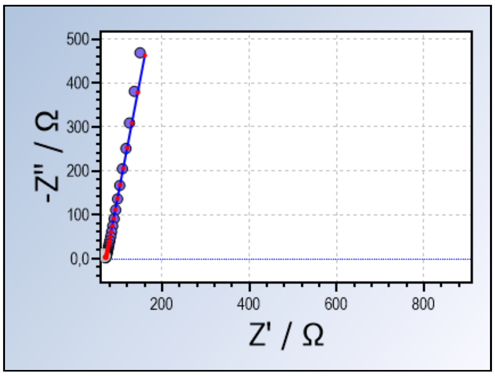

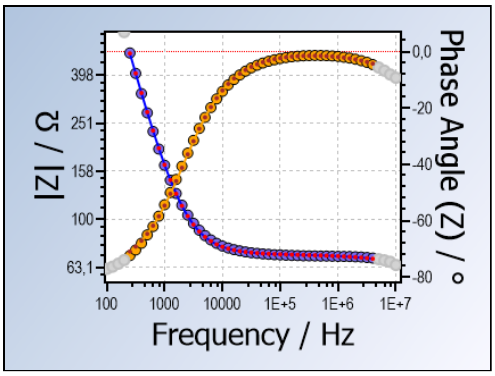

Fit Parameters:

Fix?	Name	Value	Error (Relative)
	Inductance 1	1,00E-015	1,54E-008 (1,54E+009 %)
	Resistance 1	71,295237	0,1429274 (0,2004726 %)
	CPE Q 1	6,66E-009	4,53E-009 (68,017968 %)
	CPE Alpha 1	0,7089443	0,0412024 (5,8117960 %)
	CPE Q 2	3,28E-006	4,27E-008 (1,3018989 %)
	CPE Alpha 2	0,8788200	0,0015105 (0,1718759 %)

RelaxIS 3.0.20.16 - Report

Datasource: 24.1C-2.6.txt_1

Circuit: I-(R)(P)-P

Type	Value
Temperature:	24,400000
Free variable:	0,4000000
DC Voltage:	N/A
AC Voltage:	N/A
Time:	46,219715
Harmonic:	N/A
Free Variable 2:	N/A
Area:	N/A
Thickness:	N/A

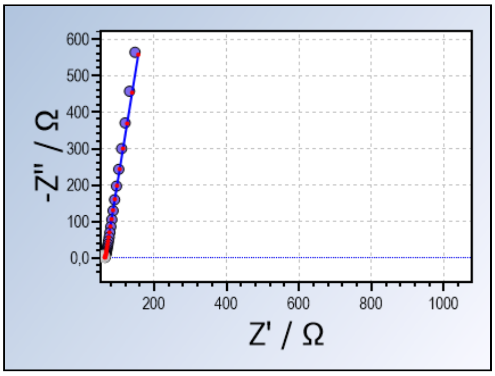

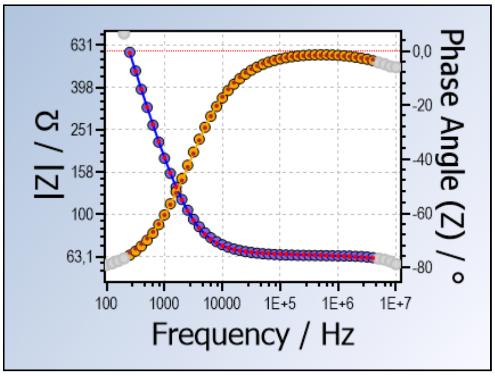

Fit Parameters:

Fix?	Name	Value	Error (Relative)
	Inductance 1	1,00E-015	1,81E-008 (1,81E+009 %)
	Resistance 1	64,826002	0,1844264 (0,2844945 %)
	CPE Q 1	5,49E-008	6,56E-008 (119,55468 %)
	CPE Alpha 1	0,5785641	0,0737523 (12,747468 %)
	CPE Q 2	2,39E-006	3,19E-008 (1,3309756 %)
	CPE Alpha 2	0,8968802	0,0015361 (0,1712733 %)

RelaxIS 3.0.20.16 - Report

Datasource: 24.1C-2.7.txt_1

Circuit: I-(R)(P)-P

Type	Value
Temperature:	24,400000
Free variable:	0,3000000
DC Voltage:	N/A
AC Voltage:	N/A
Time:	46,060927
Harmonic:	N/A
Free Variable	N/A

**2:**	
**Area:**	N/A
**Thickness:**	N/A

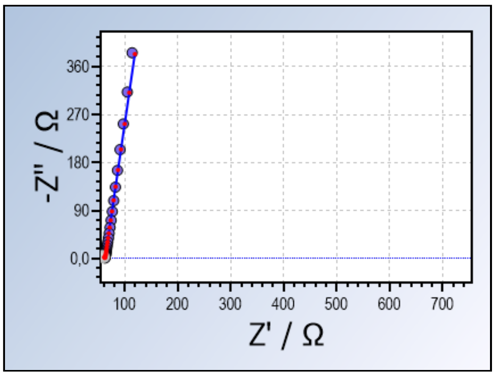

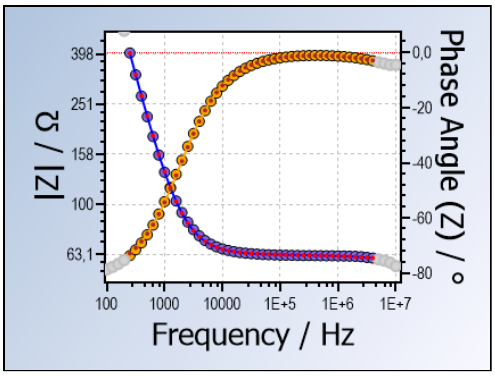

Fit Parameters:

Fix?	Name	Value	Error (Relative)
	Inductance 1	1,00E-015	8,94E-009 (8,94E+008 %)
	Resistance 1	62,880934	0,0777522 (0,1236499 %)
	CPE Q 1	1,04E-008	6,87E-009 (66,267297 %)
	CPE Alpha 1	0,6674171	0,0411665 (6,1680305 %)
	CPE Q 2	3,23E-006	2,61E-008 (0,8082531 %)
	CPE Alpha 2	0,9074794	0,0009436 (0,1039854 %)

RelaxIS 3.0.20.16 - Report

Datasource: 24.1C-2.8.txt_1

Circuit: I-(R)(P)-P

Type	Value
**Temperature:**	24,400000
**Free variable:**	0,2000000
**DC Voltage:**	N/A
**AC Voltage:**	N/A
**Time:**	46,165715
**Harmonic:**	N/A
**Free Variable 2:**	N/A
**Area:**	N/A
**Thickness:**	N/A

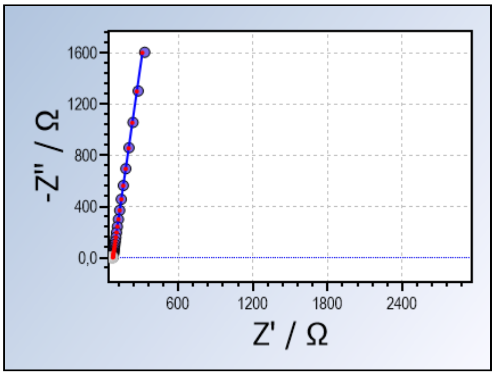

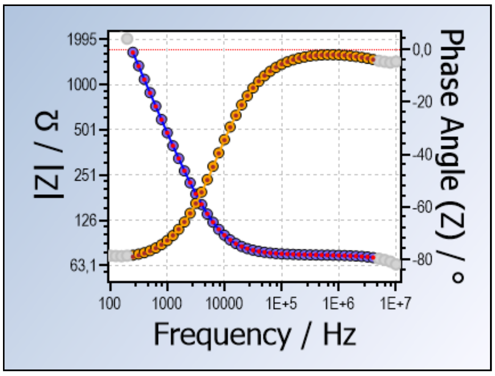

Fit Parameters:

Fix?	Name	Value	Error (Relative)
	Inductance 1	1,60E-007	4,15E-008 (25,970847 %)
	Resistance 1	74,291555	0,0556087 (0,0748520 %)
	CPE Q 1	8,56E-010	3,45E-010 (40,314943 %)
	CPE Alpha 1	0,8513287	0,0299101 (3,5133473 %)
	CPE Q 2	7,79E-007	2,68E-009 (0,3441697 %)
	CPE Alpha 2	0,9065378	0,0003747 (0,0413277 %)

RelaxIS 3.0.20.16 - Report

Datasource: 24.1C-2.9.txt_1

Circuit: I-(R)(P)-P

Type	Value
**Temperature:**	24,400000
**Free variable:**	0,1000000
**DC Voltage:**	N/A
**AC Voltage:**	N/A
**Time:**	46,342880
**Harmonic:**	N/A
**Free Variable**	N/A

**2:**	
**Area:**	N/A
**Thickness:**	N/A

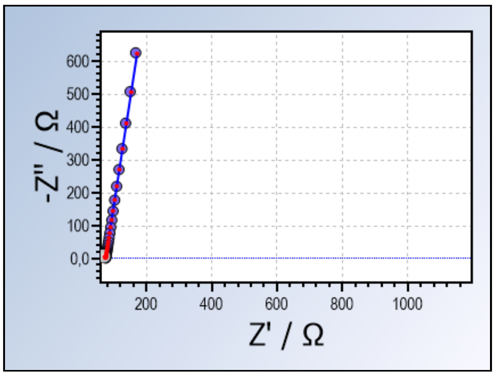

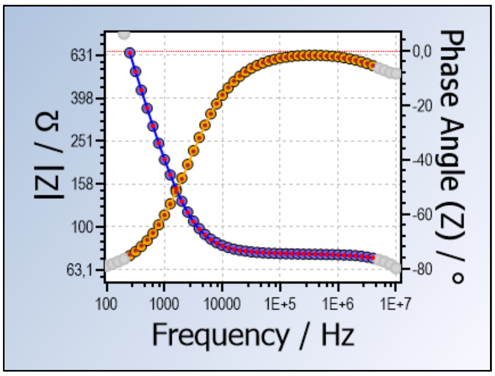

Fit Parameters:

Fix?	Name	Value	Error (Relative)
	Inductance 1	2,07E-008	4,71E-008 (226,92809 %)
	Resistance 1	75,103975	0,0737709 (0,0982251 %)
	CPE Q 1	3,20E-009	2,12E-009 (66,350376 %)
	CPE Alpha 1	0,7641618	0,0468712 (6,1336687 %)
	CPE Q 2	2,06E-006	1,17E-008 (0,5674122 %)
	CPE Alpha 2	0,9024715	0,0006524 (0,0722937 %)

RelaxIS 3.0.20.16 - Report

Datasource: 24.1C-3.0.txt_1

Circuit: I-(R)(P)-P

Type	Value
**Temperature:**	24,400000
**Free variable:**	0,0
**DC Voltage:**	N/A
**AC Voltage:**	N/A
**Time:**	46,249859
**Harmonic:**	N/A
**Free Variable 2:**	N/A
**Area:**	N/A
**Thickness:**	N/A

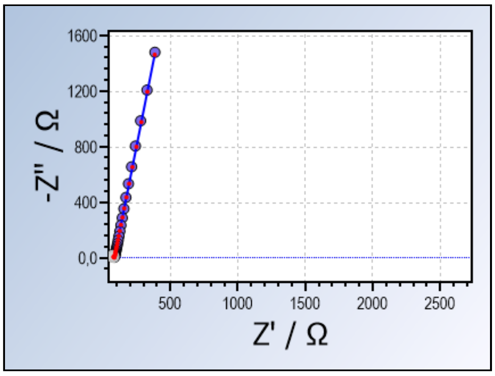

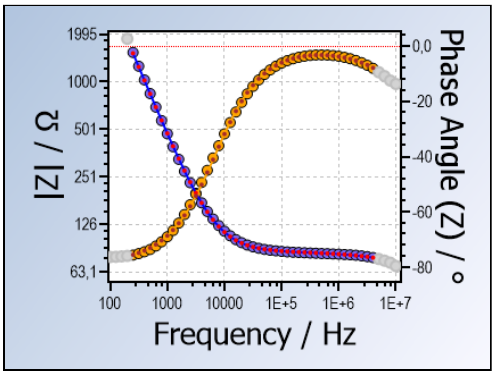

Fit Parameters:

Fix?	Name	Value	Error (Relative)
	Inductance 1	1,00E-015	8,38E-009 (8,38E+008 %)
	Resistance 1	83,720064	0,1783436 (0,2130237 %)
	CPE Q 1	1,19E-008	3,85E-009 (32,419692 %)
	CPE Alpha 1	0,7037244	0,0191772 (2,7251038 %)
	CPE Q 2	1,09E-006	1,01E-008 (0,9323310 %)
	CPE Alpha 2	0,8718125	0,0010248 (0,1175444 %)

RelaxIS 3.0.20.16 - Report

Datasource: -0.6_3.0_1

Circuit: I-(R)(P)-P

Type	Value
**Temperature:**	-0,600000
**Free variable:**	0,0
**DC Voltage:**	N/A
**AC Voltage:**	N/A
**Time:**	46,748366
**Harmonic:**	N/A
**Free Variable**	N/A

**2:**	
**Area:**	N/A
**Thickness:**	N/A

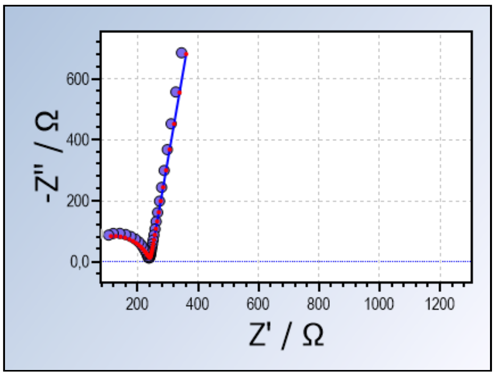

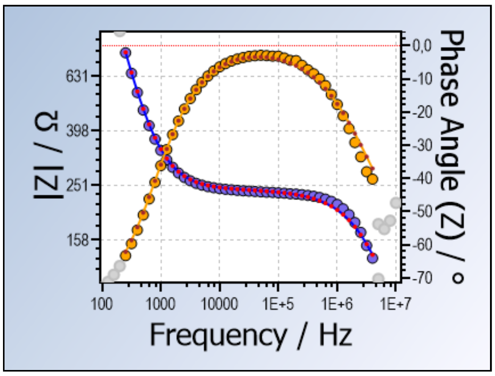

Fit Parameters:

Fix?	Name	Value	Error (Relative)
	Inductance 1	1,00E-015	5,13E-007 (5,13E+010 %)
	Resistance 1	241,80371	1,3052968 (0,5398167 %)
	CPE Q 1	8,23E-009	1,51E-009 (18,281902 %)
	CPE Alpha 1	0,7766212	0,0110124 (1,4179919 %)
	CPE Q 2	2,05E-006	1,07E-007 (5,1983701 %)
	CPE Alpha 2	0,8903343	0,0062782 (0,7051518 %)

RelaxIS 3.0.20.16 - Report

Datasource: -0.6C_2.5_1

Circuit: I-(R)(P)-P

Type	Value
**Temperature:**	-0,600000
**Free variable:**	0,5000000
**DC Voltage:**	N/A
**AC Voltage:**	N/A
**Time:**	46,176039
**Harmonic:**	N/A
**Free Variable 2:**	N/A
**Area:**	N/A
**Thickness:**	N/A

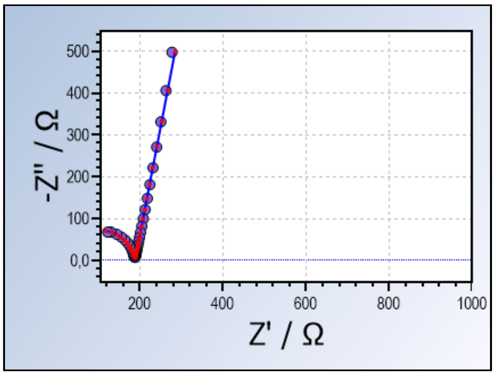

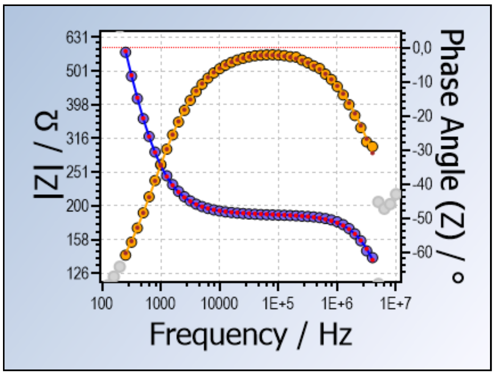

Fit Parameters:

Fix?	Name	Value	Error (Relative)
	Inductance 1	2,06E-007	4,75E-008 (23,128435 %)
	Resistance 1	188,27540	0,2684620 (0,1425900 %)
	CPE Q 1	1,02E-009	1,18E-010 (11,577141 %)
	CPE Alpha 1	0,8899234	0,0072642 (0,8162765 %)
	CPE Q 2	3,05E-006	4,58E-008 (1,5017142 %)
	CPE Alpha 2	0,8786996	0,0018150 (0,2065583 %)

RelaxIS 3.0.20.16 - Report

Datasource: -0.6C_2.6_1

Circuit: I-(R)(P)-P

Type	Value
**Temperature:**	-0,600000
**Free variable:**	0,4000000
**DC Voltage:**	N/A
**AC Voltage:**	N/A
**Time:**	46,176184
**Harmonic:**	N/A
**Free Variable**	N/A

**2:**	
**Area:**	N/A
**Thickness:**	N/A

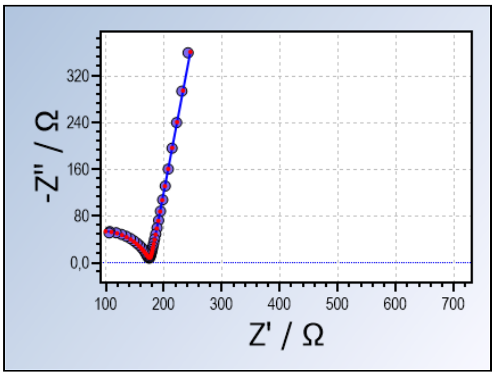

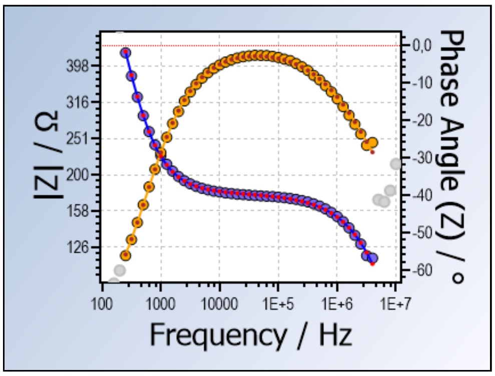

Fit Parameters:

Fix?	Name	Value	Error (Relative)
	Inductance 1	1,02E-007	4,73E-008 (46,417010 %)
	Resistance 1	176,90427	0,4025224 (0,2275368 %)
	CPE Q 1	1,88E-008	2,66E-009 (14,126941 %)
	CPE Alpha 1	0,7299693	0,0087436 (1,1977983 %)
	CPE Q 2	4,17E-006	1,01E-007 (2,4185780 %)
	CPE Alpha 2	0,8800695	0,0029718 (0,3376771 %)

RelaxIS 3.0.20.16 - Report

Datasource: -0.6C_2.7_1

Circuit: I-(R)(P)-P

Type	Value
**Temperature:**	-0,600000
**Free variable:**	0,3000000
**DC Voltage:**	N/A
**AC Voltage:**	N/A
**Time:**	46,256760
**Harmonic:**	N/A
**Free Variable 2:**	N/A
**Area:**	N/A
**Thickness:**	N/A

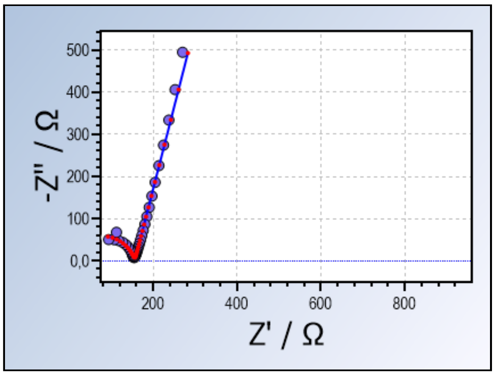

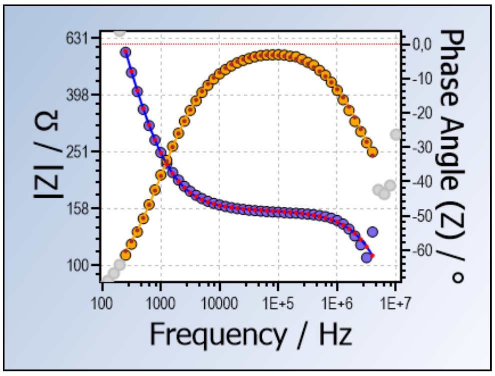

Fit Parameters:

Fix?	Name	Value	Error (Relative)
	Inductance 1	1,00E-015	4,09E-008 (4,09E+009 %)
	Resistance 1	155,76285	0,9486415 (0,6090294 %)
	CPE Q 1	3,94E-009	1,09E-009 (27,570947 %)
	CPE Alpha 1	0,8263724	0,0167783 (2,0303507 %)
	CPE Q 2	4,05E-006	2,23E-007 (5,5012682 %)
	CPE Alpha 2	0,8396786	0,0065930 (0,7851763 %)

RelaxIS 3.0.20.16 - Report

Datasource: -0.6C_2.8_1

Circuit: I-(R)(P)-P

Type	Value
**Temperature:**	-0,600000
**Free variable:**	0,2000000
**DC Voltage:**	N/A
**AC Voltage:**	N/A
**Time:**	46,227117
**Harmonic:**	N/A
**Free Variable**	N/A

**2:**	
**Area:**	N/A
**Thickness:**	N/A

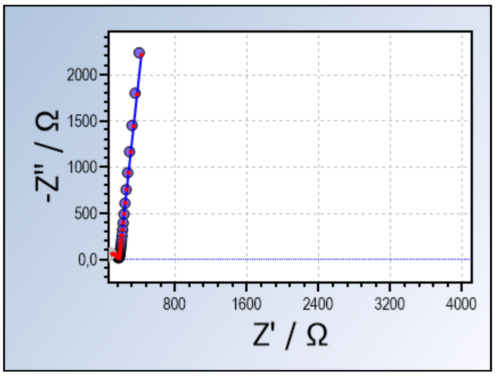

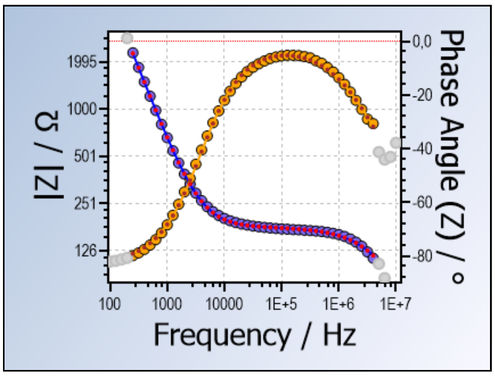

Fit Parameters:

Fix?	Name	Value	Error (Relative)
	Inductance 1	1,02E-015	0,0 (0,0 %)
	Resistance 1	176,68958	0,5435396 (0,3076240 %)
	CPE Q 1	8,08E-009	9,57E-010 (11,850300 %)
	CPE Alpha 1	0,7800831	0,0071070 (0,9110626 %)
	CPE Q 2	4,80E-007	8,24E-009 (1,7185936 %)
	CPE Alpha 2	0,9289853	0,0019298 (0,2077344 %)

RelaxIS 3.0.20.16 - Report

Datasource: -0.6C_2.9_1

Circuit: I-(R)(P)-P

Type	Value
**Temperature:**	-0,600000
**Free variable:**	0,1000000
**DC Voltage:**	N/A
**AC Voltage:**	N/A
**Time:**	45,917583
**Harmonic:**	N/A
**Free Variable 2:**	N/A
**Area:**	N/A
**Thickness:**	N/A

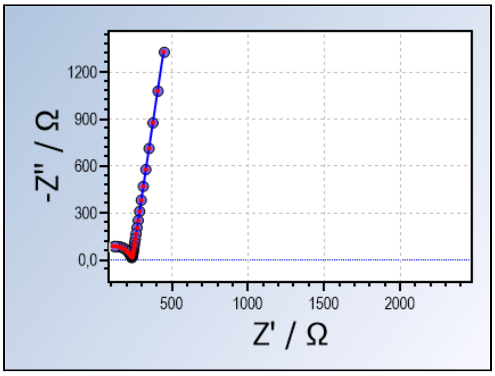

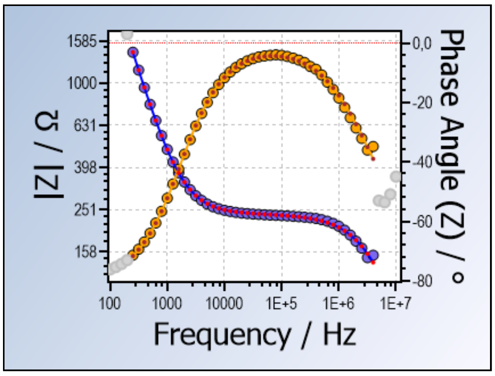

Fit Parameters:

Fix?	Name	Value	Error (Relative)
	Inductance 1	1,08E-015	0,0 (0,0 %)
	Resistance 1	239,02485	0,8937769 (0,3739263 %)
	CPE Q 1	4,40E-009	5,54E-010 (12,589645 %)
	CPE Alpha 1	0,8140416	0,0075962 (0,9331465 %)
	CPE Q 2	9,64E-007	2,68E-008 (2,7829441 %)
	CPE Alpha 2	0,9026835	0,0032526 (0,3603283 %)

RelaxIS 3.0.20.16 - Report

Datasource: -9.4C_2.5_1

Circuit: I-(R)(P)-P

Type	Value
**Temperature:**	-9,400000
**Free variable:**	0,5000000
**DC Voltage:**	N/A
**AC Voltage:**	N/A
**Time:**	46,097719
**Harmonic:**	N/A
**Free Variable**	N/A

**2:**	
**Area:**	N/A
**Thickness:**	N/A

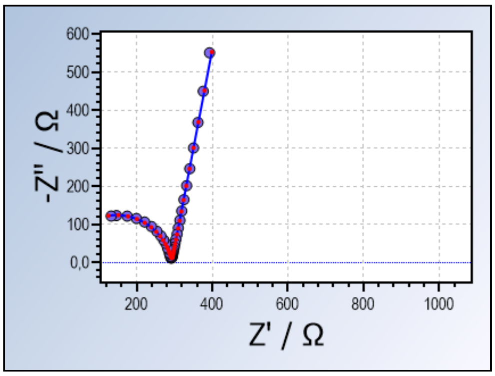

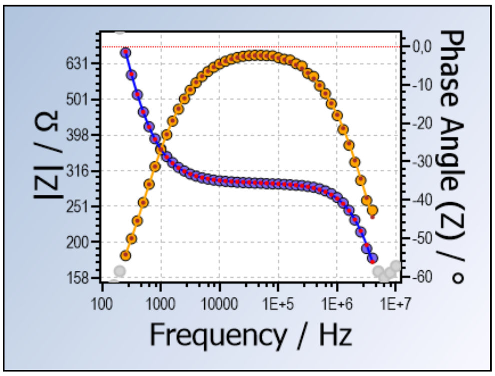

Fit Parameters:

Fix? Name	Value	Error (Relative)
Inductance 1	1,00E-015	0,0 (0,0 %)
Resistance 1	292,45593	0,4317291 (0,1476219 %)
CPE Q 1	9,13E-010	4,70E-011 (5,1540689 %)
CPE Alpha 1	0,8982904	0,0031124 (0,3464852 %)
CPE Q 2	2,74E-006	5,14E-008 (1,8747731 %)
CPE Alpha 2	0,8791726	0,0022958 (0,2611363 %)

RelaxIS 3.0.20.16 - Report

Datasource: -9.4C_2.6_1

Circuit: I-(R)(P)-P

Type	Value
**Temperature:**	-9,400000
**Free variable:**	0,4000000
**DC Voltage:**	N/A
**AC Voltage:**	N/A
**Time:**	46,297645
**Harmonic:**	N/A
**Free Variable 2:**	N/A
**Area:**	N/A
**Thickness:**	N/A

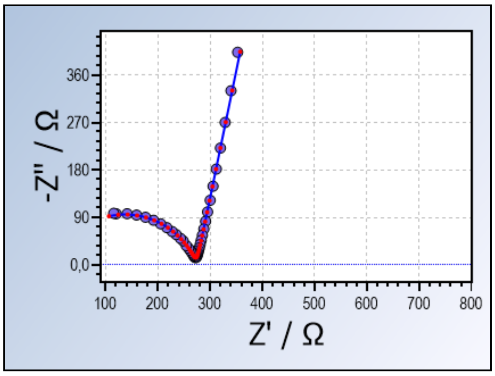

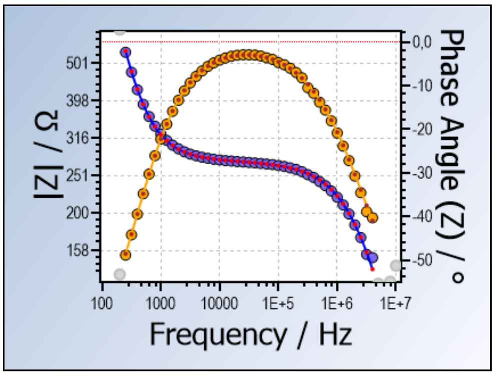

Fit Parameters:

Fix? Name	Value	Error (Relative)
Inductance 1	1,00E-015	0,0 (0,0 %)
Resistance 1	274,00456	0,6289400 (0,2295363 %)
CPE Q 1	8,95E-009	6,08E-010 (6,7957101 %)
CPE Alpha 1	0,7762315	0,0041179 (0,5305023 %)
CPE Q 2	3,98E-006	1,24E-007 (3,1024220 %)
CPE Alpha 2	0,8704399	0,0038453 (0,4417653 %)

RelaxIS 3.0.20.16 - Report

Datasource: -9.4C_2.7_1

Circuit: I-(R)(P)-P

Type	Value
**Temperature:**	-9,400000
**Free variable:**	0,3000000
**DC Voltage:**	N/A
**AC Voltage:**	N/A
**Time:**	46,692992
**Harmonic:**	N/A
**Free Variable**	N/A

2:	
**Area:**	N/A
**Thickness:**	N/A

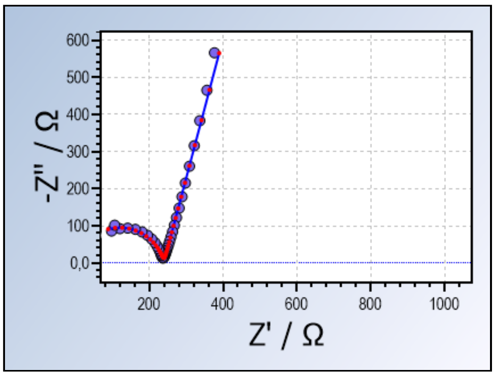

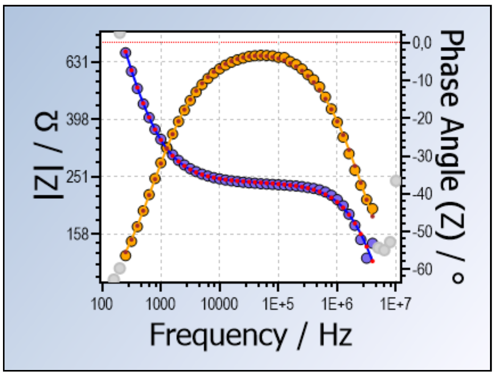

Fit Parameters:

Fix?	Name	Value	Error (Relative)
	Inductance 1	1,00E-015	0,0 (0,0 %)
	Resistance 1	239,22290	1,2501174 (0,5225743 %)
	CPE Q 1	2,59E-009	4,01E-010 (15,469975 %)
	CPE Alpha 1	0,8580417	0,0093656 (1,0915071 %)
	CPE Q 2	3,66E-006	2,01E-007 (5,4984057 %)
	CPE Alpha 2	0,8347487	0,0066739 (0,7995089 %)

RelaxIS 3.0.20.16 - Report

Datasource: -9.4C_2.8_1

Circuit: I-(R)(P)-P

Type	Value
**Temperature:**	-9,400000
**Free variable:**	0,2000000
**DC Voltage:**	N/A
**AC Voltage:**	N/A
**Time:**	47,345180
**Harmonic:**	N/A
**Free Variable 2:**	N/A
**Area:**	N/A
**Thickness:**	N/A

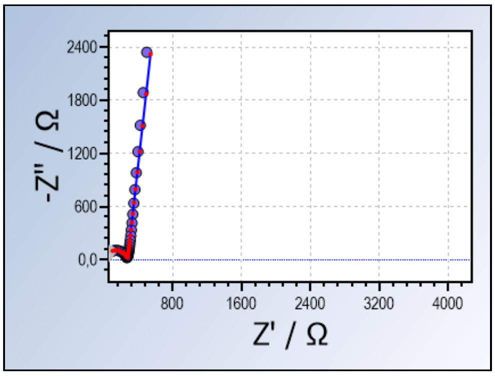

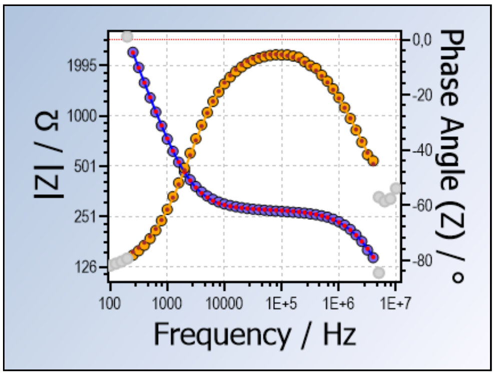

Fit Parameters:

Fix?	Name	Value	Error (Relative)
	Inductance 1	1,27E-015	2,84E-008 (2,24E+009 %)
	Resistance 1	276,18590	0,7390413 (0,2675884 %)
	CPE Q 1	3,34E-009	2,70E-010 (8,0814318 %)
	CPE Alpha 1	0,8354246	0,0048844 (0,5846587 %)
	CPE Q 2	4,64E-007	8,09E-009 (1,7429452 %)
	CPE Alpha 2	0,9271409	0,0019988 (0,2155881 %)

RelaxIS 3.0.20.16 - Report

Datasource: -9.4C_2.9_1

Circuit: I-(R)(P)-P

Type	Value
**Temperature:**	-9,400000
**Free variable:**	0,1000000
**DC Voltage:**	N/A
**AC Voltage:**	N/A
**Time:**	47,531299
**Harmonic:**	N/A
**Free Variable**	N/A

**2:**	
**Area:**	N/A
**Thickness:**	N/A

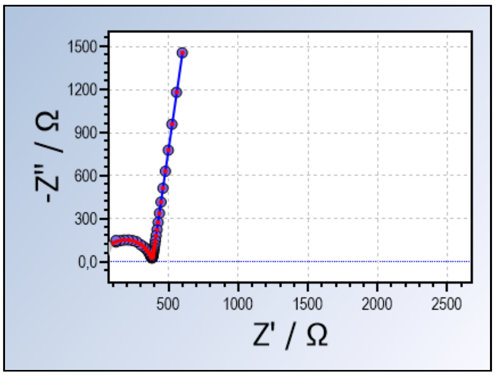

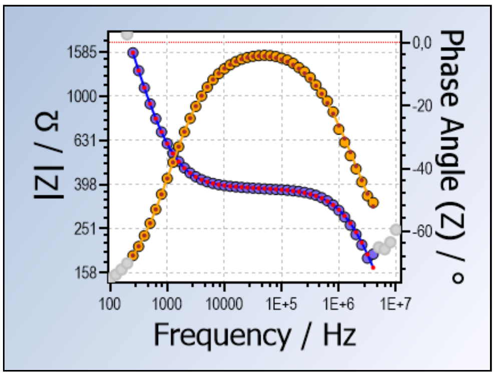

Fit Parameters:

Fix?	Name	Value	Error (Relative)
	Inductance 1	1,03E-015	4,95E-008 (4,79E+009 %)
	Resistance 1	383,61074	1,6451557 (0,4288607 %)
	CPE Q 1	2,22E-009	2,46E-010 (11,122863 %)
	CPE Alpha 1	0,8568571	0,0068511 (0,7995567 %)
	CPE Q 2	8,57E-007	3,21E-008 (3,7401568 %)
	CPE Alpha 2	0,9063191	0,0044564 (0,4917003 %)

RelaxIS 3.0.20.16 - Report

Datasource: -9.4C_3.0_1

Circuit: I-(R)(P)-P

Type	Value
**Temperature:**	-9,400000
**Free variable:**	0,0
**DC Voltage:**	N/A
**AC Voltage:**	N/A
**Time:**	46,651920
**Harmonic:**	N/A
**Free Variable 2:**	N/A
**Area:**	N/A
**Thickness:**	N/A

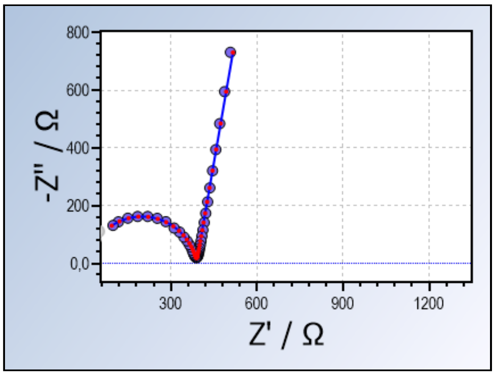

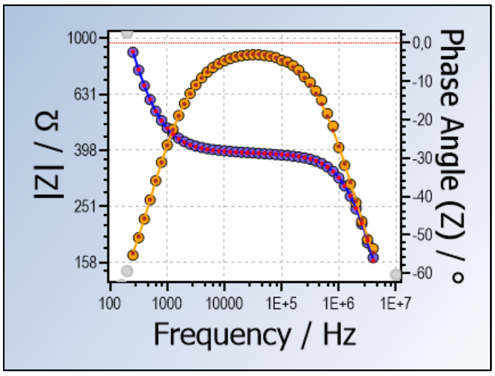

Fit Parameters:

Fix?	Name	Value	Error (Relative)
	Inductance 1	2,74E-007	4,30E-008 (15,685817 %)
	Resistance 1	388,98719	0,6185480 (0,1590150 %)
	CPE Q 1	1,10E-009	5,45E-011 (4,9455400 %)
	CPE Alpha 1	0,9002115	0,0029777 (0,3307794 %)
	CPE Q 2	1,91E-006	3,82E-008 (2,0014461 %)
	CPE Alpha 2	0,8906429	0,0024549 (0,2756335 %)

RelaxIS 3.0.20.16 - Report

Datasource: 9.5C_2.5_1

Circuit: I-(R)(P)-P

Type	Value
**Temperature:**	9,5000000
**Free variable:**	0,5000000
**DC Voltage:**	N/A
**AC Voltage:**	N/A
**Time:**	46,548714
**Harmonic:**	N/A
**Free Variable**	N/A

**2:**	
**Area:**	N/A
**Thickness:**	N/A

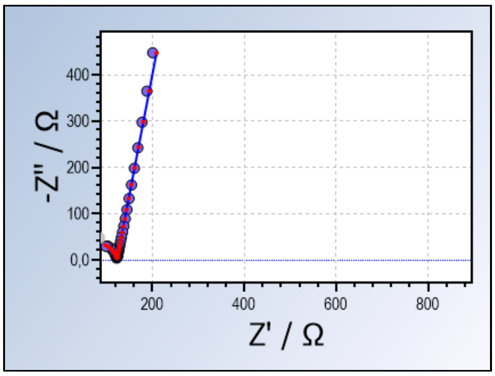

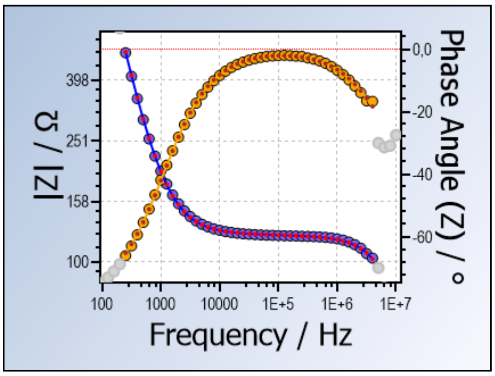

Fit Parameters:

Fix?	Name	Value	Error (Relative)
	Inductance 1	3,12E-007	4,85E-008 (15,557147 %)
	Resistance 1	122,70396	0,1729726 (0,1409674 %)
	CPE Q 1	1,05E-009	2,01E-010 (19,111966 %)
	CPE Alpha 1	0,8842298	0,0124526 (1,4083013 %)
	CPE Q 2	3,39E-006	4,32E-008 (1,2739076 %)
	CPE Alpha 2	0,8789942	0,0015177 (0,1726625 %)

RelaxIS 3.0.20.16 - Report

Datasource: 9.5C_2.6_1

Circuit: I-(R)(P)-P

Type	Value
**Temperature:**	9,5000000
**Free variable:**	0,4000000
**DC Voltage:**	N/A
**AC Voltage:**	N/A
**Time:**	46,291028
**Harmonic:**	N/A
**Free Variable 2:**	N/A
**Area:**	N/A
**Thickness:**	N/A

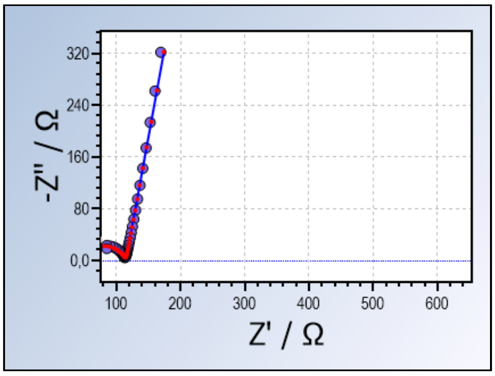

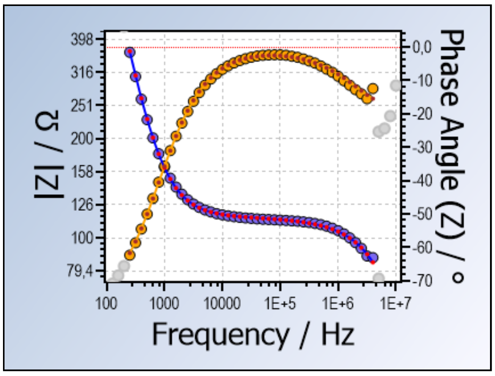

Fit Parameters:

Fix?	Name	Value	Error (Relative)
	Inductance 1	3,86E-007	5,06E-008 (13,122944 %)
	Resistance 1	115,04440	0,2980508 (0,2590746 %)
	CPE Q 1	2,09E-008	5,39E-009 (25,744107 %)
	CPE Alpha 1	0,7223598	0,0162416 (2,2484131 %)
	CPE Q 2	4,52E-006	1,05E-007 (2,3199167 %)
	CPE Alpha 2	0,8847689	0,0028145 (0,3181086 %)

RelaxIS 3.0.20.16 - Report

Datasource: 9.5C_2.7_1

Circuit: I-(R)(P)-P

Type	Value
**Temperature:**	9,5000000
**Free variable:**	0,3000000
**DC Voltage:**	N/A
**AC Voltage:**	N/A
**Time:**	46,240626
**Harmonic:**	N/A
**Free Variable**	N/A

2:	
**Area:**	N/A
**Thickness:**	N/A

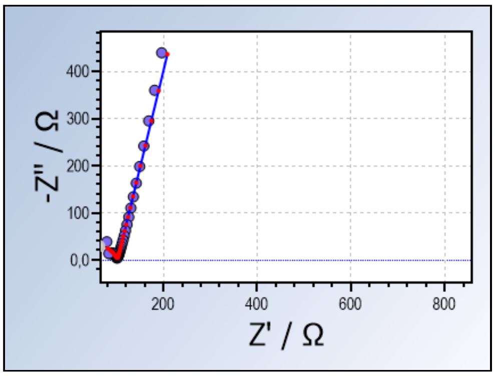

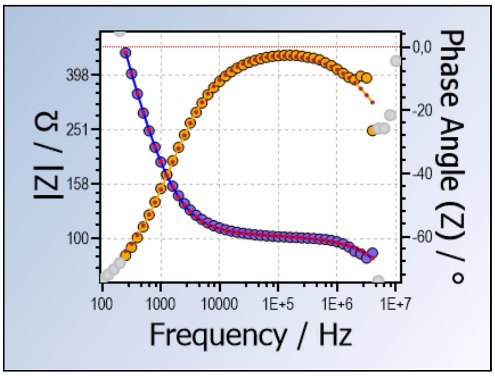

Fit Parameters:

Fix?	Name	Value	Error (Relative)
	Inductance 1	1,00E-015	1,14E-008 (1,14E+009 %)
	Resistance 1	102,24951	0,6699106 (0,6551724 %)
	CPE Q 1	1,18E-008	6,35E-009 (53,990968 %)
	CPE Alpha 1	0,7453302	0,0328571 (4,4083992 %)
	CPE Q 2	4,32E-006	2,15E-007 (4,9761717 %)
	CPE Alpha 2	0,8479590	0,0058907 (0,6946893 %)

RelaxIS 3.0.20.16 - Report

Datasource: 9.5C_2.8_1

Circuit: I-(R)(P)-P

Type	Value
**Temperature:**	9,5000000
**Free variable:**	0,2000000
**DC Voltage:**	N/A
**AC Voltage:**	N/A
**Time:**	46,772638
**Harmonic:**	N/A
**Free Variable 2:**	N/A
**Area:**	N/A
**Thickness:**	N/A

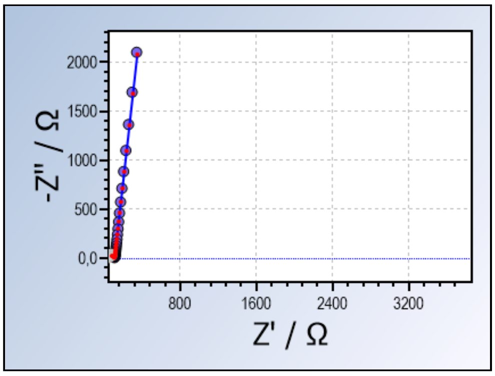

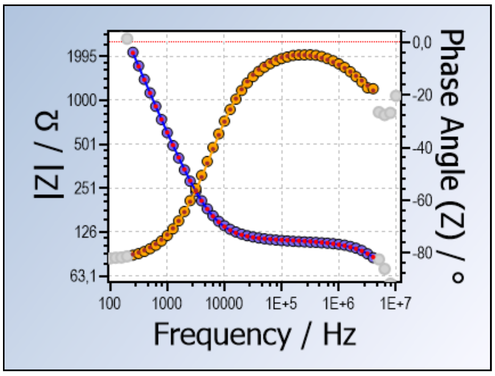

Fit Parameters:

Fix?	Name	Value	Error (Relative)
	Inductance 1	2,66E-007	5,00E-008 (18,760023 %)
	Resistance 1	111,10443	0,2960082 (0,2664234 %)
	CPE Q 1	8,74E-009	2,29E-009 (26,180604 %)
	CPE Alpha 1	0,7736896	0,0165704 (2,1417409 %)
	CPE Q 2	5,16E-007	6,39E-009 (1,2372200 %)
	CPE Alpha 2	0,9274972	0,0013629 (0,1469436 %)

RelaxIS 3.0.20.16 - Report

Datasource: 9.5C_2.9_1

Circuit: I-(R)(P)-P

Type	Value
**Temperature:**	9,5000000
**Free variable:**	0,1000000
**DC Voltage:**	N/A
**AC Voltage:**	N/A
**Time:**	46,462219
**Harmonic:**	N/A
**Free Variable**	N/A

**2:**	
**Area:**	N/A
**Thickness:**	N/A

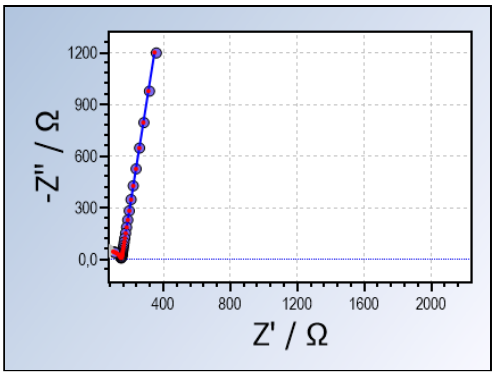

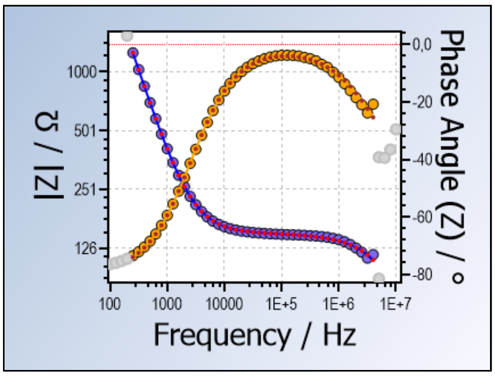

Fit Parameters:

Fix?	Name	Value	Error (Relative)
	Inductance 1	9,16E-008	9,59E-008 (104,73857 %)
	Resistance 1	149,53717	0,6049274 (0,4045331 %)
	CPE Q 1	6,59E-009	2,25E-009 (34,095464 %)
	CPE Alpha 1	0,7850803	0,0214311 (2,7297936 %)
	CPE Q 2	1,11E-006	2,76E-008 (2,4790773 %)
	CPE Alpha 2	0,8966281	0,0028477 (0,3175958 %)

RelaxIS 3.0.20.16 - Report

Datasource: 9.5C_3.0_1

Circuit: I-(R)(P)-P

Type	Value
**Temperature:**	9,5000000
**Free variable:**	0,0
**DC Voltage:**	N/A
**AC Voltage:**	N/A
**Time:**	47,961785
**Harmonic:**	N/A
**Free Variable 2:**	N/A
**Area:**	N/A
**Thickness:**	N/A

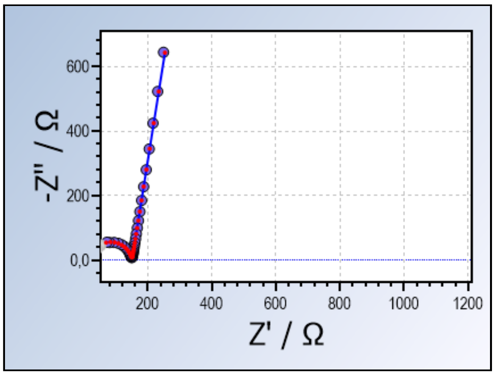

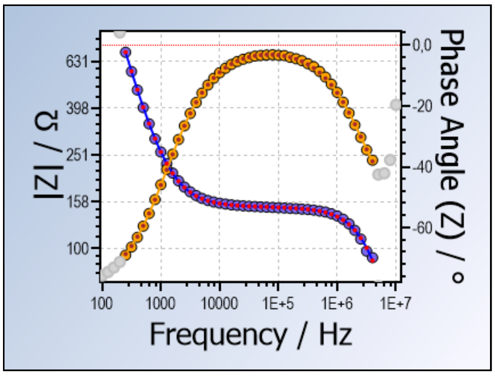

Fit Parameters:

Fix?	Name	Value	Error (Relative)
	Inductance 1	3,63E-007	2,48E-008 (6,8482922 %)
	Resistance 1	150,54455	0,2131289 (0,1415720 %)
	CPE Q 1	1,64E-009	1,23E-010 (7,4774335 %)
	CPE Alpha 1	0,8979396	0,0045826 (0,5103492 %)
	CPE Q 2	2,04E-006	2,49E-008 (1,2208364 %)
	CPE Alpha 2	0,8991354	0,0014441 (0,1606067 %)
