# Supplementary figures and images for: Re-investigating the structure–property relationship of the solid electrolytes Li 3−xIn1−xZrxCl6 and the impact of In–Zr(iv) substitution
Source: J Mater Chem A Mater. 2023 Jan 25;11(9):4559–71. doi: 10.1039/d2ta08433c (PMC9969333; doi:10.1039/d2ta08433c)

## Slide 1
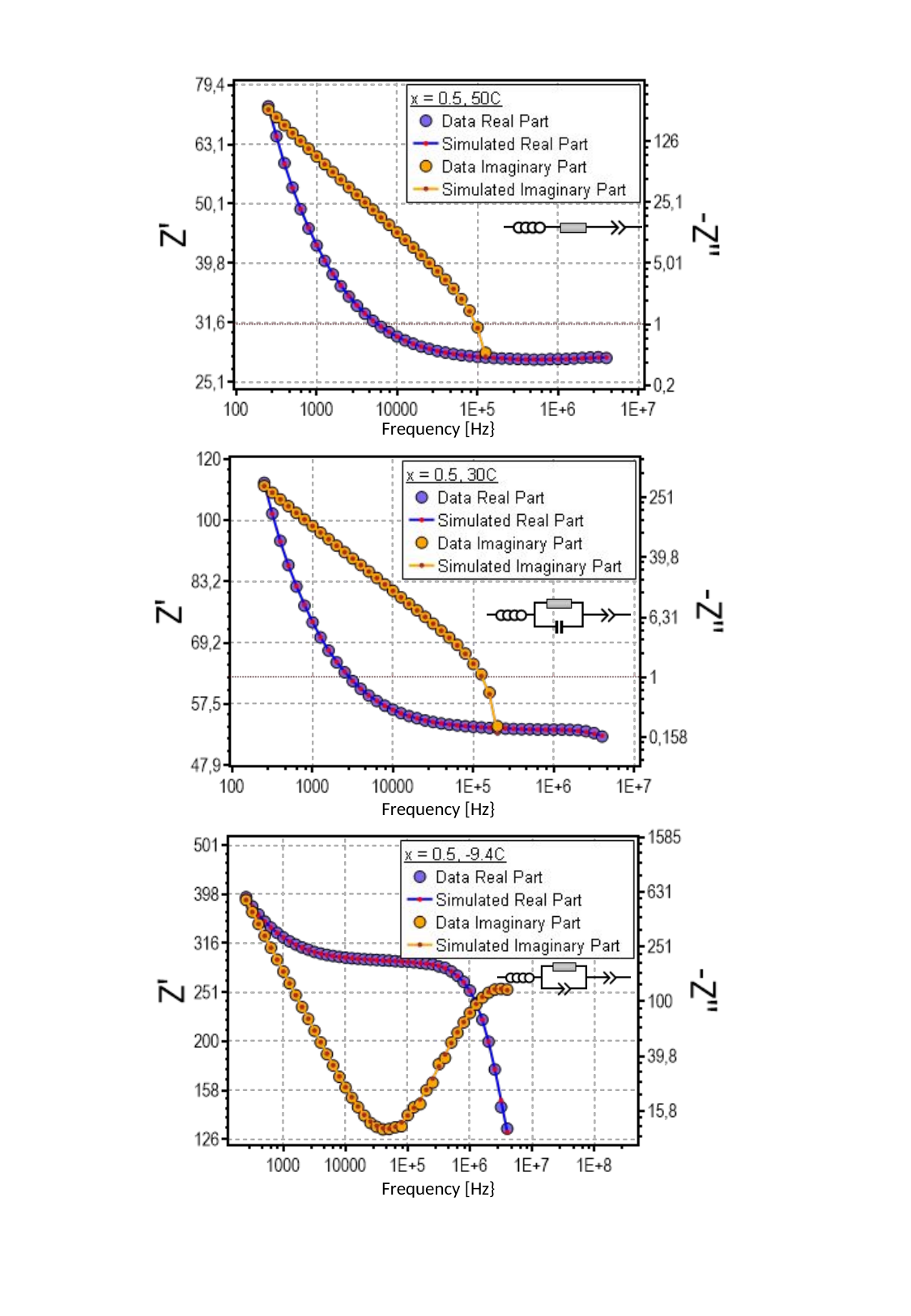

Frequency [Hz}
Frequency [Hz}
Frequency [Hz}

Supplement: TA-011-D2TA08433C-s003 [file TA-011-D2TA08433C-s003.zip › Kramers_Kroenig_impedance_error/Kramers_Kroenig_relationships.pptx]
